# Safety profile of entrectinib in NSCLC: Multi-source pharmacovigilance analysis using FAERS and JADER

**DOI:** 10.1371/journal.pone.0358331

**Published:** 2026-09-15

**Authors:** JiaLe Yang, JiaWen Liu, GuanBo Zhao, HeChen Li, Ge Sun

**Affiliations:** 1 Department of Thoracic Surgery, The Second Hospital of Dalian Medical University, Dalian, China; 2 China Medical University, Shenyang, China; 3 Dalian Medical University, Dalian, China; University of Kansas Medical Center, UNITED STATES OF AMERICA

## Abstract

**Background:**

Entrectinib is effective for ROS1-positive non-small cell lung cancer (NSCLC), but its postmarketing safety profile remains incompletely characterized outside clinical trials. We conducted a dual-database pharmacovigilance study to characterize real-world reporting patterns, identify cross-database replicated adverse event signals, explore age- and sex-related reporting heterogeneity, and evaluate time-to-onset patterns.

**Methods:**

Reports were retrieved from the U.S. Food and Drug Administration Adverse Event Reporting System (FAERS; 2020Q1–2025Q4; 520 patients; 1,573 preferred-term [PT] events) and the Japanese Adverse Drug Event Report database (JADER; 2020Q1–2025Q3; 254 patients; 393 PT events). Molecular fusion status was not available for verification. Four disproportionality methods were applied, with reporting odds ratio (ROR) as the primary signal criterion.

**Results:**

At the system organ class level, shared positive signals involved nervous system disorders (FAERS/JADER ROR: 4.48/4.76), cardiac disorders (3.20/5.43), and renal and urinary disorders (2.04/3.92). At the PT level, 23 signals were detected in both databases, whereas 58 PTs were unique to FAERS and 8 to JADER. Representative shared PT signals included dizziness (12.94/14.33), taste disorder (38.03/13.28), renal impairment (6.89/8.18), blood creatinine increased (8.31/24.99), cardiac failure (7.57/8.90), cognitive disorder (18.04/78.29), ataxia (51.73/351.45), syncope (8.87/89.45), myocarditis (3.47/5.91), electrocardiogram QT prolonged (4.01/5.35), and hyperuricaemia (7.36/59.63). Subgroup analyses suggested exploratory age- and sex-related reporting heterogeneity, including relatively more renal and mobility-related reports in older patients and female predominance for ataxia in FAERS. In the FAERS-based time-to-onset analysis, 203 of 520 reports (39.0%) had valid onset data. The median time to onset was 13 days, 70.94% of evaluable reports had onset dates within 30 days, and Weibull analysis suggested an early-failure pattern.

**Conclusions:**

Entrectinib-associated reports in NSCLC showed reproducible neurologic, cardiac, renal, and laboratory-related disproportional reporting signals across FAERS and JADER. These findings may help prioritize early safety monitoring but should be interpreted as hypothesis-generating signals rather than evidence of incidence or causality.

## 1 Introduction

Lung cancer remains the leading cause of cancer-related mortality worldwide, with non-small cell lung cancer (NSCLC) accounting for approximately 85%–90% of all cases [1]. ROS1 fusion occurs in approximately 1%–2% of NSCLC cases and defines a clinically actionable molecular subset [2,3]. The development of entrectinib, a potent ROS1-targeted tyrosine kinase inhibitor, has expanded therapeutic options for ROS1-positive NSCLC and improved disease control, particularly in patients with intracranial involvement [3,4]. This agent has demonstrated clinically meaningful activity in both treatment-naïve and previously treated patients with advanced disease [4].

Despite these advances, the safety profile of ROS1 inhibitors, particularly entrectinib, warrants further investigation [3,4]. Clinical trials provide important safety data [5]; however, they are typically conducted in selected patient populations and may not fully capture the spectrum of adverse events (AEs) observed in real-world practice [6].

To address this gap, we analyzed pharmacovigilance data from the U.S. Food and Drug Administration Adverse Event Reporting System (FAERS) and the Japanese Adverse Drug Event Report (JADER) database [7,8]. These systems provide complementary postmarketing data from distinct reporting settings and may facilitate the identification of safety signals that are not readily captured in clinical trials [9].

Building upon these considerations, we conducted a multi-source pharmacovigilance study to move beyond descriptive signal listing from a single spontaneous reporting system [10]. By integrating complementary data from FAERS (2020Q1–2025Q4) and JADER (2020Q1–2025Q3), we aimed to characterize entrectinib-associated adverse events at both the system organ class (SOC) and preferred term (PT) levels using four disproportionality methods—reporting odds ratio (ROR), proportional reporting ratio (PRR), Bayesian confidence propagation neural network (BCPNN), and multi-item gamma Poisson shrinker (MGPS). We further examined time-to-onset patterns using a Weibull distribution model and explored age- and sex-related heterogeneity through stratified subgroup analyses. Our aim was to identify reproducible pharmacovigilance signals associated with entrectinib, distinguish them from database-specific reporting fluctuations, and provide hypothesis-generating evidence to support earlier and more structured safety monitoring in routine practice [11].

## 2 Methods

### 2.1 Data source and collection

The FAERS is one of the largest spontaneous adverse event reporting systems worldwide [12]. It collects and stores adverse event (AE) reports submitted by healthcare professionals, patients, and manufacturers. As a key resource for postmarketing pharmacovigilance, FAERS supports drug safety monitoring in routine clinical practice and provides safety data across diverse populations and settings. The database comprises multiple tables, including demographics (DEMO), drug information (DRUG), adverse reactions (REAC), patient outcomes (OUTC), report sources (RPSR), therapy start and end dates (THER), and indications (INDI); these tables are linked using the unique CASEID identifier [13].

The JADER database is maintained by the Pharmaceuticals and Medical Devices Agency (PMDA) of Japan [8]. It collects adverse drug event reports submitted by pharmaceutical companies and medical institutions and serves as an important resource for postmarketing drug safety surveillance in Japan. JADER consists of four datasets: demographics (DEMO), drug information (DRUG), adverse reactions (REAC), and patient history (HIST) [14]. These datasets were linked using the unique case identifier provided in JADER to construct unified case-level records before subsequent filtering and analysis.

We conducted a multi-source pharmacovigilance analysis using FAERS data from 2020Q1 to 2025Q4 and JADER data from 2020Q1 to 2025Q3. The FAERS quarterly data files were downloaded on March 15, 2026, and the JADER data files were downloaded on March 15, 2026. In FAERS, reports were retrieved using the generic name entrectinib and the brand name Rozlytrek, with entrectinib designated as the primary suspect drug and the indication restricted to non-small cell lung cancer (NSCLC). In JADER, reports were retrieved using the Japanese drug name “エヌトレクチニブ”, with entrectinib likewise designated as the primary suspect drug and the indication restricted to NSCLC cases.

For FAERS, duplicate reports were addressed according to the FDA-recommended deduplication procedure, which prioritizes the most recent report version for each case on the basis of the unique CASEID and FDA_DT field, that is, the date on which the report was received by the FDA, to ensure retention of only the latest and most complete record [15]. No duplicate FAERS reports were ultimately retained after screening. This process yielded 520 eligible patients and 1,573 PT events. For JADER, duplicate records were removed before database linkage, resulting in 254 unique patients. After matching, a total of 393 PT events were identified.

**Fig 1:**
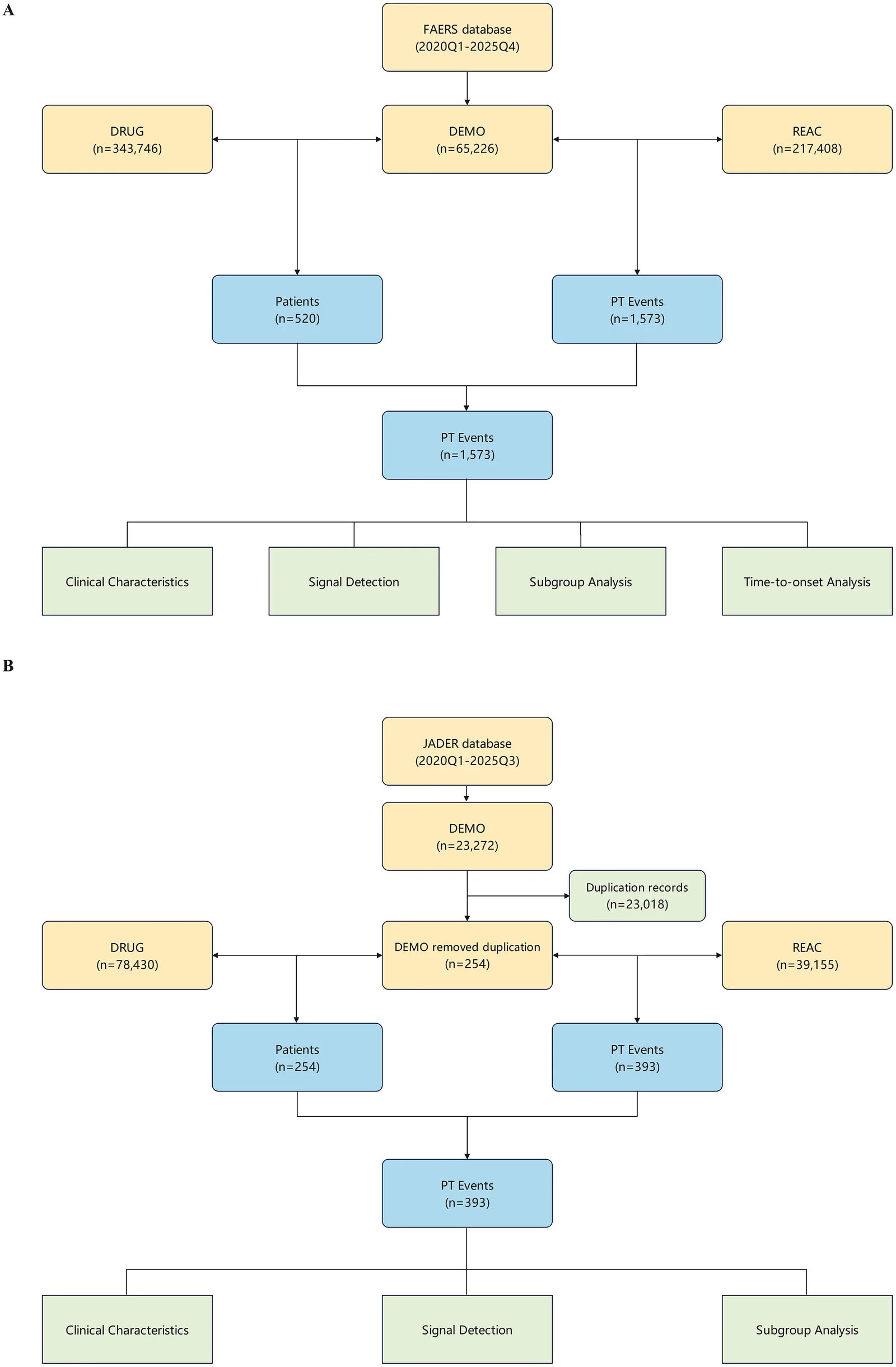
Data processing flowchart for entrectinib reports in FAERS and JADER. Panel A shows the data processing workflow for FAERS; panel B shows the data processing workflow for JADER. Abbreviations: FAERS, FDA Adverse Event Reporting System; JADER, Japanese Adverse Drug Event Report database; DEMO, demographics; DRUG, drug information; REAC, adverse reactions; PT, preferred term.

All AEs were coded according to the Medical Dictionary for Regulatory Activities (MedDRA), Version 28.1. AEs were mapped to PTs and corresponding SOCs for subsequent analyses. Because spontaneous reporting systems include both toxicity-related terms and disease-course-related terms, PTs such as “disease progression” were retained in the initial signal-detection output when they met the statistical criteria, but were categorized as disease-course-related reporting terms rather than toxicity-specific AE signals for clinical interpretation. These terms were therefore not considered part of the core toxicity profile of entrectinib. A schematic overview of the study workflow is presented in Fig 1.

Because this study used publicly available and de-identified pharmacovigilance data from FAERS and JADER, it did not involve direct contact with human participants or access to identifiable individual-level patient information. Therefore, approval from an institutional ethics review committee and informed consent were not required.

### 2.2 Statistical analysis

Four established disproportionality methods—reporting odds ratio (ROR) [15], proportional reporting ratio (PRR) [16], Bayesian confidence propagation neural network (BCPNN) [17], and multi-item gamma Poisson shrinker (MGPS) [18] —were applied for signal detection. ROR was selected as the primary criterion for defining positive signals because it is widely used in spontaneous reporting system analyses, is straightforward to interpret, and allows comparison with previous pharmacovigilance studies [19]. PRR, BCPNN, and MGPS were used as complementary methods to evaluate the robustness of the detected signals under different disproportionality assumptions. Because these algorithms differ in statistical principles, shrinkage behavior, and sensitivity to sparse counts, complete overlap across methods was not expected. Therefore, signals meeting the ROR criterion were considered the primary signal set, whereas signals additionally supported by PRR, BCPNN, and/or MGPS were regarded as more robust. Conversely, PTs detected by only one method, particularly those based on sparse counts, were interpreted cautiously as exploratory and hypothesis-generating rather than definitive safety findings.

For each database, disproportionality analyses were performed within the NSCLC reporting background rather than against the entire spontaneous reporting database [20]. The target group consisted of reports in which entrectinib was recorded as the primary suspect drug for NSCLC-related indications. The reference set consisted of NSCLC-related reports involving all other drugs after excluding entrectinib-related reports. Therefore, the estimated RORs reflected disproportionate reporting of PTs for entrectinib relative to other drug reports within the same disease-reporting background, rather than relative to the entire FAERS or JADER database.

For each PT, a two-by-two contingency table was constructed separately in FAERS and JADER. In this event-level disproportionality analysis, “a” represented the number of target PT events in the entrectinib-NSCLC target group, “b” represented other PT events in the target group, “c” represented target PT events in the reference set, and “d” represented other PT events in the reference set [21]. The event-level contingency table structure and PT event totals are shown in S1 Table. A PT was considered a positive signal when the number of reported events was at least 3 (a ≥ 3) and the lower bound of the 95% confidence interval (CI) for the ROR exceeded 1. Detailed formulas and signal-detection criteria for ROR, PRR, BCPNN, and MGPS are provided in S2 Table. Because disproportionality analysis is intended for pharmacovigilance signal detection rather than confirmatory hypothesis testing, no formal multiplicity adjustment was applied; therefore, signals detected in only one database, by only one method, or based on sparse counts were interpreted as exploratory and hypothesis-generating [22].

Particular caution was applied when interpreting low-frequency signals with extremely large RORs and wide confidence intervals. Although such signals were retained in the complete supplementary signal lists for transparency, PTs with sparse counts, especially those based on only 3–5 reports, were not emphasized as definitive safety findings in the main interpretation. These signals were considered statistically unstable and potentially sensitive to reporting variation, coding practices, and random fluctuation.

Descriptive analyses were first performed to summarize the characteristics of entrectinib-associated adverse event (AE) reports. Subgroup analyses were conducted according to sex and age categories as recorded in the source databases [23,24]. In FAERS, age was categorized as <65 years and ≥65 years, whereas in JADER, age was categorized as <70 years and ≥70 years according to the available age categories in the public dataset. Because the age cutoffs differed between the two databases, age-stratified findings were interpreted cautiously and were not used for direct cross-database comparison. Subgroup analyses were considered exploratory because several estimates were based on small numbers of reports and wide confidence intervals.

Time-to-onset (TTO) was defined as the interval between treatment initiation (START_DT) and adverse event (AE) onset (EVENT_DT). TTO analysis was restricted to FAERS because the number of JADER reports with complete and valid date-paired information was insufficient for reliable statistical analysis [25]. Only reports with complete and valid date information were included, whereas records with missing dates, implausible dates, or negative time intervals were excluded [26]. In FAERS, 203 of 520 eligible reports contained complete and valid onset-time data and were retained for TTO analysis, corresponding to 39.0% of eligible reports. The remaining 317 reports were excluded because of missing or invalid date information.

A Weibull distribution model was applied to characterize the temporal reporting pattern of AE onset [27]. The scale parameter (α), shape parameter (β), and corresponding 95% confidence intervals were estimated. Failure patterns were classified according to the β estimate and its 95% confidence interval: early failure was defined as β < 1 with an upper 95% confidence limit below 1; random failure was defined when the 95% confidence interval included 1; and wear-out failure was defined as β > 1 with a lower 95% confidence limit above 1 [24]. All data processing, statistical analyses, and figure generation were performed using R software (version 4.5.2).

## 3 Results

### 3.1 Descriptive analysis

A total of 520 eligible patients from FAERS and 254 unique patients from JADER were included in the descriptive analysis, and baseline characteristics are presented in Tables 1 and 2. In both databases, female patients accounted for a greater proportion than male patients, representing 48.7% and 57.9% of the study population in FAERS and JADER, respectively. Sex data were missing more frequently in FAERS than in JADER (10.4% vs. 4.7%).

**Table 1:** Baseline characteristics of entrectinib-associated adverse event reports in the FAERS database.

	Number	Proportion (%)
**Gender**		
Female	253	48.7%
Male	213	41.0%
Missing	54	10.4%
**WT**		
<50 kg	32	6.2%
>100 kg	4	0.8%
50 ~ 100 kg	129	24.8%
missing	355	68.3%
**AGE**		
<65	193	37.1%
≥65	204	39.2%
Missing	123	23.7%
**Outcome**		
Death	77	14.8%
Disability	4	0.8%
Hospitalization	86	16.5%
Life-Threatening	16	3.1%
Other	337	64.8%
**Reporter**		
Consumer	52	10.0%
Health Professional	52	10.0%
Pharmacist	42	8.1%
Physician	372	71.5%
Missing	2	0.4%

Note: Data are presented as number and proportion unless otherwise indicated. Percentages were calculated using the total number of eligible FAERS patients as the denominator (n = 520). Missing values were retained as a separate category where applicable. Abbreviations: FAERS, FDA Adverse Event Reporting System; WT, body weight.

**Table 2:** Baseline characteristics of entrectinib-associated adverse event reports in the JADER database.

	Number	Proportion (%)
**Gender**		
Female	147	57.9%
Male	95	37.4%
Missing	12	4.7%
**WT**		
<50 kg	39	15.4%
>100 kg	2	0.8%
50 ~ 100 kg	94	37.0%
Missing	119	46.9%
**AGE**		
<70	146	57.5%
≥70	105	41.3%
Missing	3	1.2%
Outcome*		
Recovered	147	37.4%
Partially Recovered	117	29.8%
Not Recovered	50	12.7%
Sequelae Present	1	0.3%
Death	25	6.4%
Other	53	13.5%
**Reporter**		
Health Professional	4	1.6%
Pharmacist	47	18.5%
Physician	203	79.9%

Note: Data are presented as number and proportion unless otherwise indicated. Percentages for sex, body weight, age, and reporter type were calculated using the total number of eligible JADER patients as the denominator (n = 254), whereas clinical outcome percentages were calculated using the total number of PT-level adverse event records as the denominator (n = 393). Missing values were retained as a separate category where applicable. Abbreviations: JADER, Japanese Adverse Drug Event Report database; PT, preferred term; WT, body weight.

Outcome* percentages were calculated using 393 PT-level adverse event records as the denominator.

Body weight data were incomplete in both databases, particularly in FAERS. Missing body weight data were recorded in 68.3% of FAERS patients and 46.9% of JADER patients. Among patients with available data, the 50–100 kg category represented the largest subgroup in both databases, accounting for 24.8% in FAERS and 37.0% in JADER.

The age distribution differed modestly between the two databases. In FAERS, patients aged ≥65 years accounted for 39.2% of the cohort, slightly exceeding the proportion of those aged <65 years (37.1%), whereas 23.7% of patients had missing age data. In JADER, patients aged <70 years constituted the majority (57.5%), whereas those aged ≥70 years accounted for 41.3%; only 1.2% of patients had missing age data.

Outcome profiles also differed between the two databases. In FAERS, the most frequently reported outcome was “other” (64.8%), followed by hospitalization (16.5%) and death (14.8%). Life-threatening events and disability were infrequently reported, accounting for 3.1% and 0.8%, respectively. In JADER, recovered and partially recovered outcomes were the most common, accounting for 37.4% and 29.8%, respectively. Not recovered outcomes accounted for 12.7%, death for 6.4%, and sequelae for 0.3%.

Physicians were the predominant reporters in both databases, contributing 71.5% of reports in FAERS and 79.9% in JADER. Pharmacists accounted for 8.1% of reports in FAERS and 18.5% in JADER, whereas consumers and other healthcare professionals accounted for smaller proportions overall. Overall, physician-reported cases constituted the primary source of entrectinib-related adverse event reporting in both databases.

**Fig 2:**
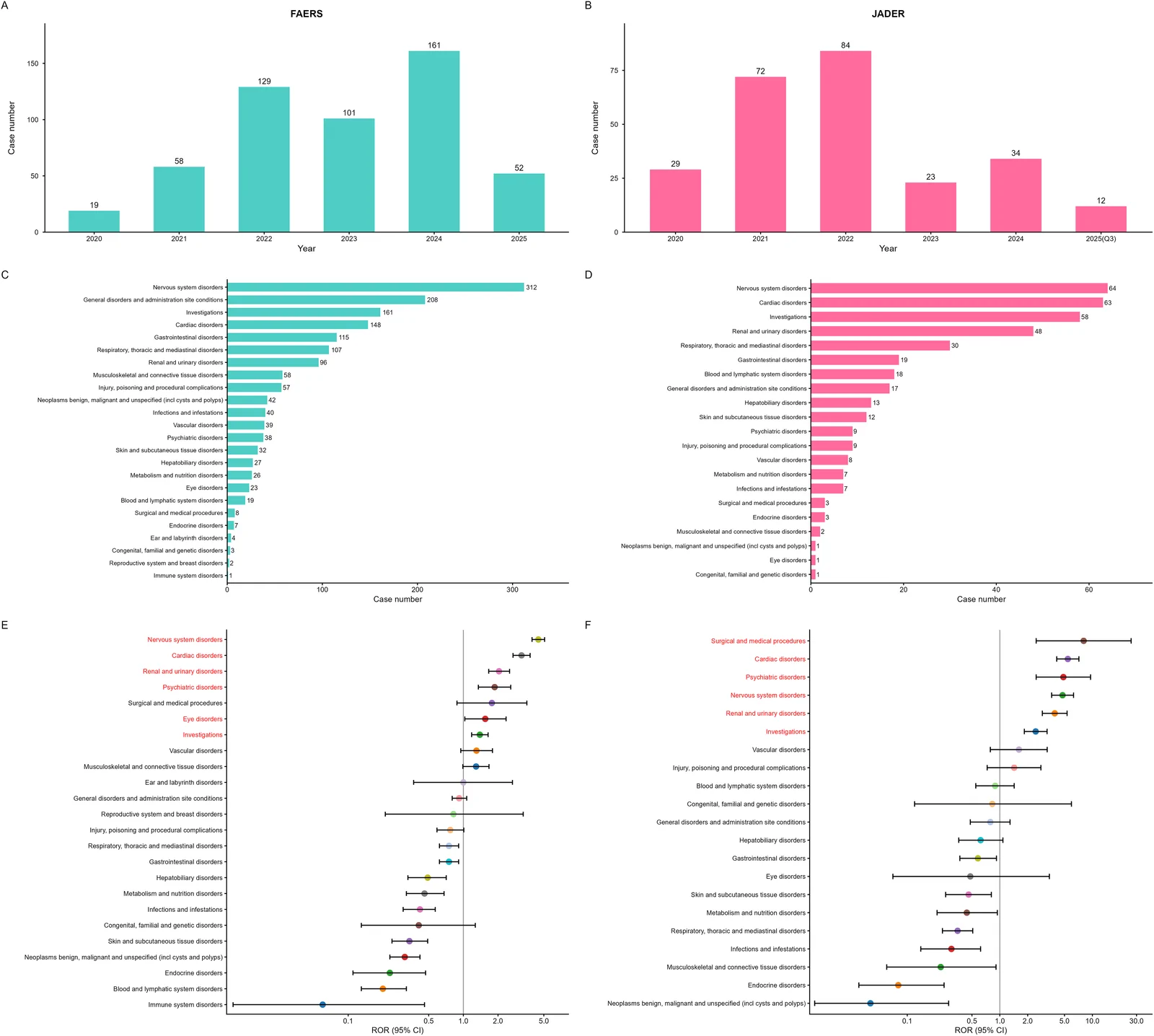
Annual trends and SOC-level signal profiles in FAERS and JADER. Panels A and B show annual reporting trends in FAERS and JADER, respectively. Panels C and D show the distribution of adverse events by SOC in FAERS and JADER, respectively. Panels E and F show SOC-level reporting odds ratios with 95% confidence intervals in FAERS and JADER, respectively. Abbreviations: SOC, system organ class; FAERS, FDA Adverse Event Reporting System; JADER, Japanese Adverse Drug Event Report database; ROR, reporting odds ratio; CI, confidence interval.

The annual reporting trends are shown in Figs 2A and 2B. In FAERS, the number of eligible reports increased from 19 in 2020–58 in 2021 and 129 in 2022, remained elevated in 2023 (101), peaked in 2024 (161), and declined in 2025 (52). In JADER, the corresponding numbers were 29 in 2020, 72 in 2021, 84 in 2022, 23 in 2023, 34 in 2024, and 12 in 2025Q3. Taken together, the yearly distributions describe reporting activity over time in the two databases, with an early postmarketing increase followed by subsequent fluctuations. These trends should be interpreted as reporting patterns rather than changes in the true incidence of adverse events.

### 3.2 Signal detection

The SOC distribution of entrectinib-associated adverse events is shown in Figs 2C and 2D, and the corresponding SOC-level disproportionality profiles are presented in Figs 2E and 2F. In FAERS, nervous system disorders were the most frequently reported SOC (n = 312), followed by general disorders and administration site conditions (n = 208), investigations (n = 161), cardiac disorders (n = 148), gastrointestinal disorders (n = 115), respiratory, thoracic and mediastinal disorders (n = 107), and renal and urinary disorders (n = 96). In JADER, the leading SOCs included nervous system disorders (n = 64), cardiac disorders (n = 63), investigations (n = 58), renal and urinary disorders (n = 48), and respiratory, thoracic and mediastinal disorders (n = 30).

At the SOC level, nervous system disorders exhibited the strongest positive signal in both databases, with an ROR of 4.48 (95% CI 3.95–5.08) in FAERS and 4.76 (95% CI 3.63–6.26) in JADER. In FAERS, additional positive SOC-level signals were identified for cardiac disorders (ROR 3.20, 95% CI 2.70–3.80), renal and urinary disorders (ROR 2.04, 95% CI 1.66–2.52), psychiatric disorders (ROR 1.87, 95% CI 1.35–2.58), investigations (ROR 1.39, 95% CI 1.18–1.64), and eye disorders (ROR 1.55, 95% CI 1.03–2.35). In JADER, positive signals were also observed for cardiac disorders (ROR 5.43, 95% CI 4.12–7.15), renal and urinary disorders (ROR 3.92, 95% CI 2.88–5.33), investigations (ROR 2.44, 95% CI 1.84–3.24), psychiatric disorders (ROR 4.86, 95% CI 2.47–9.56), and surgical and medical procedures (ROR 8.05, 95% CI 2.47–26.22). The complete SOC-level disproportionality results are provided in S3 Table.

**Fig 3:**
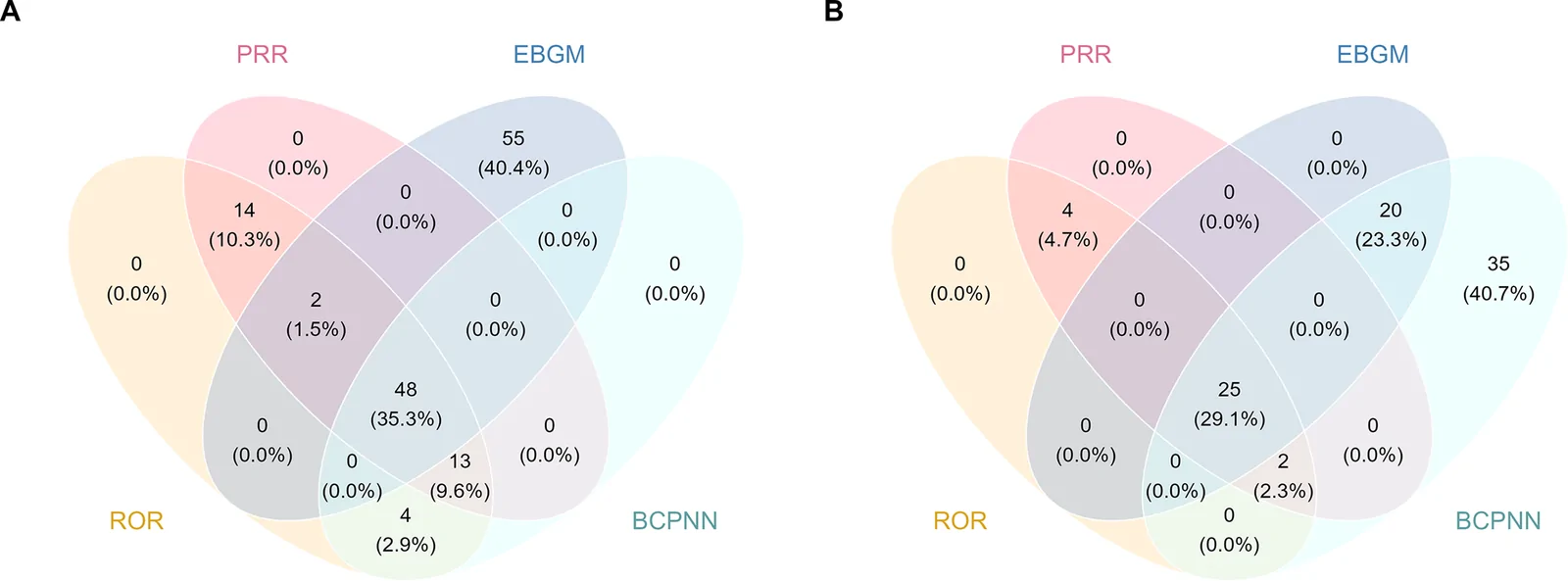
Overlap of positive PT signals across four disproportionality algorithms. Panel A shows the overlap of positive PT signals in FAERS; panel B shows the overlap of positive PT signals in JADER. The Venn diagrams summarize the overlap among the signal detection results obtained using ROR, PRR, BCPNN, and MGPS, with MGPS represented by the EBGM. Abbreviations: PT, preferred term; FAERS, FDA Adverse Event Reporting System; JADER, Japanese Adverse Drug Event Report database; ROR, reporting odds ratio; PRR, proportional reporting ratio; BCPNN, Bayesian confidence propagation neural network; MGPS, multi-item gamma Poisson shrinker; EBGM, empirical Bayes geometric mean.

The overlap among the four disproportionality methods is shown in Figs 3A and 3B. In Fig 3A (FAERS), 48 PTs were simultaneously detected by all four methods; 55 PTs were unique to EBGM, 14 were shared by PRR and ROR, 13 by BCPNN and EBGM, 2 by PRR, ROR, and EBGM, and 4 by ROR, EBGM, and BCPNN. In Fig 3B (JADER), 25 PTs overlapped across all four algorithms; 35 PTs were unique to BCPNN, 20 were shared only between BCPNN and EBGM, 4 were shared by PRR and ROR, and 2 were shared by ROR, EBGM, and BCPNN. These findings indicate that a subset of PT signals was consistently detected across multiple methods, whereas other signals were method-specific and therefore required more cautious interpretation.

**Fig 4:**
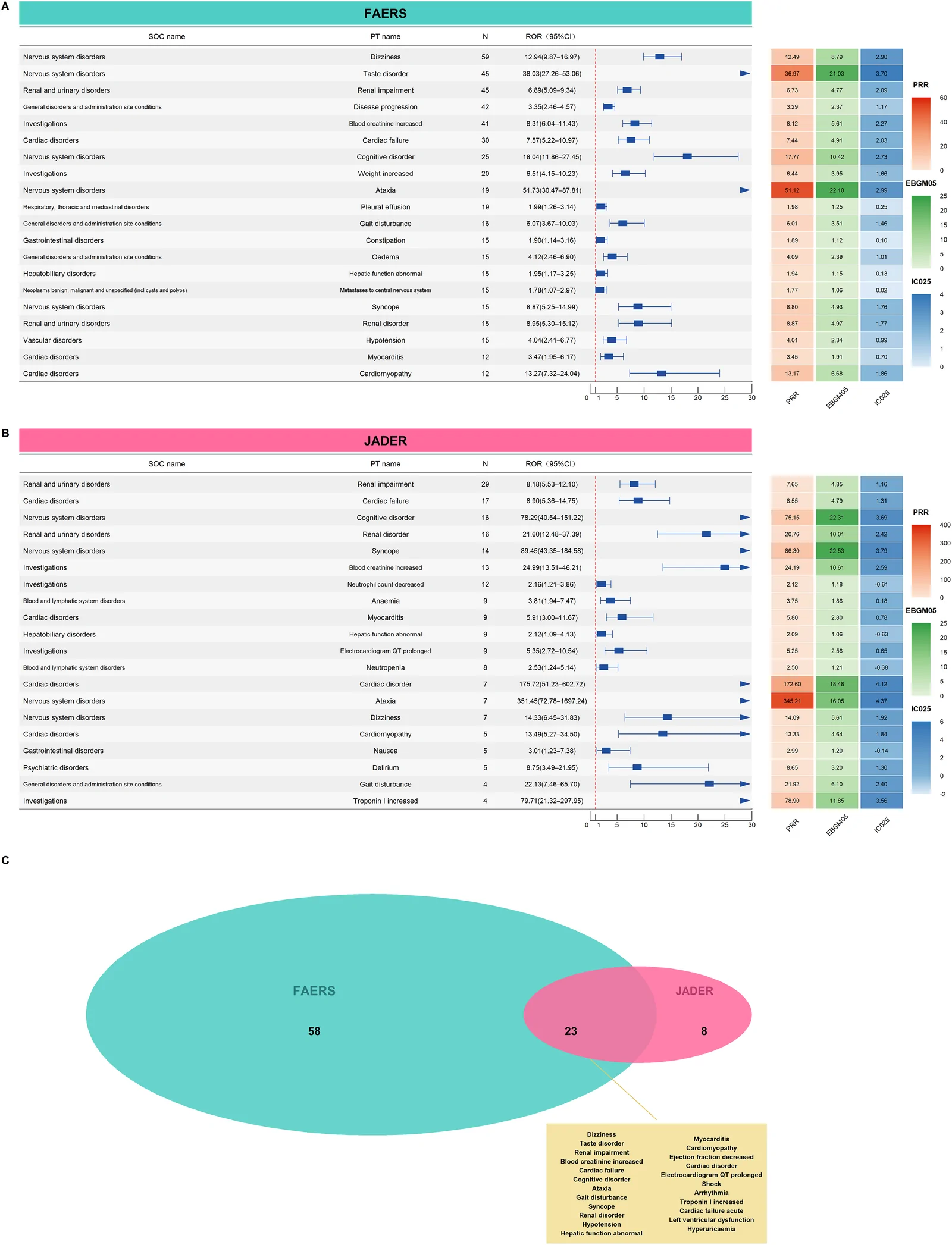
PT-level signals and cross-database overlap in FAERS and JADER. Panels A and B show representative PT-level signals in FAERS and JADER, respectively. The n (%) column indicates the number and proportion of PT events among all PT-level adverse event records in the corresponding target group (FAERS, n = 1,573; JADER, n = 393). In the forest plots, squares represent ROR point estimates, horizontal lines represent 95% confidence intervals, and the vertical dashed line represents an ROR of 1. Arrows indicate that the upper confidence limit extends beyond the displayed plotting range. The adjacent heatmaps show PRR, EBGM05, and IC025 values as complementary disproportionality metrics. Panel C shows 58 PT signals detected only in FAERS, 23 signals detected in both databases, and 8 signals detected only in JADER; the accompanying box lists the 23 cross-database replicated PT signals. Complete PT-level signal results are provided in S4 Table for FAERS and S5 Table for JADER. Abbreviations: PT, preferred term; SOC, system organ class; FAERS, FDA Adverse Event Reporting System; JADER, Japanese Adverse Drug Event Report database; ROR, reporting odds ratio; CI, confidence interval; PRR, proportional reporting ratio; EBGM05, lower fifth percentile of the empirical Bayes geometric mean; IC025, lower limit of the 95% credibility interval for the information component.

The major positive PT-level signals are presented in Figs 4A and 4B, whereas the complete PT-level signal lists are provided in S4 Table for FAERS and S5 Table for JADER. In FAERS, the most frequent and clinically notable PTs were dizziness (n = 59; ROR 12.94, 95% CI 9.87–16.97), taste disorder (n = 45; ROR 38.03, 95% CI 27.26–53.06), renal impairment (n = 45; ROR 6.89, 95% CI 5.09–9.34), disease progression (n = 42; ROR 3.35, 95% CI 2.46–4.57), blood creatinine increased (n = 41; ROR 8.31, 95% CI 6.04–11.43), cardiac failure (n = 30; ROR 7.57, 95% CI 5.22–10.97), and cognitive disorder (n = 25; ROR 18.04, 95% CI 11.86–27.45). Other notable PTs in the main figure included weight increased (n = 20; ROR 6.51, 95% CI 4.15–10.23), ataxia (n = 19; ROR 51.73, 95% CI 30.47–87.81), syncope (n = 15; ROR 8.87, 95% CI 5.25–14.99), renal disorder (n = 15; ROR 8.95, 95% CI 5.30–15.12), hypotension (n = 15; ROR 4.04, 95% CI 2.41–6.77), myocarditis (n = 12; ROR 3.47, 95% CI 1.95–6.17), and cardiomyopathy (n = 12; ROR 13.27, 95% CI 7.32–24.04). In JADER, the leading PT signals included renal impairment (n = 29; ROR 8.18, 95% CI 5.53–12.10), cardiac failure (n = 17; ROR 8.90, 95% CI 5.36–14.75), renal disorder (n = 16; ROR 21.60, 95% CI 12.48–37.39), cognitive disorder (n = 16; ROR 78.29, 95% CI 40.54–151.22), syncope (n = 14; ROR 89.45, 95% CI 43.35–184.58), and blood creatinine increased (n = 13; ROR 24.99, 95% CI 13.51–46.21). Additional notable PTs in the main figure included neutrophil count decreased (n = 12; ROR 2.16, 95% CI 1.21–3.86), electrocardiogram QT prolonged (n = 9; ROR 5.35, 95% CI 2.72–10.54), hepatic function abnormal (n = 9; ROR 2.12, 95% CI 1.09–4.13), myocarditis (n = 9; ROR 5.91, 95% CI 3.00–11.67), anaemia (n = 9; ROR 3.81, 95% CI 1.94–7.47), neutropenia (n = 8; ROR 2.53, 95% CI 1.24–5.14), ataxia (n = 7; ROR 351.45, 95% CI 72.78–1697.24), dizziness (n = 7; ROR 14.33, 95% CI 6.45–31.83), and cardiac disorder (n = 7; ROR 175.72, 95% CI 51.23–602.72).

Beyond the more frequent PTs highlighted in the main figure, the supplementary tables identified several low-frequency yet disproportionately strong signals. In FAERS, representative examples from S4 Table included dyslalia (n = 4; ROR 91.71, 95% CI 25.85–325.29), electrocardiogram T wave inversion (n = 3; ROR 82.48, 95% CI 19.69–345.44), ligament rupture (n = 3; ROR 206.21, 95% CI 34.43–1234.98), joint effusion (n = 3; ROR 41.24, 95% CI 11.34–150.00), urinary incontinence (n = 5; ROR 14.04, 95% CI 5.59–35.29), and orthostatic hypotension (n = 6; ROR 15.89, 95% CI 6.81–37.05). In JADER, S5 Table showed similarly strong but less frequently reported signals, including ataxia (n = 7; ROR 351.45, 95% CI 72.78–1697.24), cardiac disorder (n = 7; ROR 175.72, 95% CI 51.23–602.72), troponin I increased (n = 4; ROR 79.71, 95% CI 21.32–297.95), left ventricular dysfunction (n = 3; ROR 99.38, 95% CI 20.00–493.94), hyperuricaemia (n = 3; ROR 59.63, 95% CI 14.20–250.37), and arrhythmia (n = 3; ROR 11.92, 95% CI 3.58–39.64). These low-frequency signals were retained in the supplementary tables for transparency; however, because several were based on sparse counts and wide confidence intervals, they should be interpreted cautiously as exploratory pharmacovigilance signals rather than confirmed adverse drug reactions.

Cross-database comparison is summarized in Table 3 and Fig 4C. Twenty-three PT signals were identified in both FAERS and JADER, mainly involving neurologic, renal, cardiac, vascular, hepatic, metabolic, and laboratory-related events. Representative shared signals included dizziness, taste disorder, renal impairment, blood creatinine increased, cardiac failure, cognitive disorder, ataxia, syncope, renal disorder, myocarditis, cardiomyopathy, electrocardiogram QT prolonged, arrhythmia, troponin I increased, left ventricular dysfunction, and hyperuricaemia. Because these PTs were detected in both pharmacovigilance systems, they were considered more reproducible than database-specific signals. Detailed event counts and ROR estimates with 95% confidence intervals for all 23 shared PTs are provided in Table 3. In contrast, 58 positive PTs were unique to FAERS and 8 were unique to JADER.

**Table 3:** Cross-database replicated PT-level signals of entrectinib-associated adverse event reports in FAERS and JADER.

	FAERS		JADER	
PT	n (%)	ROR (95% CI)	n (%)	ROR (95% CI)
Dizziness	59 (3.75%)	12.94 (9.87–16.97)	7 (1.78%)	14.33 (6.45–31.83)
Taste disorder	45 (2.86%)	38.03 (27.26–53.06)	4 (1.02%)	13.28 (4.65–37.86)
Renal impairment	45 (2.86%)	6.89 (5.09–9.34)	29 (7.38%)	8.18 (5.53–12.10)
Blood creatinine increased	41 (2.61%)	8.31 (6.04–11.43)	13 (3.31%)	24.99 (13.51–46.21)
Cardiac failure	30 (1.91%)	7.57 (5.22–10.97)	17 (4.33%)	8.90 (5.36–14.75)
Cognitive disorder	25 (1.59%)	18.04 (11.86–27.45)	16 (4.07%)	78.29 (40.54–151.22)
Ataxia	19 (1.21%)	51.73 (30.47–87.81)	7 (1.78%)	351.45 (72.78–1697.24)
Gait disturbance	16 (1.02%)	6.07 (3.67–10.03)	4 (1.02%)	22.13 (7.46–65.70)
Syncope	15 (0.95%)	8.87 (5.25–14.99)	14 (3.56%)	89.45 (43.35–184.58)
Renal disorder	15 (0.95%)	8.95 (5.30–15.12)	16 (4.07%)	21.60 (12.48–37.39)
Hypotension	15 (0.95%)	4.04 (2.41–6.77)	3 (0.76%)	11.04 (3.33–36.53)
Hepatic function abnormal	15 (0.95%)	1.95 (1.17–3.25)	9 (2.29%)	2.12 (1.09–4.13)
Myocarditis	12 (0.76%)	3.47 (1.95–6.17)	9 (2.29%)	5.91 (3.00–11.67)
Cardiomyopathy	12 (0.76%)	13.27 (7.32–24.04)	5 (1.27%)	13.49 (5.27–34.50)
Ejection fraction decreased	12 (0.76%)	12.19 (6.74–22.05)	4 (1.02%)	17.32 (5.96–50.32)
Cardiac disorder	10 (0.64%)	10.38 (5.45–19.77)	7 (1.78%)	175.72 (51.23–602.72)
Electrocardiogram qt prolonged	6 (0.38%)	4.01 (1.78–9.04)	9 (2.29%)	5.35 (2.72–10.54)
Shock	5 (0.32%)	8.94 (3.61–22.11)	3 (0.76%)	11.46 (3.45–38.02)
Arrhythmia	5 (0.32%)	4.81 (1.97–11.75)	3 (0.76%)	11.92 (3.58–39.64)
Troponin I increased	4 (0.25%)	28.96 (9.84–85.22)	4 (1.02%)	79.71 (21.32–297.95)
Cardiac failure acute	4 (0.25%)	6.63 (2.43–18.10)	3 (0.76%)	9.61 (2.93–31.57)
Left ventricular dysfunction	4 (0.25%)	6.71 (2.46–18.32)	3 (0.76%)	99.38 (20.00–493.94)
Hyperuricaemia	3 (0.19%)	7.36 (2.30–23.55)	3 (0.76%)	59.63 (14.20–250.37)

Note: Shared PT signals were defined as positive PT-level signals detected in both FAERS and JADER. Event counts indicate the number of reports for each PT in the entrectinib-NSCLC target group. RORs are presented with 95% confidence intervals. Percentages were calculated using the total number of PT-level adverse event records in the corresponding database as the denominator (FAERS, n = 1,573; JADER, n = 393). These percentages represent the distribution of reported PT events and should not be interpreted as incidence rates or patient-level risks. Abbreviations: PT, preferred term; FAERS, FDA Adverse Event Reporting System; JADER, Japanese Adverse Drug Event Report database; ROR, reporting odds ratio; CI, confidence interval; NSCLC, non-small cell lung cancer.

### 3.3 Subgroup analysis

**Fig 5:**
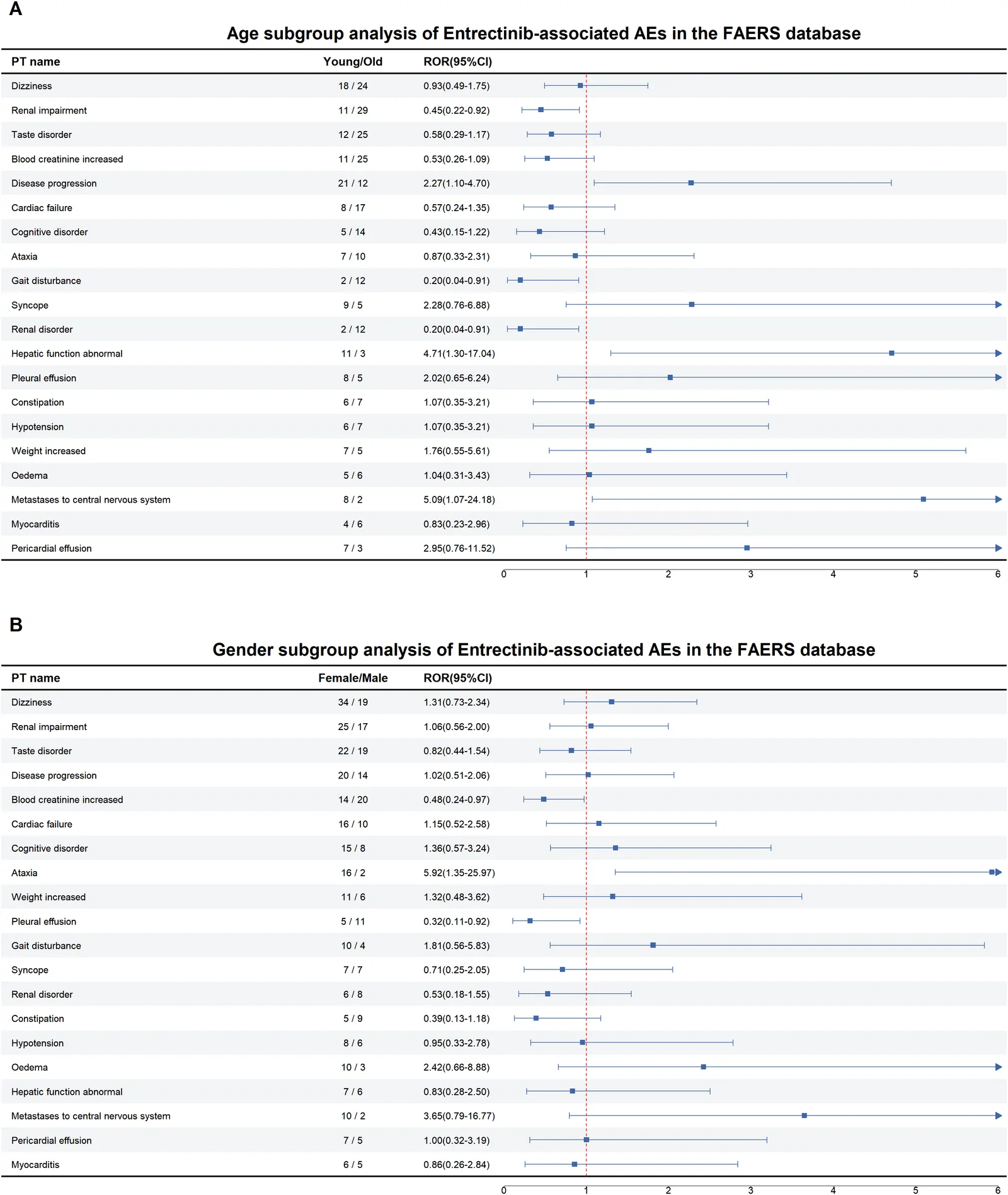
Subgroup analysis of positive PT signals in FAERS. Panel A compares patients aged <65 years with those aged ≥65 years; event counts are presented in the order <65/ ≥ 65 years, and RORs compare reporting in the younger group with reporting in the older group. Therefore, an ROR greater than 1 indicates relatively greater reporting in younger patients, whereas an ROR less than 1 indicates relatively greater reporting in older patients. Panel B compares female with male patients; event counts are presented in the order female/male, and RORs compare reporting in females with reporting in males. Therefore, an ROR greater than 1 indicates relatively greater reporting in females, whereas an ROR less than 1 indicates relatively greater reporting in males. Squares represent ROR point estimates, horizontal lines represent 95% confidence intervals, and the vertical dashed line represents an ROR of 1. Arrows indicate that the upper confidence limit extends beyond the displayed plotting range. These subgroup findings are exploratory and should be interpreted cautiously because several estimates are based on small event counts. Abbreviations: PT, preferred term; FAERS, FDA Adverse Event Reporting System; ROR, reporting odds ratio; CI, confidence interval.

Subgroup analyses are presented in Fig 5 and S6–S8 Tables. The main figure displays the age- and sex-stratified forest plots from FAERS. The complete age-stratified FAERS results are provided in S6 Table, and the complete sex-stratified FAERS results are provided in S7 Table. Sex-stratified results from JADER are summarized in S8 Table. Age-stratified subgroup analysis was not conducted for JADER because the publicly available dataset provides only broad age categories, and privacy-related masking in sparse strata could compromise the stability and interpretability of age-specific disproportionality estimates. Overall, subgroup findings were considered exploratory because several estimates were based on small numbers of reports and wide confidence intervals.

In the FAERS age-stratified analysis (Fig 5A), several PTs exhibited age-related heterogeneity. Signals relatively enriched in younger patients included disease progression (21 vs. 12 cases; ROR 2.27, 95% CI 1.10–4.70), hepatic function abnormal (11 vs. 3 cases; ROR 4.71, 95% CI 1.30–17.04), and metastases to central nervous system (8 vs. 2 cases; ROR 5.09, 95% CI 1.07–24.18). S6 Table also showed several PTs observed only in the younger subgroup, including seizure (6 vs. 0), blood creatine phosphokinase increased (5 vs. 0), pain of skin (2 vs. 0), and dyskinesia (2 vs. 0). By contrast, renal impairment (11 vs. 29 cases; ROR 0.45, 95% CI 0.22–0.92), gait disturbance (2 vs. 12 cases; ROR 0.20, 95% CI 0.04–0.91), and renal disorder (2 vs. 12 cases; ROR 0.20, 95% CI 0.04–0.91) were numerically more frequent in older patients; because younger patients served as the reference group, the corresponding RORs were less than 1. PTs observed only in the older subgroup included urinary incontinence (0 vs. 4), humerus fracture (0 vs. 3), contusion (0 vs. 3), and central nervous system lesion (0 vs. 2).

In the FAERS sex-stratified analysis (Fig 5B), ataxia showed the most pronounced female predominance (16 vs. 2 cases; ROR 5.92, 95% CI 1.35–25.97). In contrast, blood creatinine increased was less frequent in females than in males (14 vs. 20 cases; ROR 0.48, 95% CI 0.24–0.97), and pleural effusion similarly showed a male-predominant pattern (5 vs. 11 cases; ROR 0.32, 95% CI 0.11–0.92). S7 Table identified several PTs observed only in females, including memory impairment (6 vs. 0), aphasia (4 vs. 0), dyslalia (3 vs. 0), and cerebral haemorrhage (3 vs. 0). Conversely, several PTs were observed only in males, including seizure (0 vs. 8), troponin I increased (0 vs. 3), contusion (0 vs. 3), and electrocardiogram abnormal (0 vs. 3).

JADER sex-stratified findings are summarized in S8 Table. Although these estimates should be interpreted cautiously because of the limited sample size, several patterns were notable. Cognitive disorder was numerically more frequent in females than in males (13 vs. 3 cases; ROR 2.64, 95% CI 0.73–9.56), as were ataxia (6 vs. 1; ROR 3.78, 95% CI 0.45–31.93) and electrocardiogram QT prolonged (6 vs. 1; ROR 3.78, 95% CI 0.45–31.93). By contrast, cardiac failure was more common in males (5 vs. 10 cases; ROR 0.32, 95% CI 0.10–0.96), and blood creatinine increased appeared more frequent in males (5 vs. 8; ROR 0.40, 95% CI 0.13–1.25).

### 3.4 Time-to-onset analysis

**Fig 6:**
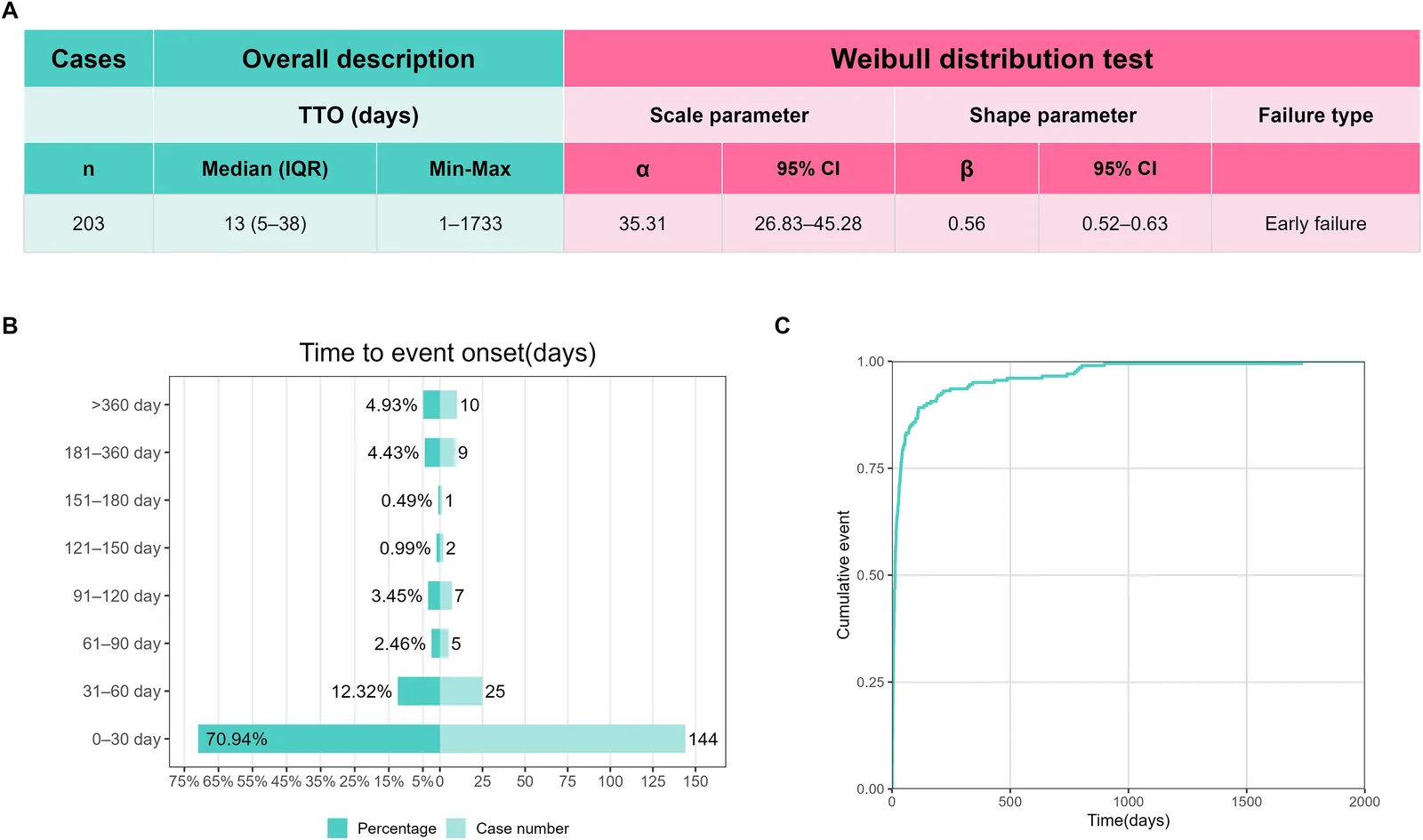
Time-to-onset analysis in the FAERS database. Panel A summarizes the number of evaluable reports, median time to onset, range, and Weibull parameters. Panel B shows the distribution of reported onset times by time interval. Panel C shows the cumulative distribution of reported onset times. Only FAERS reports with complete and valid onset-time data were included. Abbreviations: FAERS, FDA Adverse Event Reporting System; TTO, time to onset; IQR, interquartile range; CI, confidence interval.

Time-to-onset (TTO) analysis was performed using FAERS data, and the results are presented in Fig 6.

In the FAERS database, 203 of 520 eligible reports contained valid onset-time data and were included in the TTO analysis, corresponding to 39.0% of eligible reports. The remaining 317 reports were excluded because of missing or invalid date information. The median time to onset was 13 days (IQR, 5–38), with a range of 1–1733 days. As shown in Fig 6B, the onset-time distribution demonstrated marked early clustering: 70.94% of evaluable events (144/203) had onset dates 0–30 days, followed by 12.32% (25/203) within 31–60 days. Thereafter, the proportion declined to 2.46% (5/203) at 61–90 days, 3.45% (7/203) at 91–120 days, 0.99% (2/203) at 121–150 days, 0.49% (1/203) at 151–180 days, 4.43% (9/203) at 181–360 days, and 4.93% (10/203) beyond 360 days. Weibull analysis yielded a scale parameter (α) of 35.31 (95% CI 26.83–45.28) and a shape parameter (β) of 0.56 (95% CI 0.52–0.63), indicating an early-failure pattern. Consistently, the cumulative distribution curve in Fig 6C rose steeply during the initial treatment period and then gradually plateaued, suggesting that reported events were clustered during the early phase after treatment initiation.

## 4 Discussion

### 4.1 Study contribution

This study provides a complementary real-world pharmacovigilance assessment of entrectinib-associated adverse event reporting patterns in NSCLC. Rather than relying on a single spontaneous reporting system, we integrated two independent pharmacovigilance databases, FAERS and JADER, to evaluate whether signals could be reproduced across different reporting environments [28]. The main contribution of this study is therefore not the identification of entirely novel toxicity categories, because neurologic, cardiac, renal, and other adverse events have been reported previously, but the cross-database characterization of PT-level reporting signals, together with exploratory age- and sex-stratified subgroup analyses and a FAERS-based time-to-onset analysis in a postmarketing setting.

The cross-database concordance of neurologic, cardiac, and renal PT signals supports the presence of a reproducible reporting pattern in postmarketing data [6]. In addition, the subgroup analyses provided preliminary evidence of possible age- and sex-related reporting heterogeneity, while the time-to-onset analysis suggested early clustering of reported events after treatment initiation. These additional analyses extend the study beyond signal listing alone and may help generate more clinically oriented hypotheses regarding monitoring priorities during entrectinib treatment. However, these findings should be interpreted as signal-generating evidence rather than proof of causal drug toxicity [19]. Low-frequency PTs with high disproportionality estimates were retained to ensure transparency, but their clinical relevance remains uncertain because several were based on sparse counts and wide confidence intervals. Overall, this study aims to complement existing safety knowledge by identifying reproducible and exploratory reporting signals and by describing subgroup and temporal patterns that may help prioritize future clinical monitoring and validation studies.

### 4.2 Interpretation of the findings and possible mechanisms

This multi-source pharmacovigilance analysis provides a real-world characterization of entrectinib-associated adverse event reporting patterns in NSCLC using two independent spontaneous reporting systems. Overall, FAERS and JADER showed broadly concordant signal patterns at both the SOC and PT levels, particularly involving nervous system disorders, cardiac disorders, renal and urinary disorders, and investigation-related events. Because these findings were derived from spontaneous reporting databases, they should be interpreted as disproportional reporting signals rather than evidence of incidence, absolute risk, or confirmed causal adverse drug reactions.

The annual reporting trends should also be interpreted cautiously. The observed changes in the number of reports over time describe reporting activity in FAERS and JADER, but they do not necessarily reflect changes in the true incidence of entrectinib-associated adverse events. Increased reporting may be influenced by expanded drug utilization after approval, greater clinician familiarity with entrectinib, heightened awareness of specific adverse events, regulatory communications, differences in pharmacovigilance practice, or public and media attention. Therefore, the annual trend analysis is best understood as a descriptive overview of reporting patterns rather than as evidence of temporal changes in clinical risk.

From a biological perspective, several replicated signal clusters have plausible but non-confirmatory explanations. Entrectinib inhibits TRK, ROS1, and ALK and has intracranial antitumor activity, providing a biologically plausible context for the repeated reporting of neurologic events such as dizziness, cognitive disorder, ataxia, gait disturbance, syncope, and taste disorder [29,30]. However, the present study cannot determine whether these neurologic reports were caused by direct central nervous system pharmacologic effects, brain metastases, prior cranial irradiation, concomitant medications, or other patient-related factors [31]. Therefore, these findings should be interpreted as neurologic monitoring priorities rather than evidence of a specific neurologic injury pathway.

The replicated cardiac signals, including cardiac failure, cardiomyopathy, myocarditis, arrhythmia, electrocardiogram QT prolonged, troponin I increased, and left ventricular dysfunction, may also reflect multiple overlapping explanations. These may include kinase-inhibition-related effects on cardiomyocyte signaling, electrophysiologic vulnerability, myocardial stress in patients with advanced cancer, pre-existing cardiovascular disease, prior anticancer therapy, or concomitant QT-prolonging medications [32,33]. Because FAERS and JADER do not provide systematic information on baseline cardiac function, cardiac imaging, electrocardiographic monitoring, biomarkers, or myocarditis adjudication, these cardiac findings should be regarded as clinically relevant pharmacovigilance signals rather than mechanistically confirmed cardiotoxicity.

Similarly, renal impairment, renal disorder, blood creatinine increased, and hyperuricaemia may arise through multiple pathways, including direct or indirect renal effects, dehydration, hemodynamic instability, concomitant nephrotoxic drugs, tumor-related clinical deterioration, tumor lysis-related metabolic disturbance, or progression of baseline renal dysfunction [34]. The available spontaneous reporting data do not allow these possibilities to be separated. Therefore, the renal and metabolic signals should be interpreted as areas for clinical awareness and future validation rather than evidence of a single causal pathway.

Disease progression requires separate consideration. Although disease progression met the statistical signal criterion in FAERS, it is not a toxicity-specific adverse event in the same sense as dizziness, renal impairment, or cardiac failure. Instead, it may reflect treatment failure, tumor aggressiveness, acquired resistance, or the underlying disease course. For this reason, disease progression was retained in the signal-detection output for transparency but was interpreted as a disease-course-related reporting term rather than as part of the core toxicity-related signal profile of entrectinib. This distinction is important because indication-related clinical complexity may influence disproportionality estimates even when the analysis is restricted to an NSCLC reporting background.

A key finding of this study is the cross-database replication of PT-level signals. The 23 cross-database replicated PTs, which spanned neurologic, cardiac, renal, vascular, hepatic, metabolic, and laboratory-related domains, constituted the most reproducible component of the analysis and were therefore emphasized over database-specific or single-method signals. In contrast, 58 positive PTs were unique to FAERS and 8 were unique to JADER. This asymmetry may reflect differences in database size, reporting sources, regional prescribing patterns, clinical practice, regulatory context, coding habits, and reporting sensitivity. It may also reflect random reporting variation, especially for rare events. Therefore, database-specific PTs should be regarded as exploratory findings requiring further validation rather than as definitive database-specific toxicities.

Several low-frequency PTs showed large disproportionality estimates, including dyslalia, electrocardiogram T wave inversion, ligament rupture, joint effusion, urinary incontinence, and orthostatic hypotension in FAERS, as well as troponin I increased, left ventricular dysfunction, arrhythmia, and hyperuricaemia in JADER. These signals were retained in the supplementary tables to ensure transparency. However, because several were based on sparse counts and wide confidence intervals, they are statistically unstable and may be sensitive to coding differences, reporting sensitivity, random fluctuation, and multiplicity-related false-positive inflation. Accordingly, these low-frequency signals should be interpreted as exploratory pharmacovigilance signals rather than confirmed adverse drug reactions.

The subgroup analyses suggested possible reporting heterogeneity by age and sex, but these findings should be interpreted cautiously. In FAERS, several renal and mobility-related events were relatively more represented among older patients, whereas selected progression-related, hepatic, neurologic, or biochemical events were relatively enriched in younger patients. Sex-stratified analyses suggested that ataxia was more frequently reported in females, whereas blood creatinine increased and pleural effusion were relatively more frequent in males. Similar directional patterns were observed for selected PTs in JADER, but several estimates were imprecise because of small cell counts and wide confidence intervals. In addition, age categories differed between FAERS and JADER because of differences in publicly available data structures. Therefore, the subgroup findings should be viewed as hypothesis-generating observations rather than definitive evidence of age- or sex-specific risk differences.

The FAERS-based time-to-onset analysis suggested early clustering of reported events, with a median onset of 13 days and more than 70% of evaluable reports having an onset date within the first 30 days after treatment initiation. The Weibull shape parameter was consistent with an early-failure reporting pattern [27]. These findings support closer clinical attention during the early treatment period, particularly for neurologic symptoms, renal laboratory abnormalities, hemodynamic changes, and cardiac findings. However, the TTO analysis included only 203 of 520 eligible FAERS reports with valid onset-time data, and a reliable JADER-based TTO analysis could not be performed because of insufficient date-paired reports. Therefore, the temporal pattern should be interpreted cautiously because missing onset dates may have introduced selection bias.

Taken together, the present findings suggest that entrectinib-associated adverse event reporting in NSCLC is characterized by a reproducible core of neurologic, cardiac, renal, and laboratory-related signals across FAERS and JADER, together with additional database-specific and low-frequency exploratory signals. The clinical value of these findings lies in prioritizing areas for awareness and monitoring rather than establishing causality. Future cohort-based studies, registry analyses, and mechanistic investigations with detailed clinical covariates will be needed to determine which of these pharmacovigilance signals represent true drug-related toxicities and how they should be incorporated into risk-adapted monitoring strategies [35].

### 4.3 Limitations

Several limitations should be considered when interpreting these findings. First, FAERS and JADER are spontaneous reporting systems and are therefore subject to underreporting, stimulated reporting, incomplete documentation, heterogeneous report quality, delayed submissions, and reporting bias [36]. The annual reporting trends observed in this study should be interpreted as changes in reporting activity rather than changes in the true incidence of entrectinib-associated adverse events, because report counts may be influenced by drug utilization, clinician awareness, regulatory communications, media attention, and differences in pharmacovigilance practice. In addition, spontaneous reporting databases lack reliable exposure denominators and therefore cannot be used to estimate incidence rates, absolute risks, or comparative safety profiles relative to alternative therapies [37].

Second, although the disproportionality analyses were conducted within an NSCLC reporting background rather than against the entire spontaneous reporting database, residual confounding could not be fully excluded. ROS1 fusion status could not be verified in FAERS or JADER, and the NSCLC reference population could not be considered molecularly comparable to the entrectinib group. Consequently, differences in molecular subtype and treatment indication may have contributed to the observed reporting patterns. Patients with NSCLC may have advanced disease, comorbidities, prior anticancer therapy, concomitant medications, variable treatment duration, dose modifications, and competing causes of organ dysfunction. Therefore, some reported cardiac, renal, neurologic, or laboratory-related events may reflect the underlying disease, concomitant treatment, or patient-related factors rather than isolated effects of entrectinib [38]. The available FAERS and JADER data did not provide sufficient clinical granularity to fully adjust for these factors or to quantify the impact of concomitant medications. Similarly, disease progression was retained in the signal-detection output for transparency, but it was interpreted as a disease-course-related reporting term rather than a toxicity-specific adverse event signal.

Third, the analysis involved a large number of PT-level comparisons across multiple disproportionality methods, and no formal multiplicity adjustment was applied [39]. Because a single patient or case report could contribute multiple PT events, event-level observations were not fully independent, and within-report clustering may have influenced the resulting disproportionality estimates. Although cross-database replicated PTs were emphasized as the most reproducible findings, signals detected in only one database, by only one method, or based on sparse counts should be interpreted cautiously. Several low-frequency PTs showed very large ROR estimates with wide confidence intervals, indicating statistical instability and possible sensitivity to random fluctuation, coding differences, reporting sensitivity, and false-positive signal inflation. Subgroup analyses were also exploratory because several age- and sex-stratified estimates were based on small numbers of reports, and age categories differed between FAERS and JADER. Finally, the TTO analysis was limited to FAERS and included only 203 of 520 eligible reports with complete and valid onset-time data; therefore, missing onset dates may have introduced selection bias [36]. These findings should be regarded as hypothesis-generating pharmacovigilance signals and require further validation in prospective cohorts, registry-based studies, electronic health record datasets, or mechanistic investigations with detailed patient-level information [40].

## 5 Conclusion

In this dual-database pharmacovigilance study, entrectinib-associated adverse event reports in patients with NSCLC showed a reproducible cross-database signal pattern. By integrating FAERS and JADER, we identified a consistent cluster of disproportional reporting signals involving nervous system disorders, cardiac disorders, renal and urinary disorders, and investigation-related abnormalities. At the PT level, 23 signals were shared across both databases, representing the most stable and clinically informative component of the analysis. These replicated signals suggest that neurologic events, cardio-renal abnormalities, and selected laboratory findings should be regarded as key areas of safety awareness during entrectinib treatment.

Beyond confirming the value of cross-database replication, this study adds several layers of clinical information. The FAERS-based time-to-onset analysis suggested that many reported events occurred early after treatment initiation, supporting closer attention during the initial treatment period. Subgroup analyses further indicated possible age- and sex-related reporting heterogeneity, such as relatively more renal and mobility-related reports in older patients and more frequent reporting of ataxia in females. Although these subgroup findings should not be interpreted as definitive demographic risk differences, they may help generate clinically relevant hypotheses for future risk-stratified monitoring.

Overall, the present findings extend existing knowledge of entrectinib safety by distinguishing reproducible reporting signals from database-specific and low-frequency exploratory findings. Rather than simply expanding the list of possible adverse events, this study highlights the importance of structured monitoring for neurologic symptoms, renal function, hemodynamic changes, and cardiac abnormalities, particularly during the early phase of treatment. Future cohort-based studies, registry analyses, and mechanistic investigations with detailed patient-level information are warranted to validate these pharmacovigilance signals and refine risk-adapted safety monitoring strategies for patients receiving entrectinib.

## Supporting information

S1 Table Event-level two-by-two contingency table for PT-level disproportionality analysis. (DOCX)

S2 Table Disproportionality analysis algorithms and signal criteria. (DOCX)

S3 Table SOC-level disproportionality analysis of entrectinib-associated adverse events in FAERS and JADER. (DOCX)

S4 Table Positive PT signals of entrectinib-associated adverse events in FAERS. (DOCX)

S5 Table Positive PT signals of entrectinib-associated adverse events in JADER. (DOCX)

S6 Table Age-stratified subgroup analysis of positive PT signals in the FAERS database. (DOCX)

S7 Table Sex-stratified subgroup analysis of positive PT signals in the FAERS database. (DOCX)

S8 Table Sex-stratified subgroup analysis of positive PT signals in the JADER database. (DOCX)

S1 Dataset FAERS de-duplicated event-level analytic dataset. (XLSX)

S2 Dataset JADER de-duplicated event-level analytic dataset. (XLSX)
