# Supplementary material for: Safety profile of entrectinib in NSCLC: Multi-source pharmacovigilance analysis using FAERS and JADER
Source: PLoS One. 2026 Sep 15;21(9):e0358331. doi: 10.1371/journal.pone.0358331 (PMC13577382; doi:10.1371/journal.pone.0358331)
Supplement: Supplementary file 1 [file pone.0358331.s001.docx]

S1 Table. Event-level two-by-two contingency table for PT-level disproportionality analysis

| Database | **PT** | **a** | **b** | **c** | **d** |
| --- | --- | --- | --- | --- | --- |
| FAERS | Dizziness | 59 | 1514 | 648 | 215187 |
| FAERS | Taste disorder | 45 | 1528 | 167 | 215668 |
| FAERS | Renal impairment | 45 | 1528 | 918 | 214917 |
| FAERS | Disease progression | 42 | 1531 | 1754 | 214081 |
| FAERS | Blood creatinine increased | 41 | 1532 | 693 | 215142 |
| FAERS | Cardiac failure | 30 | 1543 | 553 | 215282 |
| FAERS | Cognitive disorder | 25 | 1548 | 193 | 215642 |
| FAERS | Weight increased | 20 | 1553 | 426 | 215409 |
| FAERS | Ataxia | 19 | 1554 | 51 | 215784 |
| FAERS | Pleural effusion | 19 | 1554 | 1316 | 214519 |
| FAERS | Gait disturbance | 16 | 1557 | 365 | 215470 |
| FAERS | Oedema | 15 | 1558 | 503 | 215332 |
| FAERS | Syncope | 15 | 1558 | 234 | 215601 |
| FAERS | Renal disorder | 15 | 1558 | 232 | 215603 |
| FAERS | Constipation | 15 | 1558 | 1090 | 214745 |
| FAERS | Hypotension | 15 | 1558 | 513 | 215322 |
| FAERS | Hepatic function abnormal | 15 | 1558 | 1061 | 214774 |
| FAERS | Metastases to central nervous system | 15 | 1558 | 1160 | 214675 |
| FAERS | Myocarditis | 12 | 1561 | 477 | 215358 |
| FAERS | Cardiomyopathy | 12 | 1561 | 125 | 215710 |
| FAERS | Pericardial effusion | 12 | 1561 | 528 | 215307 |
| FAERS | Ejection fraction decreased | 12 | 1561 | 136 | 215699 |
| FAERS | Arthralgia | 11 | 1562 | 728 | 215107 |
| FAERS | Fall | 10 | 1563 | 469 | 215366 |
| FAERS | Cardiac disorder | 10 | 1563 | 133 | 215702 |
| FAERS | Seizure | 9 | 1564 | 303 | 215532 |
| FAERS | Dysgeusia | 9 | 1564 | 144 | 215691 |
| FAERS | Muscular weakness | 9 | 1564 | 398 | 215437 |
| FAERS | Dysphagia | 8 | 1565 | 536 | 215299 |
| FAERS | Visual impairment | 8 | 1565 | 220 | 215615 |
| FAERS | Urinary retention | 8 | 1565 | 84 | 215751 |
| FAERS | Memory impairment | 7 | 1566 | 202 | 215633 |
| FAERS | Blood creatine phosphokinase increased | 7 | 1566 | 451 | 215384 |
| FAERS | Hypoaesthesia | 6 | 1567 | 296 | 215539 |
| FAERS | Dysarthria | 6 | 1567 | 109 | 215726 |
| FAERS | Paraesthesia | 6 | 1567 | 261 | 215574 |
| FAERS | Balance disorder | 6 | 1567 | 147 | 215688 |
| FAERS | Orthostatic hypotension | 6 | 1567 | 52 | 215783 |
| FAERS | Ventricular tachycardia | 6 | 1567 | 80 | 215755 |
| FAERS | Cardiac failure congestive | 6 | 1567 | 136 | 215699 |
| FAERS | Electrocardiogram qt prolonged | 6 | 1567 | 206 | 215629 |
| FAERS | Amnesia | 5 | 1568 | 73 | 215762 |
| FAERS | Shock | 5 | 1568 | 77 | 215758 |
| FAERS | Vision blurred | 5 | 1568 | 158 | 215677 |
| FAERS | Pain of skin | 5 | 1568 | 32 | 215803 |
| FAERS | Arrhythmia | 5 | 1568 | 143 | 215692 |
| FAERS | Urinary incontinence | 5 | 1568 | 49 | 215786 |
| FAERS | Aphasia | 4 | 1569 | 97 | 215738 |
| FAERS | Dyslalia | 4 | 1569 | 6 | 215829 |
| FAERS | Feeling abnormal | 4 | 1569 | 118 | 215717 |
| FAERS | Dry mouth | 4 | 1569 | 197 | 215638 |
| FAERS | Humerus fracture | 4 | 1569 | 14 | 215821 |
| FAERS | Neuralgia | 4 | 1569 | 60 | 215775 |
| FAERS | Ill-defined disorder | 4 | 1569 | 106 | 215729 |
| FAERS | Troponin i increased | 4 | 1569 | 19 | 215816 |
| FAERS | Hypoaesthesia oral | 4 | 1569 | 17 | 215818 |
| FAERS | Cerebral haemorrhage | 4 | 1569 | 131 | 215704 |
| FAERS | Cardiac failure acute | 4 | 1569 | 83 | 215752 |
| FAERS | Left ventricular dysfunction | 4 | 1569 | 82 | 215753 |
| FAERS | Fracture | 3 | 1570 | 115 | 215720 |
| FAERS | Wheezing | 3 | 1570 | 72 | 215763 |
| FAERS | Joint effusion | 3 | 1570 | 10 | 215825 |
| FAERS | Ligament rupture | 3 | 1570 | 2 | 215833 |
| FAERS | Cardiotoxicity | 3 | 1570 | 108 | 215727 |
| FAERS | Contusion | 3 | 1570 | 49 | 215786 |
| FAERS | Neurotoxicity | 3 | 1570 | 112 | 215723 |
| FAERS | Hyperuricaemia | 3 | 1570 | 56 | 215779 |
| FAERS | Dyskinesia | 3 | 1570 | 13 | 215822 |
| FAERS | Mental disorder | 3 | 1570 | 62 | 215773 |
| FAERS | Hyperaesthesia | 3 | 1570 | 29 | 215806 |
| FAERS | Palpitations | 3 | 1570 | 98 | 215737 |
| FAERS | Incontinence | 3 | 1570 | 16 | 215819 |
| FAERS | Fluid retention | 3 | 1570 | 86 | 215749 |
| FAERS | Movement disorder | 3 | 1570 | 82 | 215753 |
| FAERS | Ventricular arrhythmia | 3 | 1570 | 13 | 215822 |
| FAERS | Generalised oedema | 3 | 1570 | 118 | 215717 |
| FAERS | Pulmonary hypertension | 3 | 1570 | 54 | 215781 |
| FAERS | Blood pressure decreased | 3 | 1570 | 125 | 215710 |
| FAERS | Central nervous system lesion | 3 | 1570 | 97 | 215738 |
| FAERS | Electrocardiogram t wave inversion | 3 | 1570 | 5 | 215830 |
| FAERS | Electrocardiogram abnormal | 3 | 1570 | 17 | 215818 |
| JADER | Renal impairment | 29 | 364 | 374 | 38388 |
| JADER | Cardiac failure | 17 | 376 | 196 | 38566 |
| JADER | Renal disorder | 16 | 377 | 76 | 38686 |
| JADER | Cognitive disorder | 16 | 377 | 21 | 38741 |
| JADER | Syncope | 14 | 379 | 16 | 38746 |
| JADER | Blood creatinine increased | 13 | 380 | 53 | 38709 |
| JADER | Neutrophil count decreased | 12 | 381 | 557 | 38205 |
| JADER | Electrocardiogram QT prolonged | 9 | 384 | 169 | 38593 |
| JADER | Hepatic function abnormal | 9 | 384 | 424 | 38338 |
| JADER | Myocarditis | 9 | 384 | 153 | 38609 |
| JADER | Anaemia | 9 | 384 | 237 | 38525 |
| JADER | Neutropenia | 8 | 385 | 316 | 38446 |
| JADER | Ataxia | 7 | 386 | 2 | 38760 |
| JADER | Dizziness | 7 | 386 | 49 | 38713 |
| JADER | Cardiac disorder | 7 | 386 | 4 | 38758 |
| JADER | Delirium | 5 | 388 | 57 | 38705 |
| JADER | Cardiomyopathy | 5 | 388 | 37 | 38725 |
| JADER | Nausea | 5 | 388 | 165 | 38597 |
| JADER | Troponin I increased | 4 | 389 | 5 | 38757 |
| JADER | Gait disturbance | 4 | 389 | 18 | 38744 |
| JADER | Vomiting | 4 | 389 | 61 | 38701 |
| JADER | Ejection fraction decreased | 4 | 389 | 23 | 38739 |
| JADER | Taste disorder | 4 | 389 | 30 | 38732 |
| JADER | Shock | 3 | 390 | 26 | 38736 |
| JADER | Hyperuricaemia | 3 | 390 | 5 | 38757 |
| JADER | Oedema peripheral | 3 | 390 | 37 | 38725 |
| JADER | Arrhythmia | 3 | 390 | 25 | 38737 |
| JADER | Cardiac failure acute | 3 | 390 | 31 | 38731 |
| JADER | Hypotension | 3 | 390 | 27 | 38735 |
| JADER | Left ventricular dysfunction | 3 | 390 | 3 | 38759 |
| JADER | Pulmonary embolism | 3 | 390 | 81 | 38681 |

Note: For each preferred term (PT), a two-by-two contingency table was constructed separately in FAERS and JADER. In this event-level disproportionality analysis, “a” represents the number of target PT events in the entrectinib-NSCLC target population, “b” represents all other PT events in the target population, “c” represents the number of target PT events in the NSCLC reference population after excluding entrectinib-related reports, and “d” represents all other PT events in the reference population. Target AEs refer to events coded with the PT of interest, whereas other AEs refer to all remaining PT events. The total number of PT events used for each PT-specific contingency table is calculated as a + b + c + d.
