# Supplementary material for: Safety profile of entrectinib in NSCLC: Multi-source pharmacovigilance analysis using FAERS and JADER
Source: PLoS One. 2026 Sep 15;21(9):e0358331. doi: 10.1371/journal.pone.0358331 (PMC13577382; doi:10.1371/journal.pone.0358331)
Supplement: Supplementary file 2 [file pone.0358331.s002.docx]

S2 Table. Disproportionality analysis algorithms and signal criteria

| **Methods** | **Formulas** | **Criteria** |
| --- | --- | --- |
| ROR | $ROR=\frac{ad}{bc}$ | *lower limit of 95% CI > 1, N ≥ 3* |
|  | $95\% CI=exp[\ln(ROR)\pm1.96\sqrt{\frac{1}{a}+\frac{1}{b}+\frac{1}{c}+\frac{1}{d}}]$ |  |
| PRR | $PRR=\frac{a/(a+b)}{c/(c+d)}$ | *PRR≥2,* $\chi^{2}$*≥ 4, N ≥ 3* |
|  | $\chi^{2}=\frac{(ad-bc)^{2}(a+b+c+d)}{(a+b)(c+d)(a+c)(b+d)}$ |  |
| BCPNN | $IC=\log_{2} [\frac{a(a+b+c+d)}{(a+c)(a+b)}]$ | *IC025 > 0* |
|  | $95\%CI=E(IC)\pm2\sqrt{V(IC)}$ |  |
| MGPS | $EBGM=\frac{a(a+b+c+d)}{(a+c)(a+b)}$ | *EBGM05 > 2* |
|  | $95\%CI=exp[\ln(EBGM)\pm1.96\sqrt{\frac{1}{a}+\frac{1}{b}+\frac{1}{c}+\frac{1}{d}}]$ |  |

Note: ROR, reporting odds ratio; PRR, proportional reporting ratio; BCPNN, Bayesian confidence propagation neural network; MGPS, multi-item gamma Poisson shrinker; CI, confidence interval; χ², chi-square statistic; IC025, lower limit of the 95% credibility interval for the information component; EBGM05, lower fifth percentile of the empirical Bayes geometric mean.
