# Supplementary material for: Safety profile of entrectinib in NSCLC: Multi-source pharmacovigilance analysis using FAERS and JADER
Source: PLoS One. 2026 Sep 15;21(9):e0358331. doi: 10.1371/journal.pone.0358331 (PMC13577382; doi:10.1371/journal.pone.0358331)
Supplement: Supplementary file 3 [file pone.0358331.s003.docx]

S3 Table. SOC-level disproportionality analysis of entrectinib-associated adverse events in FAERS and JADER

| **SOC** | **N** | **ROR**  **(95%CI)** | **PRR**  **(χ2)** | **EBGM**  **(EBGM05)** | **IC**  **(IC025)** |
| --- | --- | --- | --- | --- | --- |
| **FAERS** |  |  |  |  |  |
| Nervous system disorders | 312 | 4.48 ( 3.95 - 5.08 ) | 3.79 ( 659.38 ) | 3.72 ( 3.28 ) | 1.89 ( 1.7 ) |
| General disorders and administration site conditions | 208 | 0.92 ( 0.8 - 1.07 ) | 0.93 ( 1.19 ) | 0.93 ( 0.81 ) | -0.1 ( -0.31 ) |
| Investigations | 161 | 1.39 ( 1.18 - 1.64 ) | 1.35 ( 15.73 ) | 1.35 ( 1.14 ) | 0.43 ( 0.19 ) |
| Cardiac disorders | 148 | 3.2 ( 2.7 - 3.8 ) | 2.99 ( 198.55 ) | 2.95 ( 2.49 ) | 1.56 ( 1.29 ) |
| Gastrointestinal disorders | 115 | 0.75 ( 0.62 - 0.91 ) | 0.77 ( 8.52 ) | 0.77 ( 0.64 ) | -0.37 ( -0.65 ) |
| Respiratory, thoracic and mediastinal disorders | 107 | 0.75 ( 0.62 - 0.91 ) | 0.77 ( 8.2 ) | 0.77 ( 0.63 ) | -0.38 ( -0.66 ) |
| Renal and urinary disorders | 96 | 2.04 ( 1.66 - 2.52 ) | 1.98 ( 47.35 ) | 1.97 ( 1.6 ) | 0.98 ( 0.66 ) |
| Musculoskeletal and connective tissue disorders | 58 | 1.29 ( 0.99 - 1.67 ) | 1.28 ( 3.54 ) | 1.27 ( 0.98 ) | 0.35 ( -0.04 ) |
| Injury, poisoning and procedural complications | 57 | 0.77 ( 0.59 - 1.01 ) | 0.78 ( 3.63 ) | 0.78 ( 0.6 ) | -0.35 ( -0.74 ) |
| Neoplasms benign, malignant and unspecified (incl cysts and polyps) | 42 | 0.31 ( 0.23 - 0.42 ) | 0.33 ( 63.63 ) | 0.33 ( 0.24 ) | -1.61 ( -2.04 ) |
| Infections and infestations | 40 | 0.42 ( 0.3 - 0.57 ) | 0.43 ( 31.93 ) | 0.43 ( 0.32 ) | -1.21 ( -1.65 ) |
| Vascular disorders | 39 | 1.3 ( 0.95 - 1.79 ) | 1.3 ( 2.65 ) | 1.29 ( 0.94 ) | 0.37 ( -0.11 ) |
| Psychiatric disorders | 38 | 1.87 ( 1.35 - 2.58 ) | 1.85 ( 14.74 ) | 1.84 ( 1.33 ) | 0.88 ( 0.37 ) |
| Skin and subcutaneous tissue disorders | 32 | 0.34 ( 0.24 - 0.49 ) | 0.36 ( 39.61 ) | 0.36 ( 0.25 ) | -1.49 ( -1.97 ) |
| Hepatobiliary disorders | 27 | 0.49 ( 0.33 - 0.71 ) | 0.49 ( 14.38 ) | 0.5 ( 0.34 ) | -1.01 ( -1.54 ) |
| Metabolism and nutrition disorders | 26 | 0.46 ( 0.32 - 0.68 ) | 0.47 ( 15.73 ) | 0.48 ( 0.32 ) | -1.07 ( -1.61 ) |
| Eye disorders | 23 | 1.55 ( 1.03 - 2.35 ) | 1.54 ( 4.41 ) | 1.54 ( 1.02 ) | 0.62 ( -0.01 ) |
| Blood and lymphatic system disorders | 19 | 0.2 ( 0.13 - 0.32 ) | 0.21 ( 59.29 ) | 0.21 ( 0.14 ) | -2.24 ( -2.83 ) |
| Surgical and medical procedures | 8 | 1.77 ( 0.88 - 3.56 ) | 1.76 ( 2.63 ) | 1.76 ( 0.87 ) | 0.81 ( -0.28 ) |
| Endocrine disorders | 7 | 0.23 ( 0.11 - 0.47 ) | 0.23 ( 18.48 ) | 0.23 ( 0.11 ) | -2.12 ( -3 ) |
| Ear and labyrinth disorders | 4 | 1 ( 0.37 - 2.67 ) | 1 ( 0 ) | 1 ( 0.37 ) | 0 ( -1.3 ) |
| Congenital, familial and genetic disorders | 3 | 0.41 ( 0.13 - 1.27 ) | 0.41 ( 2.55 ) | 0.41 ( 0.13 ) | -1.28 ( -2.5 ) |
| Reproductive system and breast disorders | 2 | 0.82 ( 0.21 - 3.31 ) | 0.82 ( 0.07 ) | 0.83 ( 0.21 ) | -0.28 ( -1.87 ) |
| Immune system disorders | 1 | 0.06 ( 0.01 - 0.46 ) | 0.06 ( 13.61 ) | 0.07 ( 0.01 ) | -3.94 ( -5.07 ) |
| JADER |  |  |  |  |  |
| Nervous system disorders | 64 | 4.76 ( 3.63 - 6.26 ) | 4.15 ( 153.05 ) | 4.02 ( 3.06 ) | 2.01 ( 0.33 ) |
| Cardiac disorders | 63 | 5.43 ( 4.12 - 7.15 ) | 4.72 ( 182.6 ) | 4.55 ( 3.45 ) | 2.19 ( 0.51 ) |
| Investigations | 58 | 2.44 ( 1.84 - 3.24 ) | 2.23 ( 41.17 ) | 2.2 ( 1.66 ) | 1.14 ( -0.53 ) |
| Renal and urinary disorders | 48 | 3.92 ( 2.88 - 5.33 ) | 3.56 ( 88.49 ) | 3.47 ( 2.56 ) | 1.8 ( 0.12 ) |
| Respiratory, thoracic and mediastinal disorders | 30 | 0.35 ( 0.24 - 0.51 ) | 0.4 ( 33.03 ) | 0.4 ( 0.28 ) | -1.31 ( -2.98 ) |
| Gastrointestinal disorders | 19 | 0.58 ( 0.37 - 0.92 ) | 0.6 ( 5.42 ) | 0.6 ( 0.38 ) | -0.73 ( -2.4 ) |
| Blood and lymphatic system disorders | 18 | 0.89 ( 0.55 - 1.43 ) | 0.9 ( 0.22 ) | 0.9 ( 0.56 ) | -0.16 ( -1.83 ) |
| General disorders and administration site conditions | 17 | 0.79 ( 0.48 - 1.29 ) | 0.8 ( 0.91 ) | 0.8 ( 0.49 ) | -0.32 ( -1.99 ) |
| Hepatobiliary disorders | 13 | 0.62 ( 0.36 - 1.08 ) | 0.63 ( 2.9 ) | 0.64 ( 0.36 ) | -0.65 ( -2.33 ) |
| Skin and subcutaneous tissue disorders | 12 | 0.46 ( 0.26 - 0.81 ) | 0.47 ( 7.43 ) | 0.48 ( 0.27 ) | -1.07 ( -2.74 ) |
| Psychiatric disorders | 9 | 4.86 ( 2.47 - 9.56 ) | 4.77 ( 25.73 ) | 4.6 ( 2.34 ) | 2.2 ( 0.52 ) |
| Injury, poisoning and procedural complications | 9 | 1.43 ( 0.73 - 2.78 ) | 1.42 ( 1.11 ) | 1.41 ( 0.73 ) | 0.5 ( -1.18 ) |
| Vascular disorders | 8 | 1.61 ( 0.79 - 3.25 ) | 1.59 ( 1.77 ) | 1.58 ( 0.78 ) | 0.66 ( -1.01 ) |
| Metabolism and nutrition disorders | 7 | 0.44 ( 0.21 - 0.94 ) | 0.45 ( 4.74 ) | 0.46 ( 0.22 ) | -1.13 ( -2.8 ) |
| Infections and infestations | 7 | 0.3 ( 0.14 - 0.62 ) | 0.31 ( 11.5 ) | 0.31 ( 0.15 ) | -1.69 ( -3.36 ) |
| Endocrine disorders | 3 | 0.08 ( 0.03 - 0.25 ) | 0.09 ( 31.74 ) | 0.09 ( 0.03 ) | -3.52 ( -5.19 ) |
| Surgical and medical procedures | 3 | 8.05 ( 2.47 - 26.22 ) | 8 ( 17.01 ) | 7.47 ( 2.29 ) | 2.9 ( 1.17 ) |
| Musculoskeletal and connective tissue disorders | 2 | 0.23 ( 0.06 - 0.91 ) | 0.23 ( 5.28 ) | 0.23 ( 0.06 ) | -2.11 ( -3.79 ) |
| Eye disorders | 1 | 0.48 ( 0.07 - 3.43 ) | 0.48 ( 0.56 ) | 0.48 ( 0.07 ) | -1.05 ( -2.73 ) |
| Neoplasms benign, malignant and unspecified (incl cysts and polyps) | 1 | 0.04 ( 0.01 - 0.28 ) | 0.04 ( 23.08 ) | 0.04 ( 0.01 ) | -4.55 ( -6.22 ) |
| Congenital, familial and genetic disorders | 1 | 0.83 ( 0.12 - 5.94 ) | 0.83 ( 0.04 ) | 0.83 ( 0.12 ) | -0.27 ( -1.96 ) |

Note: SOC, system organ class; ROR, reporting odds ratio; PRR, proportional reporting ratio; EBGM, empirical Bayes geometric mean; EBGM05, lower fifth percentile of EBGM; IC, information component; IC025, lower limit of the 95% credibility interval; CI, confidence interval; χ², chi-square statistic.
