# Supplementary material for: Safety profile of entrectinib in NSCLC: Multi-source pharmacovigilance analysis using FAERS and JADER
Source: PLoS One. 2026 Sep 15;21(9):e0358331. doi: 10.1371/journal.pone.0358331 (PMC13577382; doi:10.1371/journal.pone.0358331)
Supplement: Supplementary file 4 [file pone.0358331.s004.docx]

S4 Table. Positive PT signals of entrectinib-associated adverse events in FAERS

| **System Organ Class** | **PT** | **a** | **ROR(95%CI)** | **PRR(χ2)** | **EBGM(EBGM05)** | **IC(IC025)** |
| --- | --- | --- | --- | --- | --- | --- |
| Nervous System Disorders | Dizziness | 59 | 12.94 ( 9.87 - 16.97 ) | 12.49 ( 573.62 ) | 11.53 ( 8.79 ) | 3.53 ( 2.9 ) |
| Nervous System Disorders | Taste Disorder | 45 | 38.03 ( 27.26 - 53.06 ) | 36.97 ( 1241.91 ) | 29.34 ( 21.03 ) | 4.87 ( 3.7 ) |
| Renal And Urinary Disorders | Renal Impairment | 45 | 6.89 ( 5.09 - 9.34 ) | 6.73 ( 210.04 ) | 6.46 ( 4.77 ) | 2.69 ( 2.09 ) |
| General Disorders And Administration Site Conditions | Disease Progression | 42 | 3.35 ( 2.46 - 4.57 ) | 3.29 ( 65.76 ) | 3.23 ( 2.37 ) | 1.69 ( 1.17 ) |
| Investigations | Blood Creatinine Increased | 41 | 8.31 ( 6.04 - 11.43 ) | 8.12 ( 242.41 ) | 7.72 ( 5.61 ) | 2.95 ( 2.27 ) |
| Cardiac Disorders | Cardiac Failure | 30 | 7.57 ( 5.22 - 10.97 ) | 7.44 ( 159.16 ) | 7.11 ( 4.91 ) | 2.83 ( 2.03 ) |
| Nervous System Disorders | Cognitive Disorder | 25 | 18.04 ( 11.86 - 27.45 ) | 17.77 ( 350.71 ) | 15.85 ( 10.42 ) | 3.99 ( 2.73 ) |
| Investigations | Weight Increased | 20 | 6.51 ( 4.15 - 10.23 ) | 6.44 ( 88 ) | 6.2 ( 3.95 ) | 2.63 ( 1.66 ) |
| Nervous System Disorders | Ataxia | 19 | 51.73 ( 30.47 - 87.81 ) | 51.12 ( 680.43 ) | 37.51 ( 22.1 ) | 5.23 ( 2.99 ) |
| Respiratory, Thoracic And Mediastinal Disorders | Pleural Effusion | 19 | 1.99 ( 1.26 - 3.14 ) | 1.98 ( 9.16 ) | 1.97 ( 1.25 ) | 0.98 ( 0.25 ) |
| General Disorders And Administration Site Conditions | Gait Disturbance | 16 | 6.07 ( 3.67 - 10.03 ) | 6.01 ( 64.2 ) | 5.8 ( 3.51 ) | 2.54 ( 1.46 ) |
| General Disorders And Administration Site Conditions | Oedema | 15 | 4.12 ( 2.46 - 6.9 ) | 4.09 ( 34.11 ) | 4 ( 2.39 ) | 2 ( 1.01 ) |
| Nervous System Disorders | Syncope | 15 | 8.87 ( 5.25 - 14.99 ) | 8.8 ( 97.51 ) | 8.33 ( 4.93 ) | 3.06 ( 1.76 ) |
| Renal And Urinary Disorders | Renal Disorder | 15 | 8.95 ( 5.3 - 15.12 ) | 8.87 ( 98.51 ) | 8.39 ( 4.97 ) | 3.07 ( 1.77 ) |
| Gastrointestinal Disorders | Constipation | 15 | 1.9 ( 1.14 - 3.16 ) | 1.89 ( 6.21 ) | 1.88 ( 1.12 ) | 0.91 ( 0.1 ) |
| Vascular Disorders | Hypotension | 15 | 4.04 ( 2.41 - 6.77 ) | 4.01 ( 33.04 ) | 3.93 ( 2.34 ) | 1.97 ( 0.99 ) |
| Hepatobiliary Disorders | Hepatic Function Abnormal | 15 | 1.95 ( 1.17 - 3.25 ) | 1.94 ( 6.77 ) | 1.93 ( 1.15 ) | 0.95 ( 0.13 ) |
| Neoplasms Benign, Malignant And Unspecified (Incl Cysts And Polyps) | Metastases To Central Nervous System | 15 | 1.78 ( 1.07 - 2.97 ) | 1.77 ( 5.03 ) | 1.76 ( 1.06 ) | 0.82 ( 0.02 ) |
| Cardiac Disorders | Myocarditis | 12 | 3.47 ( 1.95 - 6.17 ) | 3.45 ( 20.43 ) | 3.39 ( 1.91 ) | 1.76 ( 0.7 ) |
| Cardiac Disorders | Cardiomyopathy | 12 | 13.27 ( 7.32 - 24.04 ) | 13.17 ( 123.23 ) | 12.11 ( 6.68 ) | 3.6 ( 1.86 ) |
| Cardiac Disorders | Pericardial Effusion | 12 | 3.13 ( 1.77 - 5.57 ) | 3.12 ( 16.93 ) | 3.07 ( 1.73 ) | 1.62 ( 0.59 ) |
| Investigations | Ejection Fraction Decreased | 12 | 12.19 ( 6.74 - 22.05 ) | 12.11 ( 112.44 ) | 11.21 ( 6.2 ) | 3.49 ( 1.81 ) |
| Musculoskeletal And Connective Tissue Disorders | Arthralgia | 11 | 2.08 ( 1.14 - 3.78 ) | 2.07 ( 6.04 ) | 2.06 ( 1.13 ) | 1.04 ( 0.07 ) |
| Injury, Poisoning And Procedural Complications | Fall | 10 | 2.94 ( 1.57 - 5.51 ) | 2.93 ( 12.44 ) | 2.89 ( 1.54 ) | 1.53 ( 0.41 ) |
| Cardiac Disorders | Cardiac Disorder | 10 | 10.38 ( 5.45 - 19.77 ) | 10.32 ( 78.3 ) | 9.67 ( 5.07 ) | 3.27 ( 1.52 ) |
| Nervous System Disorders | Seizure | 9 | 4.09 ( 2.11 - 7.96 ) | 4.08 ( 20.32 ) | 3.99 ( 2.05 ) | 2 ( 0.68 ) |
| Nervous System Disorders | Dysgeusia | 9 | 8.62 ( 4.39 - 16.93 ) | 8.58 ( 56.73 ) | 8.13 ( 4.14 ) | 3.02 ( 1.3 ) |
| Musculoskeletal And Connective Tissue Disorders | Muscular Weakness | 9 | 3.11 ( 1.61 - 6.04 ) | 3.1 ( 12.57 ) | 3.06 ( 1.58 ) | 1.61 ( 0.41 ) |
| Gastrointestinal Disorders | Dysphagia | 8 | 2.05 ( 1.02 - 4.13 ) | 2.05 ( 4.24 ) | 2.03 ( 1.01 ) | 1.02 ( -0.11 ) |
| Eye Disorders | Visual Impairment | 8 | 5.01 ( 2.47 - 10.16 ) | 4.99 ( 24.65 ) | 4.85 ( 2.39 ) | 2.28 ( 0.78 ) |
| Renal And Urinary Disorders | Urinary Retention | 8 | 13.13 ( 6.35 - 27.16 ) | 13.07 ( 81.44 ) | 12.02 ( 5.81 ) | 3.59 ( 1.42 ) |
| Nervous System Disorders | Memory Impairment | 7 | 4.77 ( 2.24 - 10.15 ) | 4.75 ( 20.08 ) | 4.63 ( 2.18 ) | 2.21 ( 0.62 ) |
| Investigations | Blood Creatine Phosphokinase Increased | 7 | 2.13 ( 1.01 - 4.51 ) | 2.13 ( 4.14 ) | 2.11 ( 1 ) | 1.08 ( -0.14 ) |
| Nervous System Disorders | Hypoaesthesia | 6 | 2.79 ( 1.24 - 6.27 ) | 2.78 ( 6.72 ) | 2.75 ( 1.22 ) | 1.46 ( 0.03 ) |
| Nervous System Disorders | Dysarthria | 6 | 7.58 ( 3.33 - 17.26 ) | 7.55 ( 32.35 ) | 7.21 ( 3.17 ) | 2.85 ( 0.8 ) |
| Nervous System Disorders | Paraesthesia | 6 | 3.16 ( 1.41 - 7.12 ) | 3.15 ( 8.64 ) | 3.11 ( 1.38 ) | 1.64 ( 0.14 ) |
| Nervous System Disorders | Balance Disorder | 6 | 5.62 ( 2.48 - 12.73 ) | 5.6 ( 21.8 ) | 5.42 ( 2.39 ) | 2.44 ( 0.61 ) |
| Vascular Disorders | Orthostatic Hypotension | 6 | 15.89 ( 6.81 - 37.05 ) | 15.83 ( 74.77 ) | 14.3 ( 6.13 ) | 3.84 ( 1.14 ) |
| Cardiac Disorders | Ventricular Tachycardia | 6 | 10.33 ( 4.5 - 23.71 ) | 10.29 ( 46.84 ) | 9.64 ( 4.2 ) | 3.27 ( 0.97 ) |
| Cardiac Disorders | Cardiac Failure Congestive | 6 | 6.07 ( 2.68 - 13.78 ) | 6.05 ( 24.26 ) | 5.84 ( 2.57 ) | 2.55 ( 0.66 ) |
| Investigations | Electrocardiogram Qt Prolonged | 6 | 4.01 ( 1.78 - 9.04 ) | 4 ( 13.11 ) | 3.91 ( 1.73 ) | 1.97 ( 0.35 ) |
| Nervous System Disorders | Amnesia | 5 | 9.42 ( 3.8 - 23.35 ) | 9.4 ( 35.13 ) | 8.86 ( 3.58 ) | 3.15 ( 0.71 ) |
| Vascular Disorders | Shock | 5 | 8.94 ( 3.61 - 22.11 ) | 8.91 ( 32.98 ) | 8.43 ( 3.41 ) | 3.08 ( 0.68 ) |
| Eye Disorders | Vision Blurred | 5 | 4.35 ( 1.78 - 10.62 ) | 4.34 ( 12.48 ) | 4.24 ( 1.74 ) | 2.08 ( 0.25 ) |
| Skin And Subcutaneous Tissue Disorders | Pain Of Skin | 5 | 21.5 ( 8.37 - 55.26 ) | 21.44 ( 84.28 ) | 18.68 ( 7.27 ) | 4.22 ( 0.96 ) |
| Cardiac Disorders | Arrhythmia | 5 | 4.81 ( 1.97 - 11.75 ) | 4.8 ( 14.53 ) | 4.67 ( 1.91 ) | 2.22 ( 0.33 ) |
| Renal And Urinary Disorders | Urinary Incontinence | 5 | 14.04 ( 5.59 - 35.29 ) | 14 ( 54.79 ) | 12.8 ( 5.09 ) | 3.68 ( 0.86 ) |
| Nervous System Disorders | Aphasia | 4 | 5.67 ( 2.08 - 15.43 ) | 5.66 ( 14.74 ) | 5.47 ( 2.01 ) | 2.45 ( 0.2 ) |
| Nervous System Disorders | Dyslalia | 4 | 91.71 ( 25.85 - 325.29 ) | 91.47 ( 214.78 ) | 55.28 ( 15.59 ) | 5.79 ( 0.65 ) |
| General Disorders And Administration Site Conditions | Feeling Abnormal | 4 | 4.66 ( 1.72 - 12.64 ) | 4.65 ( 11.1 ) | 4.53 ( 1.67 ) | 2.18 ( 0.08 ) |
| Gastrointestinal Disorders | Dry Mouth | 4 | 2.79 ( 1.04 - 7.52 ) | 2.79 ( 4.49 ) | 2.75 ( 1.02 ) | 1.46 ( -0.29 ) |
| Injury, Poisoning And Procedural Complications | Humerus Fracture | 4 | 39.3 ( 12.92 - 119.53 ) | 39.2 ( 115.83 ) | 30.71 ( 10.1 ) | 4.94 ( 0.68 ) |
| Nervous System Disorders | Neuralgia | 4 | 9.17 ( 3.33 - 25.26 ) | 9.15 ( 27.22 ) | 8.64 ( 3.14 ) | 3.11 ( 0.42 ) |
| General Disorders And Administration Site Conditions | Ill-Defined Disorder | 4 | 5.19 ( 1.91 - 14.1 ) | 5.18 ( 13 ) | 5.03 ( 1.85 ) | 2.33 ( 0.15 ) |
| Investigations | Troponin I Increased | 4 | 28.96 ( 9.84 - 85.22 ) | 28.89 ( 88.97 ) | 24.04 ( 8.17 ) | 4.59 ( 0.67 ) |
| Gastrointestinal Disorders | Hypoaesthesia Oral | 4 | 32.37 ( 10.88 - 96.3 ) | 32.29 ( 98.18 ) | 26.33 ( 8.85 ) | 4.72 ( 0.68 ) |
| Nervous System Disorders | Cerebral Haemorrhage | 4 | 4.2 ( 1.55 - 11.37 ) | 4.19 ( 9.43 ) | 4.1 ( 1.51 ) | 2.03 ( 0.02 ) |
| Cardiac Disorders | Cardiac Failure Acute | 4 | 6.63 ( 2.43 - 18.1 ) | 6.61 ( 18.19 ) | 6.35 ( 2.33 ) | 2.67 ( 0.28 ) |
| Cardiac Disorders | Left Ventricular Dysfunction | 4 | 6.71 ( 2.46 - 18.32 ) | 6.69 ( 18.48 ) | 6.43 ( 2.35 ) | 2.68 ( 0.29 ) |
| Injury, Poisoning And Procedural Complications | Fracture | 3 | 3.58 ( 1.14 - 11.29 ) | 3.58 ( 5.44 ) | 3.51 ( 1.12 ) | 1.81 ( -0.36 ) |
| Respiratory, Thoracic And Mediastinal Disorders | Wheezing | 3 | 5.73 ( 1.8 - 18.19 ) | 5.72 ( 11.21 ) | 5.53 ( 1.74 ) | 2.47 ( -0.11 ) |
| Musculoskeletal And Connective Tissue Disorders | Joint Effusion | 3 | 41.24 ( 11.34 - 150 ) | 41.16 ( 90.44 ) | 31.9 ( 8.77 ) | 5 ( 0.22 ) |
| Injury, Poisoning And Procedural Complications | Ligament Rupture | 3 | 206.21 ( 34.43 - 1234.98 ) | 205.82 ( 244.59 ) | 82.93 ( 13.85 ) | 6.37 ( 0.07 ) |
| Cardiac Disorders | Cardiotoxicity | 3 | 3.82 ( 1.21 - 12.03 ) | 3.81 ( 6.06 ) | 3.74 ( 1.18 ) | 1.9 ( -0.33 ) |
| Injury, Poisoning And Procedural Complications | Contusion | 3 | 8.41 ( 2.62 - 27.03 ) | 8.4 ( 18.44 ) | 7.97 ( 2.48 ) | 3 ( 0.03 ) |
| Nervous System Disorders | Neurotoxicity | 3 | 3.68 ( 1.17 - 11.6 ) | 3.68 ( 5.69 ) | 3.61 ( 1.14 ) | 1.85 ( -0.35 ) |
| Metabolism And Nutrition Disorders | Hyperuricaemia | 3 | 7.36 ( 2.3 - 23.55 ) | 7.35 ( 15.63 ) | 7.03 ( 2.2 ) | 2.81 ( -0.01 ) |
| Nervous System Disorders | Dyskinesia | 3 | 31.72 ( 9.03 - 111.43 ) | 31.66 ( 72.39 ) | 25.91 ( 7.38 ) | 4.7 ( 0.23 ) |
| Psychiatric Disorders | Mental Disorder | 3 | 6.65 ( 2.09 - 21.21 ) | 6.64 ( 13.71 ) | 6.38 ( 2 ) | 2.67 ( -0.05 ) |
| Nervous System Disorders | Hyperaesthesia | 3 | 14.22 ( 4.33 - 46.73 ) | 14.19 ( 33.35 ) | 12.96 ( 3.94 ) | 3.7 ( 0.16 ) |
| Cardiac Disorders | Palpitations | 3 | 4.21 ( 1.33 - 13.28 ) | 4.2 ( 7.1 ) | 4.11 ( 1.3 ) | 2.04 ( -0.27 ) |
| Renal And Urinary Disorders | Incontinence | 3 | 25.77 ( 7.5 - 88.55 ) | 25.73 ( 60.05 ) | 21.82 ( 6.35 ) | 4.45 ( 0.22 ) |
| Metabolism And Nutrition Disorders | Fluid Retention | 3 | 4.79 ( 1.51 - 15.17 ) | 4.79 ( 8.69 ) | 4.66 ( 1.47 ) | 2.22 ( -0.2 ) |
| Nervous System Disorders | Movement Disorder | 3 | 5.03 ( 1.59 - 15.93 ) | 5.02 ( 9.32 ) | 4.88 ( 1.54 ) | 2.29 ( -0.18 ) |
| Cardiac Disorders | Ventricular Arrhythmia | 3 | 31.72 ( 9.03 - 111.43 ) | 31.66 ( 72.39 ) | 25.91 ( 7.38 ) | 4.7 ( 0.23 ) |
| General Disorders And Administration Site Conditions | Generalised Oedema | 3 | 3.49 ( 1.11 - 11 ) | 3.49 ( 5.2 ) | 3.43 ( 1.09 ) | 1.78 ( -0.38 ) |
| Respiratory, Thoracic And Mediastinal Disorders | Pulmonary Hypertension | 3 | 7.64 ( 2.38 - 24.45 ) | 7.62 ( 16.36 ) | 7.27 ( 2.27 ) | 2.86 ( 0 ) |
| Investigations | Blood Pressure Decreased | 3 | 3.3 ( 1.05 - 10.37 ) | 3.29 ( 4.68 ) | 3.24 ( 1.03 ) | 1.7 ( -0.42 ) |
| Nervous System Disorders | Central Nervous System Lesion | 3 | 4.25 ( 1.35 - 13.42 ) | 4.24 ( 7.22 ) | 4.15 ( 1.31 ) | 2.05 ( -0.26 ) |
| Investigations | Electrocardiogram T Wave Inversion | 3 | 82.48 ( 19.69 - 345.44 ) | 82.33 ( 150.64 ) | 51.83 ( 12.38 ) | 5.7 ( 0.17 ) |
| Investigations | Electrocardiogram Abnormal | 3 | 24.26 ( 7.1 - 82.86 ) | 24.21 ( 56.76 ) | 20.73 ( 6.07 ) | 4.37 ( 0.22 ) |

Note: PT, preferred term; ROR, reporting odds ratio; PRR, proportional reporting ratio; EBGM, empirical Bayes geometric mean; EBGM05, lower fifth percentile of EBGM; IC, information component; IC025, lower limit of the 95% credibility interval; CI, confidence interval; χ², chi-square statistic.
