# Supplementary material for: Safety profile of entrectinib in NSCLC: Multi-source pharmacovigilance analysis using FAERS and JADER
Source: PLoS One. 2026 Sep 15;21(9):e0358331. doi: 10.1371/journal.pone.0358331 (PMC13577382; doi:10.1371/journal.pone.0358331)
Supplement: Supplementary file 5 [file pone.0358331.s005.docx]

S5 Table. Positive PT signals of entrectinib-associated adverse events in JADER

| **System Organ Class** | **PT** | **a** | **ROR(95%CI)** | **PRR(χ2)** | **EBGM(EBGM05)** | **IC(IC025)** |
| --- | --- | --- | --- | --- | --- | --- |
| Renal And Urinary Disorders | Renal Impairment | 29 | 8.18 ( 5.53 - 12.1 ) | 7.65 ( 157.14 ) | 7.17 ( 4.85 ) | 2.84 ( 1.16 ) |
| Cardiac Disorders | Cardiac Failure | 17 | 8.9 ( 5.36 - 14.75 ) | 8.55 ( 104.94 ) | 7.95 ( 4.79 ) | 2.99 ( 1.31 ) |
| Renal And Urinary Disorders | Renal Disorder | 16 | 21.6 ( 12.48 - 37.39 ) | 20.76 ( 249.24 ) | 17.33 ( 10.01 ) | 4.11 ( 2.42 ) |
| Nervous System Disorders | Cognitive Disorder | 16 | 78.29 ( 40.54 - 151.22 ) | 75.15 ( 665.01 ) | 43.08 ( 22.31 ) | 5.43 ( 3.69 ) |
| Nervous System Disorders | Syncope | 14 | 89.45 ( 43.35 - 184.58 ) | 86.3 ( 630.03 ) | 46.49 ( 22.53 ) | 5.54 ( 3.79 ) |
| Investigations | Blood Creatinine Increased | 13 | 24.99 ( 13.51 - 46.21 ) | 24.19 ( 232.5 ) | 19.62 ( 10.61 ) | 4.29 ( 2.59 ) |
| Investigations | Neutrophil Count Decreased | 12 | 2.16 ( 1.21 - 3.86 ) | 2.12 ( 7.1 ) | 2.1 ( 1.18 ) | 1.07 ( -0.61 ) |
| Investigations | Electrocardiogram Qt Prolonged | 9 | 5.35 ( 2.72 - 10.54 ) | 5.25 ( 29.55 ) | 5.04 ( 2.56 ) | 2.33 ( 0.65 ) |
| Hepatobiliary Disorders | Hepatic Function Abnormal | 9 | 2.12 ( 1.09 - 4.13 ) | 2.09 ( 5.09 ) | 2.07 ( 1.06 ) | 1.05 ( -0.63 ) |
| Cardiac Disorders | Myocarditis | 9 | 5.91 ( 3 - 11.67 ) | 5.8 ( 33.92 ) | 5.54 ( 2.8 ) | 2.47 ( 0.78 ) |
| Blood And Lymphatic System Disorders | Anaemia | 9 | 3.81 ( 1.94 - 7.47 ) | 3.75 ( 17.56 ) | 3.65 ( 1.86 ) | 1.87 ( 0.18 ) |
| Blood And Lymphatic System Disorders | Neutropenia | 8 | 2.53 ( 1.24 - 5.14 ) | 2.5 ( 7.06 ) | 2.46 ( 1.21 ) | 1.3 ( -0.38 ) |
| Nervous System Disorders | Ataxia | 7 | 351.45 ( 72.78 - 1697.24 ) | 345.21 ( 534.01 ) | 77.49 ( 16.05 ) | 6.28 ( 4.37 ) |
| Nervous System Disorders | Dizziness | 7 | 14.33 ( 6.45 - 31.83 ) | 14.09 ( 74.59 ) | 12.45 ( 5.61 ) | 3.64 ( 1.92 ) |
| Cardiac Disorders | Cardiac Disorder | 7 | 175.72 ( 51.23 - 602.72 ) | 172.6 ( 434.4 ) | 63.4 ( 18.48 ) | 5.99 ( 4.12 ) |
| Psychiatric Disorders | Delirium | 5 | 8.75 ( 3.49 - 21.95 ) | 8.65 ( 31.16 ) | 8.03 ( 3.2 ) | 3.01 ( 1.3 ) |
| Cardiac Disorders | Cardiomyopathy | 5 | 13.49 ( 5.27 - 34.5 ) | 13.33 ( 50.28 ) | 11.86 ( 4.64 ) | 3.57 ( 1.84 ) |
| Gastrointestinal Disorders | Nausea | 5 | 3.01 ( 1.23 - 7.38 ) | 2.99 ( 6.45 ) | 2.93 ( 1.2 ) | 1.55 ( -0.14 ) |
| Investigations | Troponin I Increased | 4 | 79.71 ( 21.32 - 297.95 ) | 78.9 ( 170.97 ) | 44.28 ( 11.85 ) | 5.47 ( 3.56 ) |
| General Disorders And Administration Site Conditions | Gait Disturbance | 4 | 22.13 ( 7.46 - 65.7 ) | 21.92 ( 65.37 ) | 18.11 ( 6.1 ) | 4.18 ( 2.4 ) |
| Gastrointestinal Disorders | Vomiting | 4 | 6.52 ( 2.36 - 18.03 ) | 6.47 ( 17.38 ) | 6.13 ( 2.22 ) | 2.62 ( 0.91 ) |
| Investigations | Ejection Fraction Decreased | 4 | 17.32 ( 5.96 - 50.32 ) | 17.15 ( 51.87 ) | 14.76 ( 5.08 ) | 3.88 ( 2.12 ) |
| Nervous System Disorders | Taste Disorder | 4 | 13.28 ( 4.65 - 37.86 ) | 13.15 ( 39.66 ) | 11.72 ( 4.11 ) | 3.55 ( 1.81 ) |
| Vascular Disorders | Shock | 3 | 11.46 ( 3.45 - 38.02 ) | 11.38 ( 25.49 ) | 10.31 ( 3.11 ) | 3.37 ( 1.61 ) |
| Metabolism And Nutrition Disorders | Hyperuricaemia | 3 | 59.63 ( 14.2 - 250.37 ) | 59.18 ( 107.26 ) | 37.36 ( 8.9 ) | 5.22 ( 3.29 ) |
| General Disorders And Administration Site Conditions | Oedema Peripheral | 3 | 8.05 ( 2.47 - 26.22 ) | 8 ( 17.01 ) | 7.47 ( 2.29 ) | 2.9 ( 1.17 ) |
| Cardiac Disorders | Arrhythmia | 3 | 11.92 ( 3.58 - 39.64 ) | 11.84 ( 26.59 ) | 10.67 ( 3.21 ) | 3.42 ( 1.66 ) |
| Cardiac Disorders | Cardiac Failure Acute | 3 | 9.61 ( 2.93 - 31.57 ) | 9.54 ( 20.94 ) | 8.79 ( 2.68 ) | 3.14 ( 1.39 ) |
| Vascular Disorders | Hypotension | 3 | 11.04 ( 3.33 - 36.53 ) | 10.96 ( 24.45 ) | 9.96 ( 3.01 ) | 3.32 ( 1.57 ) |
| Cardiac Disorders | Left Ventricular Dysfunction | 3 | 99.38 ( 20 - 493.94 ) | 98.63 ( 144.98 ) | 49.82 ( 10.02 ) | 5.64 ( 3.64 ) |
| Respiratory, Thoracic And Mediastinal Disorders | Pulmonary Embolism | 3 | 3.67 ( 1.16 - 11.68 ) | 3.65 ( 5.59 ) | 3.56 ( 1.12 ) | 1.83 ( 0.13 ) |

Note: PT, preferred term; ROR, reporting odds ratio; PRR, proportional reporting ratio; EBGM, empirical Bayes geometric mean; EBGM05, lower fifth percentile of EBGM; IC, information component; IC025, lower limit of the 95% credibility interval; CI, confidence interval; χ², chi-square statistic.
