# Supplementary material for: Safety profile of entrectinib in NSCLC: Multi-source pharmacovigilance analysis using FAERS and JADER
Source: PLoS One. 2026 Sep 15;21(9):e0358331. doi: 10.1371/journal.pone.0358331 (PMC13577382; doi:10.1371/journal.pone.0358331)
Supplement: Supplementary file 6 [file pone.0358331.s006.docx]

S6 Table. Age-stratified subgroup analysis of positive PT signals in the FAERS database

| **PT** | **<65** | **≥65** | **ROR** | **RORL** | **RORU** |
| --- | --- | --- | --- | --- | --- |
| Disease Progression | 21 | 12 | 2.27 | 1.10 | 4.70 |
| Dizziness | 18 | 24 | 0.93 | 0.49 | 1.75 |
| Taste Disorder | 12 | 25 | 0.58 | 0.29 | 1.17 |
| Renal Impairment | 11 | 29 | 0.45 | 0.22 | 0.92 |
| Blood Creatinine Increased | 11 | 25 | 0.53 | 0.26 | 1.09 |
| Hepatic Function Abnormal | 11 | 3 | 4.71 | 1.30 | 17.04 |
| Syncope | 9 | 5 | 2.28 | 0.76 | 6.88 |
| Cardiac Failure | 8 | 17 | 0.57 | 0.24 | 1.35 |
| Pleural Effusion | 8 | 5 | 2.02 | 0.65 | 6.24 |
| Metastases To Central Nervous System | 8 | 2 | 5.09 | 1.07 | 24.18 |
| Ataxia | 7 | 10 | 0.87 | 0.33 | 2.31 |
| Weight Increased | 7 | 5 | 1.76 | 0.55 | 5.61 |
| Pericardial Effusion | 7 | 3 | 2.95 | 0.76 | 11.52 |
| Seizure | 6 | - | - | - | - |
| Constipation | 6 | 7 | 1.07 | 0.35 | 3.21 |
| Hypotension | 6 | 7 | 1.07 | 0.35 | 3.21 |
| Dysgeusia | 6 | 2 | 3.79 | 0.76 | 18.93 |
| Blood Creatine Phosphokinase Increased | 5 | - | - | - | - |
| Cognitive Disorder | 5 | 14 | 0.43 | 0.15 | 1.22 |
| Oedema | 5 | 6 | 1.04 | 0.31 | 3.43 |
| Myocarditis | 4 | 6 | 0.83 | 0.23 | 2.96 |
| Arthralgia | 4 | 5 | 0.99 | 0.26 | 3.74 |
| Ventricular Tachycardia | 4 | 2 | 2.51 | 0.46 | 13.80 |
| Neurotoxicity | 3 | - | - | - | - |
| Palpitations | 3 | - | - | - | - |
| Ejection Fraction Decreased | 3 | 5 | 0.74 | 0.18 | 3.14 |
| Cardiac Disorder | 3 | 5 | 0.74 | 0.18 | 3.14 |
| Muscular Weakness | 3 | 3 | 1.25 | 0.25 | 6.22 |
| Urinary Retention | 3 | 3 | 1.25 | 0.25 | 6.22 |
| Hypoaesthesia | 3 | 2 | 1.88 | 0.31 | 11.30 |
| Balance Disorder | 3 | 2 | 1.88 | 0.31 | 11.30 |
| Cardiac Failure Congestive | 3 | 2 | 1.88 | 0.31 | 11.30 |
| Electrocardiogram Qt Prolonged | 3 | 2 | 1.88 | 0.31 | 11.30 |
| Dysphagia | 3 | 1 | 3.76 | 0.39 | 36.36 |
| Pain Of Skin | 2 | - | - | - | - |
| Feeling Abnormal | 2 | - | - | - | - |
| Ill-Defined Disorder | 2 | - | - | - | - |
| Joint Effusion | 2 | - | - | - | - |
| Dyskinesia | 2 | - | - | - | - |
| Gait Disturbance | 2 | 12 | 0.20 | 0.04 | 0.91 |
| Renal Disorder | 2 | 12 | 0.20 | 0.04 | 0.91 |
| Fall | 2 | 8 | 0.31 | 0.06 | 1.45 |
| Cardiomyopathy | 2 | 6 | 0.41 | 0.08 | 2.05 |
| Shock | 2 | 2 | 1.25 | 0.17 | 8.90 |
| Arrhythmia | 2 | 2 | 1.25 | 0.17 | 8.90 |
| Amnesia | 2 | 1 | 2.50 | 0.23 | 27.70 |
| Vision Blurred | 2 | 1 | 2.50 | 0.23 | 27.70 |
| Dyslalia | 2 | 1 | 2.50 | 0.23 | 27.70 |
| Dry Mouth | 2 | 1 | 2.50 | 0.23 | 27.70 |
| Troponin I Increased | 2 | 1 | 2.50 | 0.23 | 27.70 |
| Fluid Retention | 2 | 1 | 2.50 | 0.23 | 27.70 |
| Ventricular Arrhythmia | 2 | 1 | 2.50 | 0.23 | 27.70 |
| Electrocardiogram Abnormal | 2 | 1 | 2.50 | 0.23 | 27.70 |
| Visual Impairment | 1 | 4 | 0.31 | 0.03 | 2.78 |
| Memory Impairment | 1 | 4 | 0.31 | 0.03 | 2.78 |
| Dysarthria | 1 | 4 | 0.31 | 0.03 | 2.78 |
| Paraesthesia | 1 | 3 | 0.41 | 0.04 | 3.99 |
| Orthostatic Hypotension | 1 | 3 | 0.41 | 0.04 | 3.99 |
| Hypoaesthesia Oral | 1 | 2 | 0.62 | 0.06 | 6.88 |
| Cardiac Failure Acute | 1 | 2 | 0.62 | 0.06 | 6.88 |
| Left Ventricular Dysfunction | 1 | 2 | 0.62 | 0.06 | 6.88 |
| Cardiotoxicity | 1 | 2 | 0.62 | 0.06 | 6.88 |
| Pulmonary Hypertension | 1 | 2 | 0.62 | 0.06 | 6.88 |
| Blood Pressure Decreased | 1 | 2 | 0.62 | 0.06 | 6.88 |
| Aphasia | 1 | 1 | 1.24 | 0.08 | 19.99 |
| Neuralgia | 1 | 1 | 1.24 | 0.08 | 19.99 |
| Wheezing | 1 | 1 | 1.24 | 0.08 | 19.99 |
| Ligament Rupture | 1 | 1 | 1.24 | 0.08 | 19.99 |
| Hyperuricaemia | 1 | 1 | 1.24 | 0.08 | 19.99 |
| Movement Disorder | 1 | 1 | 1.24 | 0.08 | 19.99 |
| Generalised Oedema | 1 | 1 | 1.24 | 0.08 | 19.99 |
| Electrocardiogram T Wave Inversion | 1 | 1 | 1.24 | 0.08 | 19.99 |
| Urinary Incontinence | - | 4 | - | - | - |
| Humerus Fracture | - | 3 | - | - | - |
| Cerebral Haemorrhage | - | 2 | - | - | - |
| Fracture | - | 1 | - | - | - |
| Contusion | - | 3 | - | - | - |
| Mental Disorder | - | 1 | - | - | - |
| Hyperaesthesia | - | 1 | - | - | - |
| Incontinence | - | 3 | - | - | - |
| Central Nervous System Lesion | - | 2 | - | - | - |

Note: PT, preferred term; ROR, reporting odds ratio; RORL, lower limit of the 95% confidence interval; RORU, upper limit of the 95% confidence interval. A dash indicates that the ROR was not calculated because one subgroup had zero reported events.
