# Supplementary material for: Safety profile of entrectinib in NSCLC: Multi-source pharmacovigilance analysis using FAERS and JADER
Source: PLoS One. 2026 Sep 15;21(9):e0358331. doi: 10.1371/journal.pone.0358331 (PMC13577382; doi:10.1371/journal.pone.0358331)
Supplement: Supplementary file 7 [file pone.0358331.s007.docx]

S7 Table. Sex-stratified subgroup analysis of positive PT signals in the FAERS database

| **PT** | **Female** | **Male** | **ROR** | **RORL** | **RORU** |
| --- | --- | --- | --- | --- | --- |
| Dizziness | 34 | 19 | 1.31 | 0.73 | 2.34 |
| Renal Impairment | 25 | 17 | 1.06 | 0.56 | 2.00 |
| Taste Disorder | 22 | 19 | 0.82 | 0.44 | 1.54 |
| Disease Progression | 20 | 14 | 1.02 | 0.51 | 2.06 |
| Cardiac Failure | 16 | 10 | 1.15 | 0.52 | 2.58 |
| Ataxia | 16 | 2 | 5.92 | 1.35 | 25.97 |
| Cognitive Disorder | 15 | 8 | 1.36 | 0.57 | 3.24 |
| Blood Creatinine Increased | 14 | 20 | 0.48 | 0.24 | 0.97 |
| Weight Increased | 11 | 6 | 1.32 | 0.48 | 3.62 |
| Gait Disturbance | 10 | 4 | 1.81 | 0.56 | 5.83 |
| Oedema | 10 | 3 | 2.42 | 0.66 | 8.88 |
| Metastases To Central Nervous System | 10 | 2 | 3.65 | 0.79 | 16.77 |
| Hypotension | 8 | 6 | 0.95 | 0.33 | 2.78 |
| Syncope | 7 | 7 | 0.71 | 0.25 | 2.05 |
| Hepatic Function Abnormal | 7 | 6 | 0.83 | 0.28 | 2.50 |
| Pericardial Effusion | 7 | 5 | 1.00 | 0.32 | 3.19 |
| Arthralgia | 7 | 4 | 1.26 | 0.36 | 4.34 |
| Dysgeusia | 7 | 2 | 2.53 | 0.52 | 12.28 |
| Memory Impairment | 6 | - | - | - | - |
| Renal Disorder | 6 | 8 | 0.53 | 0.18 | 1.55 |
| Myocarditis | 6 | 5 | 0.86 | 0.26 | 2.84 |
| Cardiomyopathy | 6 | 4 | 1.08 | 0.30 | 3.85 |
| Fall | 6 | 4 | 1.08 | 0.30 | 3.85 |
| Pleural Effusion | 5 | 11 | 0.32 | 0.11 | 0.92 |
| Constipation | 5 | 9 | 0.39 | 0.13 | 1.18 |
| Hypoaesthesia | 5 | 1 | 3.61 | 0.42 | 31.10 |
| Balance Disorder | 5 | 1 | 3.61 | 0.42 | 31.10 |
| Cardiac Failure Congestive | 5 | 1 | 3.61 | 0.42 | 31.10 |
| Aphasia | 4 | - | - | - | - |
| Muscular Weakness | 4 | 2 | 1.44 | 0.26 | 7.90 |
| Paraesthesia | 4 | 1 | 2.88 | 0.32 | 25.94 |
| Electrocardiogram Qt Prolonged | 4 | 1 | 2.88 | 0.32 | 25.94 |
| Urinary Incontinence | 4 | 1 | 2.88 | 0.32 | 25.94 |
| Dyslalia | 3 | - | - | - | - |
| Feeling Abnormal | 3 | - | - | - | - |
| Cerebral Haemorrhage | 3 | - | - | - | - |
| Ejection Fraction Decreased | 3 | 8 | 0.26 | 0.07 | 1.00 |
| Urinary Retention | 3 | 5 | 0.43 | 0.10 | 1.80 |
| Cardiac Disorder | 3 | 4 | 0.53 | 0.12 | 2.40 |
| Visual Impairment | 3 | 4 | 0.53 | 0.12 | 2.40 |
| Dysphagia | 3 | 3 | 0.71 | 0.14 | 3.56 |
| Ventricular Tachycardia | 3 | 3 | 0.71 | 0.14 | 3.56 |
| Arrhythmia | 3 | 1 | 2.16 | 0.22 | 20.85 |
| Ill-Defined Disorder | 3 | 1 | 2.16 | 0.22 | 20.85 |
| Hypoaesthesia Oral | 3 | 1 | 2.16 | 0.22 | 20.85 |
| Ligament Rupture | 2 | - | - | - | - |
| Cardiotoxicity | 2 | - | - | - | - |
| Hyperaesthesia | 2 | - | - | - | - |
| Movement Disorder | 2 | - | - | - | - |
| Generalised Oedema | 2 | - | - | - | - |
| Pulmonary Hypertension | 2 | - | - | - | - |
| Central Nervous System Lesion | 2 | - | - | - | - |
| Blood Creatine Phosphokinase Increased | 2 | 4 | 0.36 | 0.06 | 1.95 |
| Orthostatic Hypotension | 2 | 3 | 0.48 | 0.08 | 2.86 |
| Dysarthria | 2 | 2 | 0.71 | 0.10 | 5.11 |
| Amnesia | 2 | 2 | 0.71 | 0.10 | 5.11 |
| Shock | 2 | 2 | 0.71 | 0.10 | 5.11 |
| Pain Of Skin | 2 | 2 | 0.71 | 0.10 | 5.11 |
| Vision Blurred | 2 | 1 | 1.43 | 0.13 | 15.90 |
| Cardiac Failure Acute | 2 | 1 | 1.43 | 0.13 | 15.90 |
| Left Ventricular Dysfunction | 2 | 1 | 1.43 | 0.13 | 15.90 |
| Wheezing | 2 | 1 | 1.43 | 0.13 | 15.90 |
| Neurotoxicity | 2 | 1 | 1.43 | 0.13 | 15.90 |
| Palpitations | 2 | 1 | 1.43 | 0.13 | 15.90 |
| Blood Pressure Decreased | 2 | 1 | 1.43 | 0.13 | 15.90 |
| Fracture | 1 | - | - | - | - |
| Dry Mouth | 1 | 3 | 0.24 | 0.02 | 2.29 |
| Humerus Fracture | 1 | 2 | 0.36 | 0.03 | 3.95 |
| Neuralgia | 1 | 2 | 0.36 | 0.03 | 3.95 |
| Joint Effusion | 1 | 2 | 0.36 | 0.03 | 3.95 |
| Dyskinesia | 1 | 2 | 0.36 | 0.03 | 3.95 |
| Incontinence | 1 | 2 | 0.36 | 0.03 | 3.95 |
| Fluid Retention | 1 | 2 | 0.36 | 0.03 | 3.95 |
| Ventricular Arrhythmia | 1 | 2 | 0.36 | 0.03 | 3.95 |
| Electrocardiogram T Wave Inversion | 1 | 2 | 0.36 | 0.03 | 3.95 |
| Hyperuricaemia | 1 | 1 | 0.72 | 0.04 | 11.49 |
| Mental Disorder | 1 | 1 | 0.72 | 0.04 | 11.49 |
| Seizure | - | 8 | - | - | - |
| Troponin I Increased | - | 3 | - | - | - |
| Contusion | - | 3 | - | - | - |
| Electrocardiogram Abnormal | - | 3 | - | - | - |

Note: PT, preferred term; ROR, reporting odds ratio; RORL, lower limit of the 95% confidence interval; RORU, upper limit of the 95% confidence interval. A dash indicates that the ROR was not calculated because one subgroup had zero reported events.
