# Supplementary material for: Safety profile of entrectinib in NSCLC: Multi-source pharmacovigilance analysis using FAERS and JADER
Source: PLoS One. 2026 Sep 15;21(9):e0358331. doi: 10.1371/journal.pone.0358331 (PMC13577382; doi:10.1371/journal.pone.0358331)
Supplement: Supplementary file 8 [file pone.0358331.s008.docx]

S8 Table. Sex-stratified subgroup analysis of positive PT signals in the JADER database

| **PT** | **Female** | **Male** | **ROR** | **RORL** | **RORU** |
| --- | --- | --- | --- | --- | --- |
| Renal impairment | 20 | 8 | 1.47 | 0.61 | 3.50 |
| Cardiac failure | 5 | 10 | 0.32 | 0.10 | 0.96 |
| Renal disorder | 8 | 8 | 0.62 | 0.23 | 1.73 |
| Cognitive disorder | 13 | 3 | 2.64 | 0.73 | 9.56 |
| Syncope | 8 | 6 | 0.83 | 0.28 | 2.48 |
| Blood creatinine increased | 5 | 8 | 0.40 | 0.13 | 1.25 |
| Neutrophil count decreased | 8 | 4 | 1.25 | 0.36 | 4.28 |
| Electrocardiogram QT prolonged | 6 | 1 | 3.78 | 0.45 | 31.93 |
| Hepatic function abnormal | 4 | 4 | 0.64 | 0.15 | 2.61 |
| Myocarditis | 3 | 5 | 0.38 | 0.09 | 1.64 |
| Anaemia | 6 | 3 | 1.26 | 0.31 | 5.17 |
| Neutropenia | 6 | 2 | 1.89 | 0.37 | 9.58 |
| Ataxia | 6 | 1 | 3.78 | 0.45 | 31.93 |
| Dizziness | 5 | 2 | 1.58 | 0.30 | 8.33 |
| Cardiac disorder | 2 | 3 | 0.43 | 0.07 | 2.61 |
| Delirium | 5 | - | - | - | - |
| Cardiomyopathy | 4 | 1 | 2.54 | 0.28 | 23.10 |
| Nausea | 4 | 1 | 2.54 | 0.28 | 23.10 |
| Troponin I increased | - | 4 | - | - | - |
| Gait disturbance | 4 | - | - | - | - |
| Vomiting | 3 | 1 | 1.91 | 0.20 | 18.68 |
| Ejection fraction decreased | - | 4 | - | - | - |
| Taste disorder | 2 | 2 | 0.64 | 0.09 | 4.63 |
| Shock | 1 | 2 | 0.32 | 0.03 | 3.60 |
| Hyperuricaemia | 3 | - | - | - | - |
| Oedema peripheral | 2 | 1 | 1.28 | 0.11 | 14.33 |
| Arrhythmia | 2 | 1 | 1.28 | 0.11 | 14.33 |
| Cardiac failure acute | 2 | 1 | 1.28 | 0.11 | 14.33 |
| Hypotension | 1 | 2 | 0.32 | 0.03 | 3.60 |
| Left ventricular dysfunction | 2 | 1 | 1.28 | 0.11 | 14.33 |
| Pulmonary embolism | 1 | 2 | 0.32 | 0.03 | 3.60 |

Note: PT, preferred term; ROR, reporting odds ratio; RORL, lower limit of the 95% confidence interval; RORU, upper limit of the 95% confidence interval. A dash indicates that the ROR was not calculated because one subgroup had zero reported events.
