# Supplementary material for: Safety profile of entrectinib in NSCLC: Multi-source pharmacovigilance analysis using FAERS and JADER
Source: PLoS One. 2026 Sep 15;21(9):e0358331. doi: 10.1371/journal.pone.0358331 (PMC13577382; doi:10.1371/journal.pone.0358331)
Supplement: Supplementary file 9 [file pone.0358331.s011.docx]

The research has been conducted with rigorous methodology. The analysis of Entrectinib across both pharmacovigilance databases, FAERS and JADER, has been well executed, including four disproportionality analyses: ROR, PRR, BCPNN, and MGPS. The multi-layered analysis of the drug at both SOC and PT levels, age-subgroup analysis, sex-subgroup analysis, and Time-to-onset analysis have strengthened this study. However, several important improvements are required before the research is published.

1. **The novelty appears to be somewhat overstated.**

In the discussion, the authors emphasise neurological, cardiac, and renal toxicities as major findings; however, these are already recognised adverse effects of entrectinib. This includes the research paper

Li, W., Wen, K., Zhu, W., & Luo, S. (2023). A Real-World Analysis of Tyrosine Receptor Kinase Inhibitor-Related Toxicities in Cancer Treatment. Personalized Medicine, 20(6), 485–491. <https://doi.org/10.2217/pme-2023-0072>

The discussion should be revised. The manuscript would benefit more by emphasising the novelty of the present research, for example, by distinguishing it from previously established toxicity findings. The novelty of the present study includes cross-database replication, time to onset analysis showing adverse effects within 13 days, and demographic differences, exhibiting that older people have more renal problems and females have more ataxia, unusual findings or such as Dyslalia, Troponin increase, Left ventricular dysfunction and Orthostatic hypotension

2. **Disease progression is problematic.**

The authors found in the FAERS analysis “Disease progression (n = 42; ROR 3.35) and discussed it as the PT (Preferred term) signals. However, “disease progression” is not a side effect caused by entrectinib. The disease progression might have happened due to treatment failure, due to the aggressiveness of cancer, or because the patient has become resistant, rather than due to the toxicity of the drug. The authors are required to clarify the rationale for interpreting disease progression as an adverse-event signal and to discuss the potential for confounding by indication.

3. **Mechanistic discussion is speculative.**

The authors included a statement in the discussion, “Entrectinib-associated toxicity may involve higher-order neurologic function, motor coordination, and balance control." And "Taste disorder may serve as an early indicator of broader neurologic tolerability issues”. However, none of the investigations, such as Brain imaging, Neurophysiology, molecular pathways, or animal experiments, were conducted in the study to conclude such statements.

4. **The confidence intervals and reported RORs are large.**

Some RORs were found to be enormous, such as

1. In the FAERS PT analysis- cardiac disorder (n = 7; ROR 175.72, 95% CI 51.23–602.72), dyslalia (n = 4; ROR 91.71, 95% CI 25.85–325.29), electrocardiogram T wave inversion (n = 3; ROR 82.48, 95% CI 19.69–345.44), ligament rupture (n = 3; ROR 206.21, 95% CI 34.43–1234.98), joint effusion (n = 3; ROR 41.24, 95% CI 11.34–150.00), urinary incontinence (n = 5
2. In the JADER PT analysis ataxia (n = 7; ROR 351.45, 95% CI 72.78–1697.24), Syncope (n = 14; ROR 89.45, 95% CI 43.35-184.58), Left ventricular dysfunction ROR 99, cardiac disorder (n = 7; ROR 175.72, 95% CI 51.23–602.72), troponin I increased (n = 4; ROR 79.71, 95% CI 21.32–297.95), left ventricular dysfunction (n = 3; ROR 99.38, 95% CI 20.00–493.94), hyperuricaemia (n = 3; ROR 59.63, 95% CI 14.20–250.37)

The authors are required to discuss small cell counts, statistical instability, and reporting sensitivity more explicitly,

5. **Multiple Testing Issue**

The authors are also required to add a limitation in the discussion, acknowledging that some rare signals may be real, while others may appear by chance, given that they have tested numerous adverse events. For example, ligament rupture, joint effusion, left ventricular dysfunction and hyperuricemia all had only 3 cases and large RORs. These may not be true safety signals. The authors are also required to discuss multiplicity and false-positive signal inflation, as they conducted many PT in both FAERS and JADER.

6. **Time-to-onset interpretation**

The authors have included only about 203 cases out of 520 for the time-to-onset analysis due to missing dates. More than half of the data (about 61%) was not involved in the time-to-onset analysis. If feasible, the authors can include a comparison of baseline characteristics between included and excluded cases to evaluate selection bias. If both groups look similar among cases, then the TTO analysis is reasonable, and the 203 cases are representative of all the 520 cases. If the two groups are different, the TTO findings may be biased.

7. **Repetitive points in Discussion**

The discussion is also somewhat repetitive. Most points have been written many times, such as neurological toxicity, cardio and renal toxicity, and cross-database replication. I would recommend shortening the discussion by 15%.
