# Supplementary material for: Safety profile of entrectinib in NSCLC: Multi-source pharmacovigilance analysis using FAERS and JADER
Source: PLoS One. 2026 Sep 15;21(9):e0358331. doi: 10.1371/journal.pone.0358331 (PMC13577382; doi:10.1371/journal.pone.0358331)
Supplement: Supplementary file 10 [file pone.0358331.s013.docx]

**Response Letter**

Dear Editor and Reviewers,

On behalf of all co-authors, we sincerely thank the editor and reviewers for their thoughtful, detailed, and constructive comments on our manuscript entitled “Safety profile of entrectinib in NSCLC: multi-source pharmacovigilance analysis using FAERS and JADER.” These comments were very helpful for improving the methodological clarity, presentation of pharmacovigilance findings, interpretation of disproportionality signals, and alignment with PLOS ONE reporting requirements.

In the revised manuscript, we clarified the data sources and reference set, revised the Abstract, expanded the interpretation of neurologic, cardiac, and renal reporting signals, emphasized the hypothesis-generating nature of disproportionality analyses, added a summary table of cross-database replicated PT-level signals, revised the Data Availability Statement, and provided de-duplicated event-level analytic datasets as Supporting Information. We also revised the figure captions, Supporting Information captions, and file names according to PLOS ONE requirements.

**Point-by-point responses**

**Reviewer #1**

**Comment 1.1. Consider including more quantitative results, such as specific RORs for key PTs, in the abstract.**

Response: Thank you for this helpful suggestion. We revised the Abstract to include quantitative ROR estimates for the major system organ class-level and representative shared preferred-term-level signals. Specifically, we added ROR values for nervous system disorders, cardiac disorders, renal and urinary disorders, and representative shared PT signals such as dizziness, renal impairment, blood creatinine increased, cardiac failure, cognitive disorder, ataxia, syncope, myocarditis, electrocardiogram QT prolonged, and hyperuricaemia. We also revised the wording to emphasize that these findings represent pharmacovigilance signals rather than incidence or causal effects.

**Comment 1.2. Add a summary table of the most significant PT-level signals shared across both databases.**

Response: Thank you for this recommendation. We added Table 3, entitled “Cross-database replicated PT-level signals of entrectinib-associated adverse event reports in FAERS and JADER.” This table summarizes all 23 PT-level signals shared between FAERS and JADER, including event counts and ROR estimates with 95% confidence intervals in both databases. We believe this table improves readability and directly highlights the most reproducible component of the analysis.

**Reviewer #2**

**Comment 2.1. The clinical interpretation is superficial, and deeper interpretation of neurologic, cardiac, renal, and taste-related signals is needed.**

Response: Thank you for this important comment. We agree that the clinical interpretation of the major signal clusters should be strengthened, while avoiding overstatement of causality. We therefore revised the Discussion to provide a more detailed but cautious interpretation of the neurologic, cardiac, renal, and metabolic signals. Specifically, we discussed that entrectinib, as a TRK/ROS1/ALK inhibitor with intracranial antitumor activity, provides a biologically plausible context for the repeated reporting of neurologic events such as dizziness, cognitive disorder, ataxia, gait disturbance, syncope, and taste disorder. We also expanded the interpretation of cardiac signals, including cardiac failure, cardiomyopathy, myocarditis, arrhythmia, electrocardiogram QT prolonged, troponin I increased, and left ventricular dysfunction, and discussed possible overlapping explanations such as kinase-inhibition-related effects, electrophysiologic vulnerability, baseline cardiovascular disease, prior anticancer therapy, and concomitant QT-prolonging medications. In addition, we clarified that renal impairment, renal disorder, blood creatinine increased, and hyperuricaemia may reflect multiple pathways, including dehydration, hemodynamic instability, concomitant nephrotoxic drugs, tumor-related clinical deterioration, tumor lysis-related metabolic disturbance, or progression of baseline renal dysfunction. At the same time, we explicitly stated that FAERS and JADER do not provide sufficient clinical, imaging, laboratory, electrocardiographic, or mechanistic information to establish specific causal pathways. Therefore, these findings were interpreted as pharmacovigilance signals and monitoring priorities rather than mechanistically confirmed toxicities.

**Comment 2.2. Database-specific signals deserve more attention.**

Response: We agree. We expanded the interpretation of database-specific PT signals. We now discuss that the larger number of FAERS-specific PTs compared with JADER-specific PTs may reflect differences in database size, reporting sources, regional prescribing patterns, clinical practice, regulatory context, coding habits, reporting sensitivity, and random variation, particularly for rare events. We also emphasize that database-specific signals should be interpreted as exploratory findings requiring further validation.

**Comment 2.3. Annual reporting trends may reflect reporting bias.**

Response: Thank you for highlighting this issue. We revised the Results and Discussion to clarify that annual report counts describe reporting activity rather than true changes in the incidence of adverse events. We added that reporting trends may be influenced by drug utilization, clinician awareness, regulatory communications, media attention, public concern, and pharmacovigilance practices.

**Comment 2.4. The wording should consistently emphasize that disproportionality analyses generate hypotheses rather than establish causality.**

Response: We agree. Throughout the revised Abstract, Results, Discussion, Limitations, and Conclusion, we replaced causal or definitive language with wording such as “disproportional reporting signals,” “hypothesis-generating,” and “pharmacovigilance signals.” We explicitly state that the findings should not be interpreted as evidence of incidence, absolute risk, or confirmed causal adverse drug reactions.

**Reviewer #3**

**Comment 3.1. Please define abbreviations at first use.**

Response: Thank you for this comment. We reviewed the manuscript and revised abbreviation definitions at first appearance in both the Abstract and main text. We defined FAERS, JADER, NSCLC, SOC, PT, ROR, PRR, BCPNN, MGPS, TTO, and other relevant abbreviations where they first appear.

**Comment 3.2. Multiple comparisons may inflate false-positive signals; please emphasize cross-database replicated signals over single-database or single-method signals.**

Response: We agree. We added a statement in the Methods explaining that no formal multiplicity adjustment was applied because disproportionality analysis is intended for pharmacovigilance signal detection rather than confirmatory hypothesis testing. We also revised the Results, Discussion, and Limitations to emphasize the 23 cross-database replicated PTs as the most reproducible component of the analysis, while interpreting database-specific, single-method, and sparse-count signals as exploratory and hypothesis-generating.

**Comment 3.3. The comparator group is not defined, and a sensitivity analysis restricted to other antineoplastic agents was suggested.**

Response: Thank you for this important methodological point. We clarified that disproportionality analyses were conducted within the NSCLC reporting background rather than against the entire FAERS or JADER database. The target group consisted of reports in which entrectinib was recorded as the primary suspect drug for NSCLC-related indications. The reference set consisted of NSCLC-related reports involving all other drugs after excluding entrectinib-related reports. Thus, the ROR estimates reflect disproportionate reporting relative to other drug reports within the same disease-reporting background. Because the publicly available spontaneous reporting data contain heterogeneous drug names, co-reported therapies, and incomplete treatment-context information, a formal antineoplastic-only sensitivity analysis could not be implemented reliably without introducing additional drug-name standardization assumptions. We therefore strengthened the disease-background restriction and expanded the discussion of residual confounding by indication and concomitant therapies.

**Comment 3.4. Please revise the Data Availability Statement, specify extraction quarters and download dates, and deposit the de-duplicated analytic datasets as Supporting Information.**

Response: We thank the reviewer for this important comment. We revised the Data Availability Statement to indicate that the raw FAERS and JADER data are publicly available from the U.S. Food and Drug Administration and the Pharmaceuticals and Medical Devices Agency of Japan, respectively. We specified the extraction periods and download dates: FAERS data from 2020Q1 to 2025Q4 and JADER data from 2020Q1 to 2025Q3, both downloaded on March 15, 2026. We also provided the de-duplicated event-level analytic datasets generated after applying the study-specific filtering criteria as Supporting Information (S9 Dataset and S10 Dataset), together with the derived signal-detection tables underlying the main results (S1–S8 Tables).

**Comment 3.5. The Discussion is repetitive and should be tightened.**

Response: Thank you for this suggestion. We substantially revised and reorganized the Discussion to reduce repeated descriptions of the same signal clusters. We separated the study contribution, clinical interpretation, database-specific findings, subgroup findings, time-to-onset findings, and limitations into more focused sections. We also reduced repeated figure callouts and emphasized interpretation rather than restating results.

**Comment 3.6. Please report the total number of reports and unique drugs in the reference set so readers can reproduce the contingency tables.**

Response: Thank you for this comment. We clarified that our primary disproportionality analysis was conducted as an event-level ROR analysis. The ROR calculations were based on PT event counts in the entrectinib-NSCLC target group and the NSCLC reference set, rather than on unique-drug counts. To support reproducibility, we provided the PT-specific a, b, c, and d counts in S1 Table, which are the values required to reproduce the event-level contingency tables and ROR estimates. We also revised the Methods to avoid implying that unique-drug counts were used in the signal calculation and provided the de-duplicated event-level analytic datasets as S9 Dataset and S10 Dataset for transparency.

**Reviewer #4**

**Comment 4.1. Figure 4A–B is overcrowded.**

Response: Thank you for this observation. To improve readability, we retained Figure 4 as an overview figure and added Table 3 to summarize cross-database replicated PT-level signals with event counts and ROR estimates. Complete PT-level signal lists remain available in S4 Table and S5 Table. This combination allows the main figure to function as a visual overview while providing detailed numerical results in table form.

**Comment 4.2. Please describe how the four JADER datasets were linked.**

Response: We revised the Methods to clarify that the JADER demographics, drug information, adverse reactions, and patient history datasets were linked using the unique case identifier provided in JADER to construct unified case-level records before filtering and analysis.

**Comment 4.3. The different age cutoffs between FAERS and JADER limit direct comparison.**

Response: We agree. We clarified in the Methods that FAERS age was categorized as <65 and ≥65 years, whereas JADER age was categorized as <70 and ≥70 years according to the publicly available age categories in JADER. We also added this issue to the Limitations and stated that age-stratified findings should not be used for direct cross-database comparison.

**Comment 4.4. Confounding by indication and concomitant medications should be considered.**

Response: Thank you for this important point. We expanded the Discussion and Limitations to emphasize that residual confounding by underlying disease, prior anticancer therapy, concomitant medications, comorbidities, disease progression, dose modification, and competing causes of organ dysfunction could not be fully excluded. We also clarified that some cardiac, renal, neurologic, or laboratory-related events may reflect factors other than entrectinib itself.

**Comment 4.5. Please justify using ROR as the primary signal criterion.**

Response: We revised the Methods to justify the use of ROR as the primary criterion. ROR is widely used in spontaneous reporting system analyses, is straightforward to interpret, and allows comparison with previous pharmacovigilance studies. PRR, BCPNN, and MGPS were used as complementary methods to assess robustness under different statistical assumptions.

**Reviewer #5**

**Comment 5.1. The novelty appears to be overstated.**

Response: Thank you for this helpful comment. We revised the Discussion section title and framing to “Study contribution” and clarified that the main contribution of this study is not the identification of entirely novel toxicity categories. Instead, the study contributes cross-database characterization of PT-level reporting signals, demographic subgroup patterns, and time-to-onset features in a postmarketing setting.

**Comment 5.2. Disease progression is problematic and should not be interpreted as toxicity.**

Response: We agree. We revised the Methods and Discussion to clarify that “disease progression” was retained in the initial signal-detection output for transparency but was interpreted as a disease-course-related reporting term rather than a toxicity-specific adverse event signal. We further clarified that disease progression may reflect treatment failure, tumor aggressiveness, acquired resistance, or the underlying disease course rather than a drug toxicity.

**Comment 5.3. Mechanistic discussion is speculative.**

Response: Thank you for pointing out this important issue. We agree that the previous wording could be interpreted as overly speculative. We therefore removed statements suggesting that taste disorder is an early marker of broader neurologic toxicity or that entrectinib-associated toxicity directly involves specific neurologic functions. In the revised Discussion, we used more cautious language and explicitly stated that the observed neurologic, cardiac, renal, and metabolic signals should be interpreted as pharmacovigilance signals and monitoring priorities rather than evidence of specific mechanisms or causal pathways. We also clarified that the present study did not include neuroimaging, neurophysiologic testing, molecular assays, animal experiments, cardiac imaging, or adjudicated myocarditis data.

**Comment 5.4. Some RORs and confidence intervals are very large.**

Response: We agree. We expanded the Results, Discussion, and Limitations to explicitly discuss sparse cell counts, wide confidence intervals, statistical instability, reporting sensitivity, coding differences, and random fluctuation. We now state that low-frequency signals with very large ROR estimates should be interpreted cautiously as exploratory pharmacovigilance signals rather than confirmed adverse drug reactions.

**Comment 5.5. Multiple testing may inflate false-positive signals.**

Response: We added discussion of multiplicity and false-positive signal inflation in the Methods and Limitations. We clarified that no formal multiplicity adjustment was applied because the analysis was exploratory and intended for signal detection. We also emphasized that cross-database replicated PTs are more reliable than database-specific, single-method, or sparse-count signals.

**Comment 5.6. The TTO analysis included only 203 of 520 reports.**

Response: Thank you for this important point. We revised the Methods and Results to report both the absolute number and proportion of evaluable FAERS reports included in the TTO analysis. Specifically, 203 of 520 eligible reports had complete and valid onset-time data, corresponding to 39.0%, whereas 317 reports were excluded because of missing or invalid date information. We also added discussion of potential selection bias due to missing onset dates and interpreted the TTO findings cautiously.

**Comment 5.7. The Discussion is repetitive.**

Response: We revised and tightened the Discussion to reduce repeated statements regarding neurologic, cardiac, renal, and cross-database findings. The revised Discussion emphasizes study contribution, clinical interpretation, database-specific signals, sparse-count signals, subgroup findings, TTO findings, and limitations in a more structured manner.

**Reviewer #6**

**Comment 6.1. Subgroup findings should be explicitly described as exploratory and hypothesis-generating.**

Response: We agree. We revised the Results and Discussion to clarify that age- and sex-stratified findings are exploratory and hypothesis-generating rather than definitive demographic risk differences. We also emphasized that several subgroup estimates were based on small cell counts and wide confidence intervals.

**Comment 6.2. Database-specific signals require more interpretation.**

Response: Thank you for this comment. We expanded the Discussion to explain that FAERS-specific and JADER-specific PT signals may reflect differences in database size, regional prescribing patterns, reporting sources, patient populations, coding habits, regulatory context, and random reporting variation. We also clarified that database-specific signals require further validation.

**Comment 6.3. Please report the number and proportion of reports excluded from TTO analysis.**

Response: We revised the Methods and Results to report that 203 of 520 FAERS reports had complete and valid onset-time data and were retained for TTO analysis, corresponding to 39.0% of eligible reports. The remaining 317 reports were excluded because of missing or invalid date information.

**Comment 6.4. Please define abbreviations consistently.**

Response: We reviewed the manuscript and revised abbreviation definitions at first appearance in both the Abstract and main text, including FAERS, JADER, PT, SOC, TTO, ROR, PRR, BCPNN, and MGPS.

**Comment 6.5. Please report the percentage retained for TTO analysis.**

Response: We added the percentage retained for TTO analysis in both the Methods and Results sections. Specifically, 203 of 520 eligible FAERS reports were retained, corresponding to 39.0%.

**Comment 6.6. Please distinguish pharmacovigilance signals from confirmed adverse drug reactions.**

Response: We revised the Abstract, Results, Discussion, Limitations, and Conclusion to clearly distinguish disproportional reporting signals from confirmed adverse drug reactions. We now explicitly state that the findings should be interpreted as hypothesis-generating pharmacovigilance signals rather than evidence of incidence or causality.

**Reviewer #7**

**Comment 7.1. Some abbreviations were not defined at first occurrence.**

Response: Thank you for this careful reading. We revised the Abstract and main text to define abbreviations at first use, including FAERS, JADER, NSCLC, PT, SOC, ROR, PRR, BCPNN, MGPS, and TTO.

**Comment 7.2. Please add a disclaimer regarding confounding by concomitant medications, underlying disease, and patient-related factors.**

Response: We agree. We expanded the Discussion and Limitations to state that some reported adverse events may be attributable to concomitant medications, underlying NSCLC, disease progression, prior anticancer therapy, comorbidities, or other patient-related factors rather than entrectinib itself. We also added that future cohort-based studies, registry analyses, and electronic health record studies with detailed patient-level covariates are needed to better adjust for potential confounding.

We again thank you for your rigorous and constructive comments. We believe that these revisions have substantially improved the methodological transparency, robustness, presentation, and interpretive balance of the manuscript.

Yours sincerely,

Ge Sun

On behalf of all authors
