# Supplementary material for: Safety profile of entrectinib in NSCLC: Multi-source pharmacovigilance analysis using FAERS and JADER
Source: PLoS One. 2026 Sep 15;21(9):e0358331. doi: 10.1371/journal.pone.0358331 (PMC13577382; doi:10.1371/journal.pone.0358331)
Supplement: Supplementary file 11 [file pone.0358331.s014.docx]

**Response Letter**

Dear Editor and Reviewers,

On behalf of all co-authors, we sincerely thank the editor and reviewers for their careful, constructive, and encouraging evaluation of our revised manuscript entitled “Safety profile of entrectinib in NSCLC: multi-source pharmacovigilance analysis using FAERS and JADER.” We greatly appreciate the reviewers’ recognition of the methodological rigor and substantial improvement of the manuscript. The remaining comments were highly helpful for further improving internal consistency, clarity, clinical interpretation, and presentation.

In response to the current round of review, we rechecked the citations and formatting of all Supporting Information items, standardized abbreviations throughout the manuscript, added percentages alongside absolute event counts for the cross-database replicated PT-level signals in Table 3, and reviewed all figure legends for independent interpretability. We also corrected the hyperuricaemia ROR in the Abstract, clarified the different denominators used for JADER baseline characteristics and clinical outcomes, verified the JADER “Recovered” count, and expanded the Limitations to address event-level non-independence, unavailable fusion status, and the lack of molecular comparability between the entrectinib and reference groups.

**Point-by-point responses**

**Reviewer #2**

**Comment 2.1. Ensure every supplementary table (S1–S10) is cited sequentially in the Results.**

Response: Thank you for this careful suggestion. We rechecked all Supporting Information citations and ensured that the items are introduced in numerical order at their first relevant locations. S1 and S2 Tables are cited in the Methods because they describe the event-level contingency table and the disproportionality algorithms and signal criteria; S3–S8 Tables are cited sequentially in the Results; and S9 and S10 are de-duplicated analytic datasets and are cited in the Data Availability Statement. We retained the designations “S9 Dataset” and “S10 Dataset” because these items are datasets rather than supplementary tables.

**Comment 2.2. Verify consistent formatting of abbreviations throughout the manuscript.**

Response: Thank you for this helpful comment. We conducted a comprehensive review of abbreviations in the Abstract, main text, tables, and figure legends. We standardized the use and formatting of FAERS, JADER, NSCLC, AE, SOC, PT, ROR, PRR, BCPNN, MGPS, EBGM, TTO, CI, and IQR.

**Comment 2.3. Consider reporting percentages alongside absolute event counts whenever PT-level signals are summarized.**

Response: Thank you for this useful recommendation. To improve interpretability without overloading the main text with additional numerical detail, we added corresponding percentages alongside absolute event counts for all 23 cross-database replicated PT-level signals in Table 3. The column headings were revised to “n (%).” Percentages were calculated using the total number of PT-level adverse event records in the corresponding database as the denominator (FAERS, n = 1,573; JADER, n = 393). We also clarified in the table note and Figure 4 legend that these percentages describe the distribution of reported PT events and should not be interpreted as incidence rates or patient-level risks.

**Comment 2.4. Review figure legends to ensure they can be interpreted independently of the main text.**

Response: We agree and carefully reviewed the legends for Figures 1–6. We expanded the legends where additional clarification was needed, particularly for Figures 3–5. The revised legends now describe the contents of each panel, the relevant analysis denominators, the meaning of n (%), the ROR point estimates and 95% confidence intervals, the reference line, truncated confidence intervals, complementary heatmap metrics, subgroup comparison directions, and all relevant abbreviations. These revisions allow the figures to be interpreted without relying on the main text.

**Reviewer #3**

**Comment 3.1. The Abstract reports hyperuricaemia as 6.04/59.63, whereas Table 3 provides an FAERS ROR of 7.36 (95% CI 2.30–23.55).**

Response: Thank you for identifying this inconsistency. We corrected the FAERS ROR for hyperuricaemia in the Abstract from 6.04 to 7.36. The revised Abstract now reports hyperuricaemia as 7.36/59.63 for FAERS/JADER, consistent with Table 3.

**Comment 3.2. The denominator used for the JADER outcome percentages in Table 2 differs from the denominator stated in the table note. Please harmonize the tables, correct the note, and verify the JADER “Recovered” count of 147.**

Response: Thank you for this careful observation. We rechecked the underlying JADER data and confirmed that sex, body weight, age, and reporter type were summarized at the unique-patient level using 254 patients, whereas clinical outcomes were summarized at the PT-event level using 393 PT-level adverse event records. We therefore revised the Table 2 note to explicitly distinguish these denominators. Table 1 remains based on 520 eligible FAERS patients. We also rechecked the source data for the JADER “Recovered” category and confirmed that the count of 147 is correct. Its identical value to the number of female patients is coincidental and does not result from a duplicated or incorrectly referenced source cell.

**Comment 3.3. Please acknowledge event-level non-independence in the Limitations, state that fusion status could not be verified and that the reference population was not molecularly comparable, and consider softening the ROS1 framing in the Abstract.**

Response: Thank you for this important comment. We revised the Limitations to explicitly state that a single patient or case report could contribute multiple PT events; therefore, event-level observations were not fully independent, and within-report clustering may have influenced the disproportionality estimates. We also added that ROS1 fusion status could not be verified in FAERS or JADER and that the NSCLC reference population could not be considered molecularly comparable to the entrectinib group. In addition, the Abstract now explicitly states that molecular fusion status was not available for verification, thereby avoiding the implication that all included NSCLC reports were molecularly confirmed ROS1-positive cases.

We again thank the editor and reviewers for their rigorous and constructive comments. We believe that these revisions have further improved the internal consistency, transparency, presentation, and interpretive balance of the manuscript.

Yours sincerely,

Ge Sun

On behalf of all authors
