# Supplementary material for: Safety profile of entrectinib in NSCLC: Multi-source pharmacovigilance analysis using FAERS and JADER
Source: PLoS One. 2026 Sep 15;21(9):e0358331. doi: 10.1371/journal.pone.0358331 (PMC13577382; doi:10.1371/journal.pone.0358331)
Supplement: Peer review history

# Peer review: Decision Letter 0

**DOI:** 10.1371/journal.pone.0358331.r001

**Authors:** Guichuan Lai

28 Jun 2026

Dear Dr. Sun,

Thank you for submitting your manuscript to PLOS ONE. After careful consideration, we feel that it has merit but does not fully meet PLOS ONE’s publication criteria as it currently stands. Therefore, we invite you to submit a revised version of the manuscript that addresses the points raised during the review process.

Please submit your revised manuscript by Aug 27 2026 11:59PM. If you will need more time than this to complete your revisions, please reply to this message or contact the journal office at plosone@plos.org. When you're ready to submit your revision, log on to https://www.editorialmanager.com/pone/ and select the 'Submissions Needing Revision' folder to locate your manuscript file.

- A letter that responds to each point raised by the academic editor and reviewer(s). You should upload this letter as a separate file labeled 'Response to Reviewers'.
- A marked-up copy of your manuscript that highlights changes made to the original version. You should upload this as a separate file labeled 'Revised Manuscript with Track Changes'.
- An unmarked version of your revised paper without tracked changes. You should upload this as a separate file labeled 'Manuscript'.

If you would like to make changes to your financial disclosure, please include your updated statement in your cover letter. Guidelines for resubmitting your figure files are available below the reviewer comments at the end of this letter.

If applicable, we recommend that you deposit your laboratory protocols in protocols.io to enhance the reproducibility of your results. Protocols.io assigns your protocol its own identifier (DOI) so that it can be cited independently in the future. For instructions see: https://journals.plos.org/plosone/s/submission-guidelines#loc-laboratory-protocols. Additionally, PLOS ONE offers an option for publishing peer-reviewed Lab Protocol articles, which describe protocols hosted on protocols.io. Read more information on sharing protocols at https://plos.org/protocols?utm_medium=editorial-email&utm_source=authorletters&utm_campaign=protocols.

As the corresponding author, your ORCID iD is verified in the submission system and will appear in the published article. PLOS supports the use of ORCID, and we encourage all coauthors to register for an ORCID iD and use it as well. Please encourage your coauthors to verify their ORCID iD within the submission system before final acceptance, as unverified ORCID iDs will not appear in the published article. *Only*  the individual author can complete the verification step; PLOS staff *cannot*  verify ORCID iDs on behalf of authors.

We look forward to receiving your revised manuscript.

Kind regards,

Guichuan Lai

Academic Editor

PLOS One

**Journal Requirements:**

When submitting your revision, we need you to address these additional requirements.

1. Please ensure that your manuscript meets PLOS ONE's style requirements, including those for file naming. The PLOS ONE style templates can be found at

https://journals.plos.org/plosone/s/file?id=wjVg/PLOSOne_formatting_sample_main_body.pdf and

https://journals.plos.org/plosone/s/file?id=ba62/PLOSOne_formatting_sample_title_authors_affiliations.pdf

2. Thank you for stating the following financial disclosure:

“G.S. received funding from the cultivating scientific research project of the Second Hospital of Dalian Medical University (grant number YJ20250033).”

Please state what role the funders took in the study.  If the funders had no role, please state: "The funders had no role in study design, data collection and analysis, decision to publish, or preparation of the manuscript."

If this statement is not correct you must amend it as needed.

Please include this amended Role of Funder statement in your cover letter; we will change the online submission form on your behalf.

3. Please note that funding information should not appear in any section or other areas of your manuscript. We will only publish funding information present in the Funding Statement section of the online submission form. Please remove any funding-related text from the manuscript.

4. In the online submission form you indicate that your data is not available for proprietary reasons and have provided a contact point for accessing this data. Please note that your current contact point is a co-author on this manuscript. According to our Data Policy, the contact point must not be an author on the manuscript and must be an institutional contact, ideally not an individual. Please revise your data statement to a non-author institutional point of contact, such as a data access or ethics committee, and send this to us via return email. Please also include contact information for the third party organization, and please include the full citation of where the data can be found.

5. Your ethics statement should only appear in the Methods section of your manuscript. If your ethics statement is written in any section besides the Methods, please move it to the Methods section and delete it from any other section. Please ensure that your ethics statement is included in your manuscript, as the ethics statement entered into the online submission form will not be published alongside your manuscript.

6. Please remove your figures from within your manuscript file, leaving only the individual TIFF/EPS image files, uploaded separately. These will be automatically included in the reviewers’ PDF.

7. Please include captions for your Supporting Information files at the end of your manuscript, and update any in-text citations to match accordingly. Please see our Supporting Information guidelines for more information: http://journals.plos.org/plosone/s/supporting-information.

8. If the reviewer comments include a recommendation to cite specific previously published works, please review and evaluate these publications to determine whether they are relevant and should be cited. There is no requirement to cite these works unless the editor has indicated otherwise.

[Note: HTML markup is below. Please do not edit.]

Reviewers' comments:

Reviewer's Responses to Questions

**Comments to the Author**

1. Is the manuscript technically sound, and do the data support the conclusions?

Reviewer #1: Yes

Reviewer #2: Yes

Reviewer #3: Partly

Reviewer #4: Yes

Reviewer #5: Yes

Reviewer #6: Yes

Reviewer #7: Yes

**********

2. Has the statistical analysis been performed appropriately and rigorously?

Reviewer #1: Yes

Reviewer #2: Yes

Reviewer #3: Yes

Reviewer #4: Yes

Reviewer #5: Yes

Reviewer #6: Yes

Reviewer #7: Yes

**********

3. Have the authors made all data underlying the findings in their manuscript fully available?

The PLOS Data policy

Reviewer #1: Yes

Reviewer #2: Yes

Reviewer #3: Yes

Reviewer #4: No

Reviewer #5: Yes

Reviewer #6: No

Reviewer #7: Yes

**********

4. Is the manuscript presented in an intelligible fashion and written in standard English?

Reviewer #1: Yes

Reviewer #2: Yes

Reviewer #3: Yes

Reviewer #4: Yes

Reviewer #5: Yes

Reviewer #6: Yes

Reviewer #7: Yes

**********

Reviewer #1: Dear Authors,

I commend your excellent work on this manuscript and appreciate the opportunity to review it. It is a valuable contribution to the real-world safety profile of entrectinib use for NSCLC. I have a few suggestions to improve the quality of your work:

1. Consider including more quantitative results (e.g., specific RORs for key PTs) in your abstract to highlight the magnitude of the main signals.

2. Add a summary table of the most significant PT-level signals shared across both databases; about ten or as preferred.

Well done!

Reviewer #2: This manuscript evaluates the real-world safety profile of entrectinib using two major spontaneous reporting systems and identifies reproducible neurologic, cardiac, and renal adverse-event signals. The dual-database design and inclusion of TTO analyses provide clinically relevant information and enhance the study's value.

However, the conclusions currently overstate the strength of the evidence generated by disproportionality analyses. The manuscript would benefit from additional sensitivity analyses, a more balanced discussion of confounding and reporting bias, clearer justification of methodological choices, and deeper clinical interpretation of the identified signals.

1. Clinical interpretation is superficial

Several interesting signals are identified:

myocarditis

cardiomyopathy

cardiac failure

cognitive disorder

ataxia

renal impairment

taste disorder

However, the manuscript provides relatively limited mechanistic discussion.

For example:

Why would entrectinib preferentially produce neurologic toxicity?

What kinase inhibition pathways could explain myocarditis?

Are renal signals direct drug effects or consequences of dehydration, tumor lysis, or disease progression?

A deeper biological discussion would substantially strengthen the paper.

2. Database-specific signals deserve more attention

The manuscript reports:

58 PTs unique to FAERS

only 8 unique to JADER

This asymmetry likely reflects differences in:

reporting systems,

database size,

pharmacovigilance practices,

regional prescribing.

3. Annual reporting trends may reflect reporting bias

The manuscript interprets increasing report numbers as post-marketing surveillance trends.

However, increasing reports could equally result from:

expanding drug utilization,

greater clinician awareness,

regulatory communications,

publicity.

4. Discussion

The Discussion appropriately emphasizes the predominance of neurologic, cardiac, and renal toxicities.

The wording should consistently emphasize that disproportionality analyses generate hypotheses rather than establish causality.

Reviewer #3: The study applies appropriate pharmacovigilance methods (ROR, PRR, BCPNN, MGPS). The topic is clinically useful, the cross-database design is a genuine strength, and the conclusions are, for the most part, appropriately framed as signals rather than causal effects. The following points should be addressed.

1. Please provide the meaning of the abbreviations used in the study the first time you use them.

2. Multiple comparisons. A large number of PTs are tested across four algorithms with no adjustment for multiplicity, which inflates false-positive signals. Please state the issue explicitly and consider focusing primary interpretation on signals concordant across both databases and all four methods. The cross-database replication (23 shared PTs) is the most defensible part of the analysis and should be foregrounded over single-database, single-method hits.

3. The comparator group is not defined. The methods do not state the reference set used for disproportionality (whole database vs. all other drugs and whether any restriction was applied). Because cases were restricted to the NSCLC indication while presumably being compared against the full database, the indication-specific RORs may be confounded by both the indication and notoriety. Please define the comparator and add a sensitivity analysis that restricts it to other antineoplastic agents.

4. On data availability. Your statement says data are "available from the corresponding author on reasonable request," which is inconsistent with the fact that the underlying data are public and with PLOS policy. Please revise to point to the public FAERS/PMDA sources. Furthermore, please specify the exact extraction quarters and download dates and deposit the de-duplicated analytic datasets as supporting information.

5. The Discussion is repetitive, restating the same signal clusters with frequent figure callouts. Tightening would improve readability without loss of content.

6. Please report the total number of reports and unique drugs in the reference set so readers can reproduce the contingency tables.

Thank you.

Reviewer #4: This manuscript presents a multi-source pharmacovigilance analysis of the safety profile of entrectinib in non-small cell lung cancer (NSCLC). By leveraging data from the FAERS and JADER databases, the authors employ four disproportionality methods to identify adverse event (AE) signals, conduct age- and sex-stratified subgroup analyses, and assess time-to-onset patterns. The study's primary contribution is its cross-database validation of safety signals, which strengthens the evidence for a core set of toxicities—notably neurologic, cardiac, and renal—that appear early in treatment. The work is methodologically sound and clinically relevant, offering a valuable real-world complement to clinical trial data. However, several fundamental methodological and reporting limitations preclude acceptance for publication.

1. While supplementary tables are comprehensive, the main figures (particularly Figure 4A-B) are overcrowded, making it difficult to read individual PTs and their corresponding ROR values. A more focused or interactive figure would improve clarity.

2. The methods state that JADER “consists of four datasets” (DEMO, DRUG, REAC, HIST) but do not explicitly describe how these datasets are linked (e.g., using a unique ID) to create a unified patient-level record. For FAERS, the linkage using CASEID is described, but for JADER, it is omitted.

3. The manuscript uses different age cutoffs for FAERS (<65 vs. ≥65) and JADER (<70 vs. ≥70). While the authors acknowledge the reason for JADER's broad categories, this inconsistency makes direct comparison of age-related findings between databases difficult and is a notable limitation.

4. The study acknowledges the limitations of spontaneous reporting systems but does not quantify the potential impact of confounding by indication or concomitant medications. For instance, NSCLC patients may have many comorbidities and take multiple drugs, which could influence the reporting of cardiac and renal events. This could be explored by analyzing reports where entrectinib is the sole suspect drug vs. not.

5. While ROR is a widely used method, the authors also employed other methods. They should provide a justification for using ROR as the primary definition of a positive signal, especially since the overlap between methods is not perfect (as shown in Figure 3), and some PTs are only detected by one method.

Reviewer #5: The research has been conducted with rigorous methodology. The analysis of entrectinib across both pharmacovigilance databases, FAERS and JADER, has been well executed, including four disproportionality analyses: ROR, PRR, BCPNN, and MGPS. The multi-layered analysis of the drug at both SOC and PT levels, age-subgroup analysis, sex-subgroup analysis, and time-to-onset analysis have strengthened this study. However, several important improvements are required before the research is published. Please look for the attached file.

Reviewer #6: This manuscript presents a dual-database pharmacovigilance analysis evaluating the real-world safety profile of entrectinib in patients with NSCLC using FAERS and JADER. By integrating signal detection across two independent spontaneous reporting systems together with subgroup and time-to-onset analyses, the study provides a more comprehensive characterization of entrectinib-associated adverse events than previous single-database reports. Overall, the study is well designed and clinically relevant.

Minor Comments:

1. Results Sections 3.3–3.4; Discussion. For example, the female predominance of ataxia is based on only 16 versus 2 reports, resulting in a very wide confidence interval. Similar issues are present for several age-specific signals. Although the limitations section acknowledges this issue, the Results and Discussion could more explicitly emphasize that these subgroup findings are exploratory and hypothesis-generating rather than definitive demographic differences.

2. Results Section 3.2; Discussion. The manuscript appropriately reports PTs that were shared across FAERS and JADER as well as signals unique to each database. However, it remains unclear whether database-specific signals are more likely attributable to regional prescribing patterns, reporting practices, differences in patient populations, or random reporting variation. Expanding this discussion would help readers better interpret the clinical significance of these unique findings.

3. The manuscript states that only reports with complete and valid dates were included, but the number and proportion of reports excluded because of missing or implausible dates are not provided. Reporting these numbers would improve transparency and allow readers to assess the representativeness of the TTO analysis.

4. Please ensure that all abbreviations (e.g., PT, SOC, TTO, MGPS, BCPNN) are consistently defined at first appearance in both the Abstract and the main text.

5. Consider reporting the percentage of reports retained for the TTO analysis in addition to the absolute number of eligible reports.

6. In the Discussion, the authors may consider explicitly distinguishing “pharmacovigilance signals” from “confirmed adverse drug reactions” to avoid any implication of causality.

Reviewer #7: Dear Authors,

One of the major strengths of this study is the cross-validation performed using two independent data sources. The descriptive analysis of adverse events was well presented, and I appreciated the clear flow of the key findings. I have two suggestions that could further improve the manuscript:

- I noticed consistent use of abbreviations that were not defined when first introduced, particularly in line 65, line 102 (e.g., PT events), and line 170. Please ensure that all abbreviations are spelled out at their first occurrence.

- I understand that you were careful not to infer causal associations from the disproportionality analysis. However, I recommend adding a brief disclaimer emphasizing that some reported adverse events may be attributable to concomitant medications, the underlying disease, or other patient-related factors rather than entrectinib itself. It would also be helpful to recommend that future studies adjust for relevant clinical and demographic characteristics to better account for potential confounding.

Overall, I believe this is a relevant and timely study that could influence how clinicians view the use of entrectinib in patients with NSCLC.

**********

what does this mean?). If published, this will include your full peer review and any attached files.

If you choose “no”, your identity will remain anonymous but your review may still be made public.

**Do you want your identity to be public for this peer review?**  For information about this choice, including consent withdrawal, please see our Privacy Policy

Reviewer #1: **Yes:** Dr. Oluwafolayemi Doyeni

Reviewer #2: **Yes:** Juan Carlos Alvarado MD, MSc.

Reviewer #3: No

Reviewer #4: No

Reviewer #5: **Yes:** SABREENA ALAM

Reviewer #6: **Yes:** Yuhang Liu

Reviewer #7: **Yes:** Adebowale Sylvester Adeyemi

**********

[NOTE: If reviewer comments were submitted as an attachment file, they will be attached to this email and accessible via the submission site. Please log into your account, locate the manuscript record, and check for the action link "View Attachments". If this link does not appear, there are no attachment files.]

To ensure your figures meet our technical requirements, please review our figure guidelines: https://journals.plos.org/plosone/s/figures

You may also use PLOS’s free figure tool, NAAS, to help you prepare publication quality figures: https://journals.plos.org/plosone/s/figures#loc-tools-for-figure-preparation.

NAAS will assess whether your figures meet our technical requirements by comparing each figure against our figure specifications.

Attachment Submitted filename: review for plos one- FAERS and JADER pharmacovigilance.docx

# Author comment: Author response to Decision Letter 1

**DOI:** 10.1371/journal.pone.0358331.r002

10 Jul 2026

Response Letter

Dear Editor and Reviewers,

On behalf of all co-authors, we sincerely thank the editor and reviewers for their thoughtful, detailed, and constructive comments on our manuscript entitled “Safety profile of entrectinib in NSCLC: multi-source pharmacovigilance analysis using FAERS and JADER.” These comments were very helpful for improving the methodological clarity, presentation of pharmacovigilance findings, interpretation of disproportionality signals, and alignment with PLOS ONE reporting requirements.

In the revised manuscript, we clarified the data sources and reference set, revised the Abstract, expanded the interpretation of neurologic, cardiac, and renal reporting signals, emphasized the hypothesis-generating nature of disproportionality analyses, added a summary table of cross-database replicated PT-level signals, revised the Data Availability Statement, and provided de-duplicated event-level analytic datasets as Supporting Information. We also revised the figure captions, Supporting Information captions, and file names according to PLOS ONE requirements.

Point-by-point responses

Reviewer #1

Comment 1.1. Consider including more quantitative results, such as specific RORs for key PTs, in the abstract.

Response: Thank you for this helpful suggestion. We revised the Abstract to include quantitative ROR estimates for the major system organ class-level and representative shared preferred-term-level signals. Specifically, we added ROR values for nervous system disorders, cardiac disorders, renal and urinary disorders, and representative shared PT signals such as dizziness, renal impairment, blood creatinine increased, cardiac failure, cognitive disorder, ataxia, syncope, myocarditis, electrocardiogram QT prolonged, and hyperuricaemia. We also revised the wording to emphasize that these findings represent pharmacovigilance signals rather than incidence or causal effects.

Comment 1.2. Add a summary table of the most significant PT-level signals shared across both databases.

Response: Thank you for this recommendation. We added Table 3, entitled “Cross-database replicated PT-level signals of entrectinib-associated adverse event reports in FAERS and JADER.” This table summarizes all 23 PT-level signals shared between FAERS and JADER, including event counts and ROR estimates with 95% confidence intervals in both databases. We believe this table improves readability and directly highlights the most reproducible component of the analysis.

Reviewer #2

Comment 2.1. The clinical interpretation is superficial, and deeper interpretation of neurologic, cardiac, renal, and taste-related signals is needed.

Response: Thank you for this important comment. We agree that the clinical interpretation of the major signal clusters should be strengthened, while avoiding overstatement of causality. We therefore revised the Discussion to provide a more detailed but cautious interpretation of the neurologic, cardiac, renal, and metabolic signals. Specifically, we discussed that entrectinib, as a TRK/ROS1/ALK inhibitor with intracranial antitumor activity, provides a biologically plausible context for the repeated reporting of neurologic events such as dizziness, cognitive disorder, ataxia, gait disturbance, syncope, and taste disorder. We also expanded the interpretation of cardiac signals, including cardiac failure, cardiomyopathy, myocarditis, arrhythmia, electrocardiogram QT prolonged, troponin I increased, and left ventricular dysfunction, and discussed possible overlapping explanations such as kinase-inhibition-related effects, electrophysiologic vulnerability, baseline cardiovascular disease, prior anticancer therapy, and concomitant QT-prolonging medications. In addition, we clarified that renal impairment, renal disorder, blood creatinine increased, and hyperuricaemia may reflect multiple pathways, including dehydration, hemodynamic instability, concomitant nephrotoxic drugs, tumor-related clinical deterioration, tumor lysis-related metabolic disturbance, or progression of baseline renal dysfunction. At the same time, we explicitly stated that FAERS and JADER do not provide sufficient clinical, imaging, laboratory, electrocardiographic, or mechanistic information to establish specific causal pathways. Therefore, these findings were interpreted as pharmacovigilance signals and monitoring priorities rather than mechanistically confirmed toxicities.

Comment 2.2. Database-specific signals deserve more attention.

Response: We agree. We expanded the interpretation of database-specific PT signals. We now discuss that the larger number of FAERS-specific PTs compared with JADER-specific PTs may reflect differences in database size, reporting sources, regional prescribing patterns, clinical practice, regulatory context, coding habits, reporting sensitivity, and random variation, particularly for rare events. We also emphasize that database-specific signals should be interpreted as exploratory findings requiring further validation.

Comment 2.3. Annual reporting trends may reflect reporting bias.

Response: Thank you for highlighting this issue. We revised the Results and Discussion to clarify that annual report counts describe reporting activity rather than true changes in the incidence of adverse events. We added that reporting trends may be influenced by drug utilization, clinician awareness, regulatory communications, media attention, public concern, and pharmacovigilance practices.

Comment 2.4. The wording should consistently emphasize that disproportionality analyses generate hypotheses rather than establish causality.

Response: We agree. Throughout the revised Abstract, Results, Discussion, Limitations, and Conclusion, we replaced causal or definitive language with wording such as “disproportional reporting signals,” “hypothesis-generating,” and “pharmacovigilance signals.” We explicitly state that the findings should not be interpreted as evidence of incidence, absolute risk, or confirmed causal adverse drug reactions.

Reviewer #3

Comment 3.1. Please define abbreviations at first use.

Response: Thank you for this comment. We reviewed the manuscript and revised abbreviation definitions at first appearance in both the Abstract and main text. We defined FAERS, JADER, NSCLC, SOC, PT, ROR, PRR, BCPNN, MGPS, TTO, and other relevant abbreviations where they first appear.

Comment 3.2. Multiple comparisons may inflate false-positive signals; please emphasize cross-database replicated signals over single-database or single-method signals.

Response: We agree. We added a statement in the Methods explaining that no formal multiplicity adjustment was applied because disproportionality analysis is intended for pharmacovigilance signal detection rather than confirmatory hypothesis testing. We also revised the Results, Discussion, and Limitations to emphasize the 23 cross-database replicated PTs as the most reproducible component of the analysis, while interpreting database-specific, single-method, and sparse-count signals as exploratory and hypothesis-generating.

Comment 3.3. The comparator group is not defined, and a sensitivity analysis restricted to other antineoplastic agents was suggested.

Response: Thank you for this important methodological point. We clarified that disproportionality analyses were conducted within the NSCLC reporting background rather than against the entire FAERS or JADER database. The target group consisted of reports in which entrectinib was recorded as the primary suspect drug for NSCLC-related indications. The reference set consisted of NSCLC-related reports involving all other drugs after excluding entrectinib-related reports. Thus, the ROR estimates reflect disproportionate reporting relative to other drug reports within the same disease-reporting background. Because the publicly available spontaneous reporting data contain heterogeneous drug names, co-reported therapies, and incomplete treatment-context information, a formal antineoplastic-only sensitivity analysis could not be implemented reliably without introducing additional drug-name standardization assumptions. We therefore strengthened the disease-background restriction and expanded the discussion of residual confounding by indication and concomitant therapies.

Comment 3.4. Please revise the Data Availability Statement, specify extraction quarters and download dates, and deposit the de-duplicated analytic datasets as Supporting Information.

Response: We thank the reviewer for this important comment. We revised the Data Availability Statement to indicate that the raw FAERS and JADER data are publicly available from the U.S. Food and Drug Administration and the Pharmaceuticals and Medical Devices Agency of Japan, respectively. We specified the extraction periods and download dates: FAERS data from 2020Q1 to 2025Q4 and JADER data from 2020Q1 to 2025Q3, both downloaded on March 15, 2026. We also provided the de-duplicated event-level analytic datasets generated after applying the study-specific filtering criteria as Supporting Information (S9 Dataset and S10 Dataset), together with the derived signal-detection tables underlying the main results (S1–S8 Tables).

Comment 3.5. The Discussion is repetitive and should be tightened.

Response: Thank you for this suggestion. We substantially revised and reorganized the Discussion to reduce repeated descriptions of the same signal clusters. We separated the study contribution, clinical interpretation, database-specific findings, subgroup findings, time-to-onset findings, and limitations into more focused sections. We also reduced repeated figure callouts and emphasized interpretation rather than restating results.

Comment 3.6. Please report the total number of reports and unique drugs in the reference set so readers can reproduce the contingency tables.

Response: Thank you for this comment. We clarified that our primary disproportionality analysis was conducted as an event-level ROR analysis. The ROR calculations were based on PT event counts in the entrectinib-NSCLC target group and the NSCLC reference set, rather than on unique-drug counts. To support reproducibility, we provided the PT-specific a, b, c, and d counts in S1 Table, which are the values required to reproduce the event-level contingency tables and ROR estimates. We also revised the Methods to avoid implying that unique-drug counts were used in the signal calculation and provided the de-duplicated event-level analytic datasets as S9 Dataset and S10 Dataset for transparency.

Reviewer #4

Comment 4.1. Figure 4A–B is overcrowded.

Response: Thank you for this observation. To improve readability, we retained Figure 4 as an overview figure and added Table 3 to summarize cross-database replicated PT-level signals with event counts and ROR estimates. Complete PT-level signal lists remain available in S4 Table and S5 Table. This combination allows the main figure to function as a visual overview while providing detailed numerical results in table form.

Comment 4.2. Please describe how the four JADER datasets were linked.

Response: We revised the Methods to clarify that the JADER demographics, drug information, adverse reactions, and patient history datasets were linked using the unique case identifier provided in JADER to construct unified case-level records before filtering and analysis.

Comment 4.3. The different age cutoffs between FAERS and JADER limit direct comparison.

Response: We agree. We clarified in the Methods that FAERS age was categorized as <65 and ≥65 years, whereas JADER age was categorized as <70 and ≥70 years according to the publicly available age categories in JADER. We also added this issue to the Limitations and stated that age-stratified findings should not be used for direct cross-database comparison.

Comment 4.4. Confounding by indication and concomitant medications should be considered.

Response: Thank you for this important point. We expanded the Discussion and Limitations to emphasize that residual confounding by underlying disease, prior anticancer therapy, concomitant medications, comorbidities, disease progression, dose modification, and competing causes of organ dysfunction could not be fully excluded. We also clarified that some cardiac, renal, neurologic, or laboratory-related events may reflect factors other than entrectinib itself.

Comment 4.5. Please justify using ROR as the primary signal criterion.

Response: We revised the Methods to justify the use of ROR as the primary criterion. ROR is widely used in spontaneous reporting system analyses, is straightforward to interpret, and allows comparison with previous pharmacovigilance studies. PRR, BCPNN, and MGPS were used as complementary methods to assess robustness under different statistical assumptions.

Reviewer #5

Comment 5.1. The novelty appears to be overstated.

Response: Thank you for this helpful comment. We revised the Discussion section title and framing to “Study contribution” and clarified that the main contribution of this study is not the identification of entirely novel toxicity categories. Instead, the study contributes cross-database characterization of PT-level reporting signals, demographic subgroup patterns, and time-to-onset features in a postmarketing setting.

Comment 5.2. Disease progression is problematic and should not be interpreted as toxicity.

Response: We agree. We revised the Methods and Discussion to clarify that “disease progression” was retained in the initial signal-detection output for transparency but was interpreted as a disease-course-related reporting term rather than a toxicity-specific adverse event signal. We further clarified that disease progression may reflect treatment failure, tumor aggressiveness, acquired resistance, or the underlying disease course rather than a drug toxicity.

Comment 5.3. Mechanistic discussion is speculative.

Response: Thank you for pointing out this important issue. We agree that the previous wording could be interpreted as overly speculative. We therefore removed statements suggesting that taste disorder is an early marker of broader neurologic toxicity or that entrectinib-associated toxicity directly involves specific neurologic functions. In the revised Discussion, we used more cautious language and explicitly stated that the observed neurologic, cardiac, renal, and metabolic signals should be interpreted as pharmacovigilance signals and monitoring priorities rather than evidence of specific mechanisms or causal pathways. We also clarified that the present study did not include neuroimaging, neurophysiologic testing, molecular assays, animal experiments, cardiac imaging, or adjudicated myocarditis data.

Comment 5.4. Some RORs and confidence intervals are very large.

Response: We agree. We expanded the Results, Discussion, and Limitations to explicitly discuss sparse cell counts, wide confidence intervals, statistical instability, reporting sensitivity, coding differences, and random fluctuation. We now state that low-frequency signals with very large ROR estimates should be interpreted cautiously as exploratory pharmacovigilance signals rather than confirmed adverse drug reactions.

Comment 5.5. Multiple testing may inflate false-positive signals.

Response: We added discussion of multiplicity and false-positive signal inflation in the Methods and Limitations. We clarified that no formal multiplicity adjustment was applied because the analysis was exploratory and intended for signal detection. We also emphasized that cross-database replicated PTs are more reliable than database-specific, single-method, or sparse-count signals.

Comment 5.6. The TTO analysis included only 203 of 520 reports.

Response: Thank you for this important point. We revised the Methods and Results to report both the absolute number and proportion of evaluable FAERS reports included in the TTO analysis. Specifically, 203 of 520 eligible reports had complete and valid onset-time data, corresponding to 39.0%, whereas 317 reports were excluded because of missing or invalid date information. We also added discussion of potential selection bias due to missing onset dates and interpreted the TTO findings cautiously.

Comment 5.7. The Discussion is repetitive.

Response: We revised and tightened the Discussion to reduce repeated statements re

Attachment Submitted filename: Response_to_Reviewers.docx

# Peer review: Decision Letter 1

**DOI:** 10.1371/journal.pone.0358331.r003

**Authors:** Guichuan Lai

5 Aug 2026

Dear Dr. Sun,

Thank you for submitting your manuscript to PLOS ONE. After careful consideration, we feel that it has merit but does not fully meet PLOS ONE’s publication criteria as it currently stands. Therefore, we invite you to submit a revised version of the manuscript that addresses the points raised during the review process.

Please submit your revised manuscript by Oct 04 2026 11:59PM. If you will need more time than this to complete your revisions, please reply to this message or contact the journal office at plosone@plos.org. When you're ready to submit your revision, log on to https://www.editorialmanager.com/pone/ and select the 'Submissions Needing Revision' folder to locate your manuscript file.

- A letter that responds to each point raised by the academic editor and reviewer(s). You should upload this letter as a separate file labeled 'Response to Reviewers'.
- A marked-up copy of your manuscript that highlights changes made to the original version. You should upload this as a separate file labeled 'Revised Manuscript with Track Changes'.
- An unmarked version of your revised paper without tracked changes. You should upload this as a separate file labeled 'Manuscript'.

If you would like to make changes to your financial disclosure, please include your updated statement in your cover letter. Guidelines for resubmitting your figure files are available below the reviewer comments at the end of this letter.

If applicable, we recommend that you deposit your laboratory protocols in protocols.io to enhance the reproducibility of your results. Protocols.io assigns your protocol its own identifier (DOI) so that it can be cited independently in the future. For instructions see: https://journals.plos.org/plosone/s/submission-guidelines#loc-laboratory-protocols. Additionally, PLOS ONE offers an option for publishing peer-reviewed Lab Protocol articles, which describe protocols hosted on protocols.io. Read more information on sharing protocols at https://plos.org/protocols?utm_medium=editorial-email&utm_source=authorletters&utm_campaign=protocols.

As the corresponding author, your ORCID iD is verified in the submission system and will appear in the published article. PLOS supports the use of ORCID, and we encourage all coauthors to register for an ORCID iD and use it as well. Please encourage your coauthors to verify their ORCID iD within the submission system before final acceptance, as unverified ORCID iDs will not appear in the published article. *Only*  the individual author can complete the verification step; PLOS staff *cannot*  verify ORCID iDs on behalf of authors.

We look forward to receiving your revised manuscript.

Kind regards,

Guichuan Lai

Academic Editor

PLOS One

Journal Requirements:

If the reviewer comments include a recommendation to cite specific previously published works, please review and evaluate these publications to determine whether they are relevant and should be cited. There is no requirement to cite these works unless the editor has indicated otherwise.

[Note: HTML markup is below. Please do not edit.]

Reviewers' comments:

Reviewer's Responses to Questions

**Comments to the Author**

Reviewer #1: All comments have been addressed

Reviewer #2: All comments have been addressed

Reviewer #3: (No Response)

Reviewer #5: All comments have been addressed

Reviewer #6: All comments have been addressed

**********

2. Is the manuscript technically sound, and do the data support the conclusions?

Reviewer #1: (No Response)

Reviewer #2: Yes

Reviewer #3: Partly

Reviewer #5: Yes

Reviewer #6: Yes

**********

3. Has the statistical analysis been performed appropriately and rigorously?

Reviewer #1: (No Response)

Reviewer #2: Yes

Reviewer #3: Yes

Reviewer #5: Yes

Reviewer #6: Yes

**********

4. Have the authors made all data underlying the findings in their manuscript fully available?

The PLOS Data policy

Reviewer #1: (No Response)

Reviewer #2: Yes

Reviewer #3: Yes

Reviewer #5: Yes

Reviewer #6: Yes

**********

5. Is the manuscript presented in an intelligible fashion and written in standard English?

Reviewer #1: (No Response)

Reviewer #2: Yes

Reviewer #3: Yes

Reviewer #5: Yes

Reviewer #6: Yes

**********

Reviewer #1: (No Response)

Reviewer #2: the manuscript represents a rigorous and clinically relevant pharmacovigilance study evaluating the real-world safety profile of entrectinib using two independent international adverse event reporting databases. The methodology is appropriate, the statistical analyses are robust, and the authors appropriately avoid causal interpretations of disproportionality signals.

The remaining issues are limited to improving clarity, clinical interpretation, and presentation rather than addressing substantive methodological concerns.

Minor revision:

-Ensure every supplementary table (S1–S10) is cited sequentially in the Results.

-Verify consistent formatting of abbreviations throughout the manuscript.

-Consider reporting percentages alongside absolute event counts whenever PT-level signals are summarized.

-Review figure legends to ensure they can be interpreted independently of the main text.

Reviewer #3: Excellent job putting this manuscript together and responding to the reviewer comments constructively and in detail. Your manuscript has substantially improved. The comparator definition is now explicit; causal language has been replaced with disproportional-reporting language throughout; Table 3 is a genuine improvement; the TTO denominator is reported; and the Data Availability Statement now meets journal requirements with de-duplicated analytic datasets deposited.

My remaining concerns are mostly matters of internal consistency and one omitted limitation, and I believe they can be resolved without new analyses.

Please find below my comments on these issues.

1. Your abstract reports hyperuricemia as 6.04/59.63, whereas Table 3 provides an FAERS ROR of 7.36 (95% CI 2.30–23.55). All other abstract values match Table 3, so this single figure should be corrected.

2. Table 2 (JADER) outcome percentages use a different denominator from the one stated. The outcome counts sum to 393, which is the number of PT events, not the 254 unique patients given in the table note, and each percentage is consistent with a denominator of 393. Table 1 (FAERS) outcomes, by contrast, sum to 520 and use the patient denominator. Please harmonize the two tables and correct the note. I also noted that the JADER "Recovered" count of 147 is identical to the female count of 147; please check the source cell and correct it.

3. In your response to Reviewer 3, comment 3.6, you explain the event-level choice clearly, but the Limitations section does not mention non-independence. Please add a sentence acknowledging it. Also, please state explicitly in the Limitations that fusion status could not be verified and that the reference population is not molecularly comparable, and consider softening the ROS1 framing in the abstract accordingly.

Thank you.

Reviewer #5: Very Good! All comments have been addressed. The analysis of Entrectinib across both pharmacovigilance databases, FAERS and JADER, has been well executed, including four disproportionality analyses: ROR, PRR, BCPNN, and MGPS. The multi-layered analysis of the drug at both SOC and PT levels, age-subgroup analysis, sex-subgroup analysis, and Time-to-onset analysis and now new revisions have strengthened this study.

Reviewer #6: The authors have appropriately addressed the comments. I don't have additional questions and wish the authors best of luck to their future research endeavors.

**********

what does this mean?). If published, this will include your full peer review and any attached files.

If you choose “no”, your identity will remain anonymous but your review may still be made public.

**Do you want your identity to be public for this peer review?**  For information about this choice, including consent withdrawal, please see our Privacy Policy

Reviewer #1: **Yes:** Dr. Oluwafolayemi Doyeni

Reviewer #2: **Yes:** Juan Carlos Alvarado Gonzalez MD, MSc.

Reviewer #3: No

Reviewer #5: **Yes:** SABREENA ALAM

Reviewer #6: **Yes:** Yuhang Liu

**********

[NOTE: If reviewer comments were submitted as an attachment file, they will be attached to this email and accessible via the submission site. Please log into your account, locate the manuscript record, and check for the action link "View Attachments". If this link does not appear, there are no attachment files.]

To ensure your figures meet our technical requirements, please review our figure guidelines: https://journals.plos.org/plosone/s/figures

You may also use PLOS’s free figure tool, NAAS, to help you prepare publication quality figures: https://journals.plos.org/plosone/s/figures#loc-tools-for-figure-preparation.

NAAS will assess whether your figures meet our technical requirements by comparing each figure against our figure specifications.

# Author comment: Author response to Decision Letter 2

**DOI:** 10.1371/journal.pone.0358331.r004

9 Aug 2026

Dear Reviewers,

On behalf of all co-authors, we sincerely thank the editor and reviewers for their careful, constructive, and encouraging evaluation of our revised manuscript entitled “Safety profile of entrectinib in NSCLC: multi-source pharmacovigilance analysis using FAERS and JADER.” We greatly appreciate the reviewers’ recognition of the methodological rigor and substantial improvement of the manuscript. The remaining comments were highly helpful for further improving internal consistency, clarity, clinical interpretation, and presentation.

In response to the current round of review, we rechecked the citations and formatting of all Supporting Information items, standardized abbreviations throughout the manuscript, added percentages alongside absolute event counts for the cross-database replicated PT-level signals in Table 3, and reviewed all figure legends for independent interpretability. We also corrected the hyperuricaemia ROR in the Abstract, clarified the different denominators used for JADER baseline characteristics and clinical outcomes, verified the JADER “Recovered” count, and expanded the Limitations to address event-level non-independence, unavailable fusion status, and the lack of molecular comparability between the entrectinib and reference groups.

Point-by-point responses

Reviewer #2

Comment 2.1. Ensure every supplementary table (S1–S10) is cited sequentially in the Results.

Response: Thank you for this careful suggestion. We rechecked all Supporting Information citations and ensured that the items are introduced in numerical order at their first relevant locations. S1 and S2 Tables are cited in the Methods because they describe the event-level contingency table and the disproportionality algorithms and signal criteria; S3–S8 Tables are cited sequentially in the Results; and S9 and S10 are de-duplicated analytic datasets and are cited in the Data Availability Statement. We retained the designations “S9 Dataset” and “S10 Dataset” because these items are datasets rather than supplementary tables.

Comment 2.2. Verify consistent formatting of abbreviations throughout the manuscript.

Response: Thank you for this helpful comment. We conducted a comprehensive review of abbreviations in the Abstract, main text, tables, and figure legends. We standardized the use and formatting of FAERS, JADER, NSCLC, AE, SOC, PT, ROR, PRR, BCPNN, MGPS, EBGM, TTO, CI, and IQR.

Comment 2.3. Consider reporting percentages alongside absolute event counts whenever PT-level signals are summarized.

Response: Thank you for this useful recommendation. To improve interpretability without overloading the main text with additional numerical detail, we added corresponding percentages alongside absolute event counts for all 23 cross-database replicated PT-level signals in Table 3. The column headings were revised to “n (%).” Percentages were calculated using the total number of PT-level adverse event records in the corresponding database as the denominator (FAERS, n = 1,573; JADER, n = 393). We also clarified in the table note and Figure 4 legend that these percentages describe the distribution of reported PT events and should not be interpreted as incidence rates or patient-level risks.

Comment 2.4. Review figure legends to ensure they can be interpreted independently of the main text.

Response: We agree and carefully reviewed the legends for Figures 1–6. We expanded the legends where additional clarification was needed, particularly for Figures 3–5. The revised legends now describe the contents of each panel, the relevant analysis denominators, the meaning of n (%), the ROR point estimates and 95% confidence intervals, the reference line, truncated confidence intervals, complementary heatmap metrics, subgroup comparison directions, and all relevant abbreviations. These revisions allow the figures to be interpreted without relying on the main text.

Reviewer #3

Comment 3.1. The Abstract reports hyperuricaemia as 6.04/59.63, whereas Table 3 provides an FAERS ROR of 7.36 (95% CI 2.30–23.55).

Response: Thank you for identifying this inconsistency. We corrected the FAERS ROR for hyperuricaemia in the Abstract from 6.04 to 7.36. The revised Abstract now reports hyperuricaemia as 7.36/59.63 for FAERS/JADER, consistent with Table 3.

Comment 3.2. The denominator used for the JADER outcome percentages in Table 2 differs from the denominator stated in the table note. Please harmonize the tables, correct the note, and verify the JADER “Recovered” count of 147.

Response: Thank you for this careful observation. We rechecked the underlying JADER data and confirmed that sex, body weight, age, and reporter type were summarized at the unique-patient level using 254 patients, whereas clinical outcomes were summarized at the PT-event level using 393 PT-level adverse event records. We therefore revised the Table 2 note to explicitly distinguish these denominators. Table 1 remains based on 520 eligible FAERS patients. We also rechecked the source data for the JADER “Recovered” category and confirmed that the count of 147 is correct. Its identical value to the number of female patients is coincidental and does not result from a duplicated or incorrectly referenced source cell.

Comment 3.3. Please acknowledge event-level non-independence in the Limitations, state that fusion status could not be verified and that the reference population was not molecularly comparable, and consider softening the ROS1 framing in the Abstract.

Response: Thank you for this important comment. We revised the Limitations to explicitly state that a single patient or case report could contribute multiple PT events; therefore, event-level observations were not fully independent, and within-report clustering may have influenced the disproportionality estimates. We also added that ROS1 fusion status could not be verified in FAERS or JADER and that the NSCLC reference population could not be considered molecularly comparable to the entrectinib group. In addition, the Abstract now explicitly states that molecular fusion status was not available for verification, thereby avoiding the implication that all included NSCLC reports were molecularly confirmed ROS1-positive cases.

We again thank the editor and reviewers for their rigorous and constructive comments. We believe that these revisions have further improved the internal consistency, transparency, presentation, and interpretive balance of the manuscript.

Yours sincerely,

Ge Sun

On behalf of all authors

Attachment Submitted filename: Response_to_Reviewers_auresp_2.docx

# Peer review: Decision Letter 2

**DOI:** 10.1371/journal.pone.0358331.r005

**Authors:** Qi Chen

31 Aug 2026

Safety profile of entrectinib in NSCLC: multi-source pharmacovigilance analysis using FAERS and JADER

PONE-D-26-26274R2

Dear Dr. Sun,

We’re pleased to inform you that your manuscript has been judged scientifically suitable for publication and will be formally accepted for publication once it meets all outstanding technical requirements.

Within one week, you’ll receive an e-mail detailing the required amendments. When these have been addressed, you’ll receive a formal acceptance letter and your manuscript will be scheduled for publication.

An invoice will be generated when your article is formally accepted. Please note, if your institution has a publishing partnership with PLOS and your article meets the relevant criteria, all or part of your publication costs will be covered. Please make sure your user information is up-to-date by logging into Editorial Manager at Editorial Manager® and clicking the ‘Update My Information' link at the top of the page. For questions related to billing, please contact billing support.

If your institution or institutions have a press office, please notify them about your upcoming paper to help maximize its impact. If they’ll be preparing press materials, please inform our press team as soon as possible -- no later than 48 hours after receiving the formal acceptance. Your manuscript will remain under strict press embargo until 2 pm Eastern Time on the date of publication. For more information, please contact onepress@plos.org.

Kind regards,

Qi Chen, PhD

Academic Editor

PLOS One

Additional Editor Comments (optional):

The authors made good effort to address the reviewers' questions. There is no more comments.

Reviewers' comments:

Reviewer's Responses to Questions

**Comments to the Author**

Reviewer #2: All comments have been addressed

Reviewer #3: All comments have been addressed

Reviewer #5: All comments have been addressed

**********

2. Is the manuscript technically sound, and do the data support the conclusions?

Reviewer #2: Yes

Reviewer #3: Yes

Reviewer #5: Yes

**********

3. Has the statistical analysis been performed appropriately and rigorously?

Reviewer #2: Yes

Reviewer #3: Yes

Reviewer #5: Yes

**********

4. Have the authors made all data underlying the findings in their manuscript fully available?

The PLOS Data policy

Reviewer #2: Yes

Reviewer #3: Yes

Reviewer #5: Yes

**********

5. Is the manuscript presented in an intelligible fashion and written in standard English?

Reviewer #2: Yes

Reviewer #3: Yes

Reviewer #5: Yes

**********

Reviewer #2: The manuscript addresses a clinically relevant topic because real-world safety data for entrectinib remain limited, particularly outside clinical trials. The use of two independent pharmacovigilance databases represents an important strength that improves the robustness of the detected signals.

All comments have been addressed.

Reviewer #3: I thank the authors for their careful and complete responses.

The authors have adequately addressed all points raised in the previous round, and I have no further substantive concerns.

Reviewer #5: All comments have been addressed. The research has been conducted with rigorous methodology. The analysis of Entrectinib across both pharmacovigilance databases, FAERS and JADER, has been well executed, including four disproportionality analyses: ROR, PRR, BCPNN, and MGPS. The multi-layered analysis of the drug at both SOC and PT levels, age-subgroup analysis, sex-subgroup analysis, and Time-to-onset analysis have strengthened this study

**********

what does this mean?). If published, this will include your full peer review and any attached files.

If you choose “no”, your identity will remain anonymous but your review may still be made public.

**Do you want your identity to be public for this peer review?**  For information about this choice, including consent withdrawal, please see our Privacy Policy

Reviewer #2: **Yes:** Juan Carlos Alvarado MD, MSc.

Reviewer #3: No

Reviewer #5: **Yes:** Sabreena Alam

**********

# Editor report: Acceptance letter

**DOI:** 10.1371/journal.pone.0358331.r006

**Authors:** Qi Chen

PONE-D-26-26274R2

PLOS One

Dear Dr. Sun,

I'm pleased to inform you that your manuscript has been deemed suitable for publication in PLOS One. Congratulations! Your manuscript is now being handed over to our production team.

At this stage, our production department will prepare your paper for publication. This includes ensuring the following:

* All references, tables, and figures are properly cited

* All relevant supporting information is included in the manuscript submission,

* There are no issues that prevent the paper from being properly typeset

You will receive further instructions from the production team, including instructions on how to review your proof when it is ready. Please keep in mind that we are working through a large volume of accepted articles, so please give us a few days to review your paper and let you know the next and final steps.

Lastly, if your institution or institutions have a press office, please let them know about your upcoming paper now to help maximize its impact. If they'll be preparing press materials, please inform our press team within the next 48 hours. Your manuscript will remain under strict press embargo until 2 pm Eastern Time on the date of publication. For more information, please contact onepress@plos.org.

You will receive an invoice from PLOS for your publication fee after your manuscript has reached the completed accept phase. If you receive an email requesting payment before acceptance or for any other service, this may be a phishing scheme. Learn how to identify phishing emails and protect your accounts at https://explore.plos.org/phishing.

If we can help with anything else, please email us at customercare@plos.org.

Thank you for submitting your work to PLOS One and supporting open access.

Kind regards,

PLOS One Editorial Office Staff

on behalf of

Dr. Qi Chen

Academic Editor

PLOS One
